# MeCP2 Lactylation Protects against Ischemic Brain Injury by Transcriptionally Regulating Neuronal Apoptosis

**DOI:** 10.1002/advs.202415309

**Published:** 2025-04-24

**Authors:** Min Sun, Yuxin Zhang, Rui Mao, Yan Chen, Pinyi Liu, Lei Ye, Siyi Xu, Junqiu Jia, Shu Shu, Huiya Li, Yanping Yin, Shengnan Xia, Yanting Chen, Yun Xu

**Affiliations:** ^1^ Department of Neurology Nanjing Drum Tower Hospital Affiliated Hospital of Medical School Nanjing University Nanjing 210008 China; ^2^ Nanjing Drum Tower Hospital Clinical College of Nanjing University of Chinese Medicine Nanjing 210008 China; ^3^ State Key Laboratory of Pharmaceutical Biotechnology and Institute of Translational Medicine for Brain Critical Diseases Nanjing University Nanjing 210008 China; ^4^ Jiangsu Key Laboratory for Molecular Medicine and Institute of Translational Medicine for Brain Critical Diseases Nanjing University Nanjing 210008 China; ^5^ Jiangsu Provincial Key Discipline of Neurology Nanjing 210008 China; ^6^ Nanjing Neurology Clinical Medical Center and Nanjing Gulou Hospital Brain Disease and Brain Science Center Nanjing 210008 China; ^7^ Nanjing Drum Tower Hospital Clinical College of Jiangsu University Nanjing 210008 China; ^8^ Department of Neurology Nanjing Drum Tower Hospital Chinese Academy of Medical Science & Peking Union Medical College Nanjing 210008 China; ^9^ Nanjing Key Laboratory for Cardiovascular Information and Health Engineering Medicine Nanjing 210008 China

**Keywords:** ischemic stroke, lactylation, MeCP2 lactylation, neuronal apoptosis, transcriptional regulation

## Abstract

Lactate plays diverse roles in brain pathophysiology, including ischemic stroke. Here, the role of lysine lactylation, an epigenetic modification of lactate, in cerebral ischemia is investigated. Using a mouse model of transient middle cerebral artery occlusion, increased brain lactate levels and global protein lactylation are observed. Proteomics analysis reveals significant lactylation of non‐histone proteins in the ischemic penumbra. Lactylation of MeCP2, a transcriptional regulator, is identified as a protective mechanism against stroke‐induced neuronal death. Inhibition of MeCP2 lactylation through chemical or genetic manipulation increases infarct volume and aggravates neurological deficits. Mechanistically, MeCP2 lactylation at K210/K249 represses the transcription of apoptosis‐associated genes, including *Pdcd4* and *Pla2g6*, thereby attenuating neuronal apoptosis. Additionally, HDAC3 and p300 are identified as key enzymes that regulate MeCP2 lactylation post‐stroke. The findings suggest that MeCP2 lactylation offers a potential therapeutic target for alleviating neuronal damage and improving stroke outcomes.

## Graphical abstract

MeCP2 lactylation protects against ischemic stroke by reducing brain infarct volume and improving neurological outcomes. Lactylation at K210/K249 exerts neuroprotective effects by repressing the transcription of apoptosis‐associated genes, including *Pdcd4* and *Pla2g6*. HDAC3 and p300 serve as key regulatory enzymes for MeCP2 lactylation following stroke.

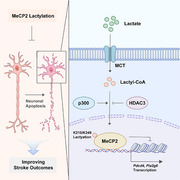

## 1 Introduction

Cerebral ischemia, caused by a significant reduction in blood flow to one or more brain arteries, is a leading cause of morbidity and mortality worldwide.^[^ ^1^ ^]^ The brain uses glucose as its primary source of energy. Glucose produces ATP through two alternative mechanisms: glycolysis and oxidative phosphorylation. Impaired regional cerebral blood flow and glucose metabolism is a hallmark of ischemic injury in acute stroke, resulting from a cascade of events triggered by energy depletion and leading to cell death.^[^ ^2^, ^3^ ^]^ Lactate, once considered a metabolic waste product of glycolysis, is now recognized as a beneficial metabolite in the brain under pathological conditions, including ischemic stroke.^[^ ^4^ ^]^

Brain lactate accumulates markedly after the onset of ischemia, which is clinically critical for identifying the ischemic penumbra with early changes in glucose metabolism.^[^ ^5^, ^6^ ^]^ In experimental stroke models, intracerebroventricular or intravenous injections of lactate after reperfusion improved ischemic infarcts and neurological deficits.^[^ ^7^ ^]^ Emerging evidence suggests that lactate serves as an energy source in the central nervous system (CNS). During an energy crisis, lactate can be transported from astrocytes to neurons as an energy substrate to alleviate energy deficiency in neurons.^[^ ^8^, ^9^ ^]^ Additionally, lactate has essential signaling functions that modulate neuronal functions, including excitability, plasticity, and memory consolidation.^[^ ^4^ ^]^ However, the mechanisms by which lactate regulates these metabolic and cellular functions have not been fully elucidated.

Increasing evidence suggests that alterations in metabolism and epigenetics contribute to disease.^[^ ^10^ ^]^ Chemical modifications are sensitive to changes in intracellular metabolites such as lactate.^[^ ^11^, ^12^ ^]^ Zhang et al. identified lysine lactylation (Kla) as a novel type of histone modification that can be stimulated by lactate.^[^ ^11^ ^]^ Under hypoxic conditions, lactate production and concurrent histone lactylation are induced during macrophage activation, which may promote a shift from inflammatory macrophages toward a more homeostatic phenotype characterized by the expression of reparative genes.^[^ ^13^ ^]^ This suggests that differential histone lactylation is regulated by lactate metabolism, which in turn modulates gene expression. Recent studies have identified histone lactylation as a critical regulatory mechanism in CNS disorders, influencing neuronal activity and neuroinflammation.^[^ ^14^, ^15^ ^]^

In this study, we uncover that lactylation of MeCP2, a non‐histone protein, contributes to neuronal survival during ischemic stroke. With a comprehensive lactylation proteomics, we demonstrated a global change of protein lactylation in response to cerebral ischemia. Notably, we focused on the lactylation of MeCP2, a protein known to play a critical role in neuronal gene regulation. Using in vivo and in vitro stroke models, we identified that MeCP2 lactylation acted as an intrinsic neuroprotective mechanism in stroke. Furthermore, we revealed that MeCP2 lactylation at K210 and K249 enhanced its binding to apoptotic gene promoters, leading to transcriptional repression of pro‐apoptotic genes such as *Pdcd4* and *Pla2g6*, thus protecting against ischemic neuronal death.

## 2 Results

### 2.1 Lactylatic Modification Exerts Neuroprotection in the Ischemic Stroke

**Figure 1:**
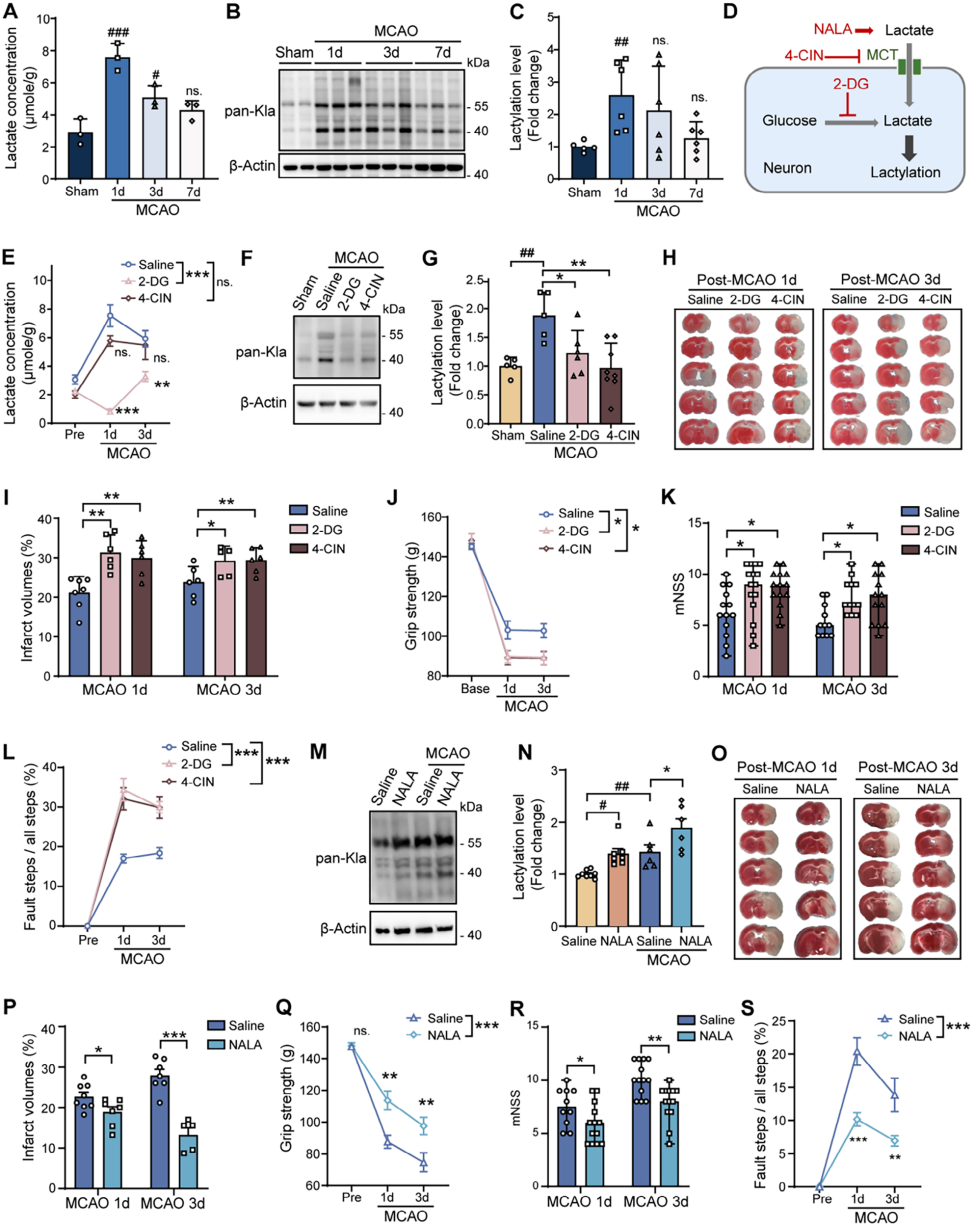
Protein lactylation and its neuroprotective role in ischemic stroke. A) Lactate concentrations in the brains of sham‐operated and MCAO mice at 1, 3, and 7 d after reperfusion (*n* = 3 per group). B) Western blot analysis of pan‐Kla in brain tissues from sham and MCAO mice at 1, 3, and 7 d post‐surgery, representative of three independent experiments. C) Quantification of pan‐Kla levels in the ischemic penumbra of MCAO mice at different time points, normalized to sham controls (*n* = 5–6 per group). D) Schematic diagram illustrating the experimental design, including interventions with 2‐DG, 4‐CIN, and NALA targeting lactate metabolism and lactylation modulation. E) Lactate levels in brain tissues of MCAO mice treated with saline, 2‐DG, or 4‐CIN, measured at 1 and 3 d following reperfusion (*n* = 4–5 per group). F) Western blot showing reduced pan‐Kla in MCAO mice treated with 2‐DG or 4‐CIN compared to saline controls, representative of three independent experiments. G) Quantification of pan‐Kla in brains of MCAO mice treated with saline, 2‐DG, or 4‐CIN, normalized to sham controls (*n* = 5–8 per group). H) Representative TTC staining of brain sections from MCAO mice treated with saline, 2‐DG, or 4‐CIN at 1 and 3 d after reperfusion. I) Quantification of infarct volumes showing aggravated brain damage in 2‐DG and 4‐CIN treated groups (*n* = 5–7 per group). J) Evaluation grip strength, K) modified Neurological Severity Scores (mNSS), and L) fault step ratio in MCAO mice treated with saline, 2‐DG, or 4‐CIN at 1 and 3 d after reperfusion (*n* = 5–15 per group). M) Western blot analysis of pan‐Kla in brain tissues from MCAO mice treated with saline or NALA, representative of three independent experiments. N) Quantification of pan‐Kla levels in brains of MCAO mice treated with saline or NALA, normalized to sham controls (*n* = 6–8 per group). O) Representative TTC staining of brain sections from MCAO mice treated with saline or NALA at 1 and 3 d following reperfusion. P) Quantification of infarct volumes showing alleviated ischemic brain injury in NALA treated groups (*n* = 5–8 per group). Q) Evaluation of grip strength, R) mNSS, and S) fault step ratio in MCAO mice treated with saline or NALA at 1 and 3 d post reperfusion (*n* = 9–13 per group). Modified Neurological Severity Scores (K, R) are shown as median ± ranges, and other values are presented as mean ± SEM. *^#^p* < 0.05, *^##^p* < 0.01, *^###^p* < 0.01 versus sham control; *p < 0.05, **p < 0.01, ***p < 0.001 versus MCAO control; ns, not significant.

Given that lactate serves as a precursor for Kla modification, we first assessed the expression patterns of lactate and global protein lactylation in the ischemic stroke. As expected, it was found an early peak of lactate production in the ischemic hemisphere at 1 d after stroke, but a downward shift was observed at later time points after reperfusion (**Figure** **1**A). Protein Kla was examined by western blot analysis in mouse brain tissues. We found a concurrent accumulation of protein Kla in the penumbra of ischemic brain, with a substantial upregulation at 1 d after reperfusion (Figure 1B,C).

To further confirm the roles of Kla modifications in the setting of stroke, 2‐deoxyglucose (2‐DG) and 4‐hydroxycinnamate (4‐CIN) were used to block glycolysis and lactate shuttle respectively (Figure 1D). It was interesting to find that the treatment of 2‐DG resulted in a pronounced decrease of both lactate production and protein Kla in the ischemic brain (Figure 1E–G). Whereas, blocking lactate transport into neurons using 4‐CIN significantly inhibited pan Kla without affecting total lactate production (Figure 1E–G). Considering neuroprotective properties of lactate, the precursor of Kla modification, we further explore the biological functions of Kla in the animal models of ischemic stroke. We found that Kla blockade exacerbated brain damage of the mice after middle cerebral artery occlusion (MCAO) (Figure 1H,I). In addition, blocking Kla could significantly aggravate neurological deficits after stroke, as indicated by weakened grip strength, higher modified Neurological Severity Scores (mNSS), and increased fault step ratios in the 2‐DG and 4‐CIN groups compared with the controls (Figure 1J–L).

Conversely, sodium lactate (NALA) treatment, which mimics lactate, significantly increased pan‐Kla levels and resulted in smaller infarct volumes in MCAO mice (Figure 1M–P). The NALA‐treated stroke mice also showed better functional outcomes compared with the controls, with improved grip strength, lower mNSS scores, and reduced fault step ratios (Figure 1Q–S). These findings suggest that protein lactylation protects against ischemic brain injury, and its modulation can significantly improve stroke outcomes.

### 2.2 Alteration of Protein Lactylation in Response to Cerebral Ischemia

**Figure 2:**
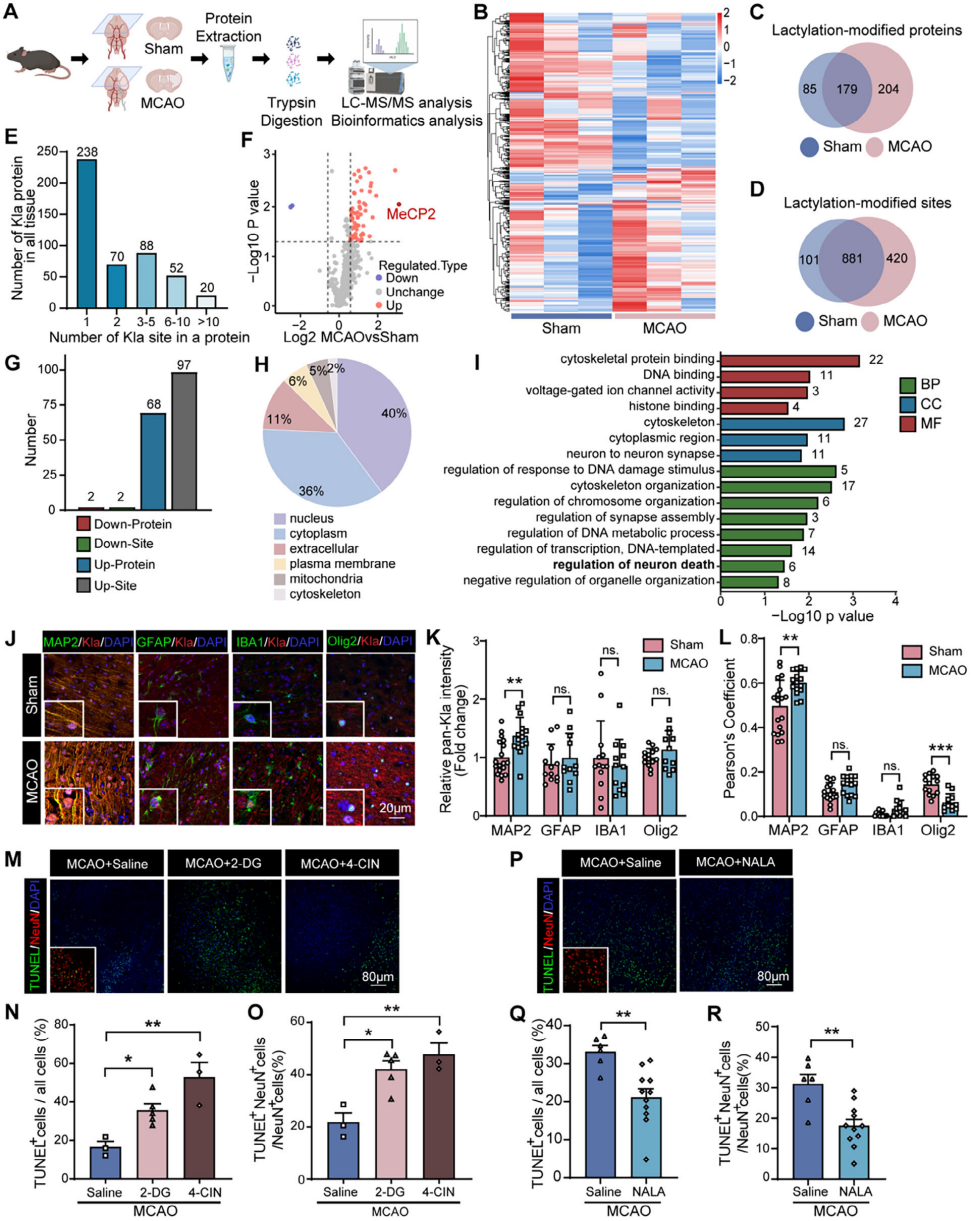
Protein lactylation protects against neuronal apoptosis in stroke. A) Schematic of experimental workflow: Protein extraction from brain tissues of sham and MCAO mice, followed by trypsin digestion and LC‐MS/MS analysis (Created with bioRender.com). B) Heatmap depicting differential lactylation‐modified proteins between sham and MCAO groups. C) Venn diagram showing the overlap of lactylated proteins in sham and MCAO groups. D) Venn diagram showing the overlap of lactylated sites in sham and MCAO groups. E) Distribution of Kla‐modified proteins by the number of lactylation sites. F) Volcano plot showing differentially regulated proteins in MCAO versus sham mice. G) Bar graph showing the distribution of upregulated and downregulated lactylation sites and proteins in the penumbra of MCAO mice. H) Venn diagram illustrating subcellular localization of the differentially lactylated proteins in the penumbra of MCAO mice. I) GO enrichment analysis for BP, CC, and MF associated with stroke‐induce differentially lactylated proteins. J) Representative immunofluorescence images showing co‐localization of Kla (red) with MAP2, GFAP, IBA1, or Olig2 (green) in the cortex of sham and MCAO mice. K) Quantification of Kla intensity in MAP2^+^, GFAP^+^, IBA1^+^, and Olig2^+^ cells in sham and MCAO brain sections (*n* = 11–18 slices from 4–6 mice per group). L) Pearson's correlation coefficient between Kla intensity and the intensity of MAP2, GFAP, and IBA1(*n* = 11–18 slices from 4–6 mice per group). M) Representative TUNEL staining of the brain sections of MCAO mice treated with saline, 2‐DG, or 4‐CIN at 1 d after reperfusion. N) Quantification of the proportion of TUNEL^+^ cells among all cells in MCAO brain sections treated with saline, 2‐DG, or 4‐CIN (*n* = 3–5 mice per group). O) Quantification of the percentage of TUNEL^+^ neurons among all neuronal cells in MCAO brain sections treated with saline, 2‐DG, or 4‐CIN (*n* = 3–5 mice per group). P) Representative TUNEL staining in MCAO brain sections treated with saline or NALA at 1 d following reperfusion. Q) Quantification of the proportion of TUNEL^+^ cells among all cells in MCAO brain sections treated with saline or NALA (*n* = 6–11 mice per group). R) Quantification of the percentage of TUNEL^+^ neurons among all neuronal cells in MCAO brain sections treated with saline or NALA (*n* = 6–11 mice per group). Data are presented as mean ± SEM. *p < 0.05, **p < 0.01 versus MCAO control; ns, not significant.

Based on these findings, we sought to identify and quantify Kla modifications in brain proteins that were remarkably affected by cerebral ischemia. Accordingly, we analyzed the ischemic penumbra of the MCAO mice and control mice using mass spectrometry. Kla‐containing peptides were enriched with immobilized anti‐Kla and analyzed using liquid chromatography‐tandem mass spectrometry (LC‐MS/MS) (**Figure** **2**A). A total of 1402 Kla sites across 468 proteins were identified, with significant differences in lactylation‐modified proteins and sites between the sham and MCAO groups (Figure 2B–D). Among these Kla proteins, 238 (36.9%) modified proteins had a single Kla site, and 72 (15.4%) had more than five Kla sites (Figure 2E).

We next quantified the changes of protein Kla level in response to MCAO treatment relative to the total protein abundance in the ischemic penumbra. The cutoff ratio for significant Kla changes between MCAO and sham mice was set to above 1.5 or below 0.67. The results showed that abundance of 97 Kla sites in 68 proteins were up‐regulated, while only 2 Kla sites in 2 proteins were down‐regulated in MCAO mice compared with sham mice (Figure 2F,G, Table S1, Supporting Information). The up‐regulated Kla proteins were mainly located in the cytoplasm, nucleus, plasma membrane and mitochondria (Figure 2H, Table S1, Supporting Information). Among these stroke‐regulated Kla proteins, 14 proteins possessed multiple Kla sites (2‐5 Kla sites, Table S2, Supporting Information). Network‐based gene ontology (GO) analysis was used to cluster all differentially lactylated proteins according to the categories of biological process (BP), molecular function (MF), and cellular components (CC). GO analysis revealed that Kla proteins are involved in regulating various biological processes, including cytoskeleton organization, regulation of DNA damage response, chromosome organization, negative regulation of organelle organization, DNA metabolic processes, synapse assembly, neuronal death, and transcription (DNA‐templated) (Figure 2I). MF enrichment analysis revealed that the Kla proteins were associated with cytoskeletal protein binding, DNA binding, voltage‐gated ion channel activity, and histone binding (Figure 2I). The CC analysis indicated that these Kla proteins were mainly localized in the nuclear lumen, cytoskeleton, cytoplasmic region, and neuron‐to‐neuron synapses (Figure 2I).

### 2.3 Cell‐Type Specificity of Protein Lactylation in Ischemic Stroke

To investigate stroke‐induced changes in pan‐lactylation across different brain cell types, we performed immunofluorescence co‐staining of pan‐Kla with markers of neurons (MAP2 or NeuN), astrocytes (GFAP or S100β), microglia (IBA1), and oligodendrocytes (Olig2 or MBP). In the cortex of sham mice, Kla was predominantly expressed in neuronal nuclei and processes (Figure 2J, Figure S2B, Supporting Information). Minimal colocalization of pan‐Kla with GFAP, IBA1, or Olig2 was observed, indicating baseline lactylation in a subset of astrocytes, microglia and oligodendrocytes (Figure 2J). Post‐MCAO, a pronounced induction of pan‐Kla was observed in neurons within the penumbra, whereas lactylation levels in GFAP‐positive astrocytes, IBA1‐positive microglia and Olig2‐positive oligodendrocytes remained unchanged compared with those in the sham group (Figure 2J,K). Further quantification using Pearson's coefficient confirmed that protein lactylation predominantly occurs in neurons following cerebral ischemia, with a slight increase noted in co‐localization with the astrocytic marker S100β (Figure 2L, Figure S2D, Supporting Information). Similarly, brain tissues from patients with ischemic stroke exhibited evident colocalization of Kla and MAP2 staining (Figure S2E, Supporting Information), highlighting the potential involvement of Kla in the neuronal response to ischemic injury.

To further delineate the cell‐type specificity of protein lactylation following MCAO, flow cytometry was performed. Cells were labeled with specific markers for neurons (NVAM‐1), astrocytes (GLAST), and microglia (CD45 and CD11b) and assessed for Kla expression. Flow cytometry plots revealed distinct populations of Kla‐positive cells across these cell types, with a significantly higher percentage observed in neurons and astrocytes than in microglia (Figure S2F, Supporting Information). Quantitative analysis demonstrated that neurons exhibited the highest levels of lactylation post‐MCAO, suggesting a predominant role for lactylation in these cell types in response to ischemic injury (Figure S2G, Supporting Information). Collectively, these observations indicate that protein lactylation is primarily localized in neurons in the brain following MCAO, reflecting a cell type specific response to ischemic stress.

### 2.4 Lactylation Links Lactate Metabolism with Neuronal Death in Ischemic Stroke

To confirm the effects of different lactylation levels on neuronal cell death following MCAO, we conducted flow cytometry. Neurons were identified using the neuronal marker NCAM‐1, and cell death was assessed using a viability marker. Flow cytometry data revealed a significant increase in the proportion of neuronal death in the MCAO mice receiving 2‐DG or 4‐CIN than those of saline‐treated group (Figure S2H,I, Supporting Information). These findings suggest that the reduced lactylation levels, induced by 2‐DG and 4‐CIN, exacerbate neuronal cell death following ischemic injury. Conversely, higher lactylation levels, as seen with NALA treatment, are associated with reduced neuronal cell death. This underscores the potential protective role of lactylation in neuronal survival, with its reduction correlating with increased neuronal cell death post‐MCAO.

Terminal deoxynucleotidyl transferase dUTP nick end labeling (TUNEL) staining is a marker of DNA fragmentation, indicating apoptosis. Immunofluorescence analysis using TUNEL and NeuN co‐staining further revealed that treatment with 2‐DG and 4‐CIN resulted in increased neuronal death in the MCAO group (Figure 2M). Quantification of TUNEL‐positive cells showed significant increases in both total and neuronal cell death in the 2‐DG and 4‐CIN treated groups compared to the saline control (Figure 2N,O). In contrast, NALA treatment markedly mitigated stroke‐induced neuronal apoptosis (Figure 2P–R). These findings highlight the potential regulatory function of protein lactylation in mitigating neuronal apoptosis post‐MCAO. Meanwhile, Western blot analysis revealed that cleaved caspase‐1 levels, a marker of pyroptosis, was not induced at 1 and 3 d post‐MCAO (Figure S2J,K, Supporting Information). However, phosphorylated RIP3 (p‐RIP3), a marker of necroptosis, was significantly upregulated at 1 and 3 d post‐MCAO (Figure S2J,L, Supporting Information). Interventions targeting lactylation, such as treatments with NALA, did not significantly affect the levels of p‐RIP3 (Figure S2M,N, Supporting Information). These findings indicate that lactylation primarily influences neuronal apoptosis rather than pyroptosis or necroptosis in response to ischemic injury.

### 2.5 Protein Lactylation Reduces Neuronal Apoptosis Following Oxygen‐Glucose Deprivation/Reoxygenation

To further investigate the role of lactylation in modulating neuronal death pathways under ischemic conditions, we utilized an oxygen‐glucose deprivation/reoxygenation (OGD/R) model in primary neuronal cultures. Western blot analysis was performed to assess cleaved caspase‐3 levels, an indicator of apoptosis, at different time points post‐reoxygenation. We found that the expression of cleaved Caspase‐3 was markedly increased at 6 h post‐reoxygenation compared with that in the control group (Figure S3A,B, Supporting Information). To explore the effects of lactylation blockade on cell viability and apoptosis under ischemic conditions, we treated primary neurons with varying concentrations of 2‐DG. We then assessed cell viability using cell counting kit 8 (CCK‐8) assays and analyzed apoptosis through western blotting. The CCK‐8 assay demonstrated that treatment with 2‐DG led to a dose‐dependent decrease in cell viability, with significant reductions observed at concentrations of 4 mm and higher (Figure S3C, Supporting Information). Furthermore, we found that 4 mm concentration of 2‐DG reduced protein lactylation levels, accompanied by a significant induction of cleaved caspase‐3 under OGD/R conditions (Figure S3D–F, Supporting Information). Conversely, NALA treatment significantly improved cell viability in a dose‐dependent manner (Figure S3G, Supporting Information), and the 10 mm concentration of NALA was selected for further experiments. As expected, treatment with NALA significantly upregulated pan‐Kla levels and reduced neuronal apoptosis after OGD/R (Figure S3H–J, Supporting Information), suggesting a protective role of lactylation in maintaining cell survival. These results reveal that lactylation plays a crucial role in regulating neuronal cell death and viability under ischemic conditions. Lower lactylation levels, induced by 2‐DG, exacerbate apoptosis, while treatments that enhance lactylation, such as NALA, confer neuroprotection by reducing cell death and promoting cell survival.

### 2.6 Stroke Induces Lactylation of Non‐Histone Proteins Associated with Neuronal Death

**Figure 3:**
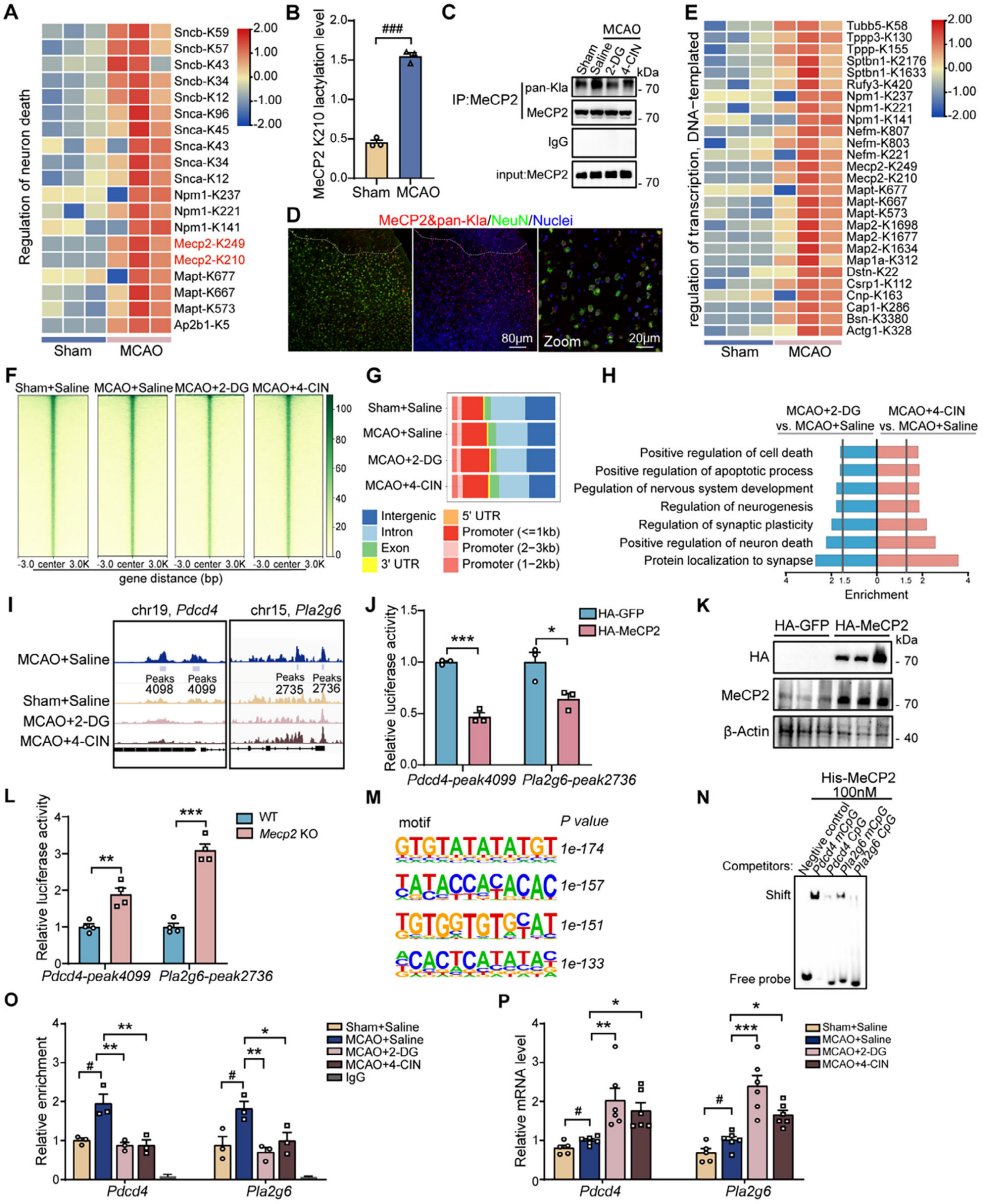
MeCP2 lactylation regulates neuronal apoptosis after stroke. A) Heatmap depicting differential lactylation at specific lysine residues of proteins involved in the regulation of neuronal death after stroke. B) PRM quantification of MeCP2 K210 lactylation levels in the brains of sham and MCAO mice (*n* = 3 mice per group). C) MeCP2 was immunoprecipitated from sham and MCAO brain tissues, and pan‐Kla and MeCP2 levels were analyzed by Western blot. D) Representative immunofluorescence images showing the proximity ligation assay used to validate MeCP2 lactylation in neurons within the cortex of MCAO mice. Red, in situ PLA detection of MeCP2 and pan‐Kla; green, NeuN; blue, DAPI. E) Heatmap showing differentially lactylated proteins involved in transcriptional regulation between sham and MCAO groups. F) The binding density of MeCP2 was visualized by deepTools: the heatmap presents the CUT&Tag counts at different MeCP2 binding peaks across different treatment groups, ordered by signal strength. G) Distribution of MeCP2 binding peaks across genomic regions in cortical tissues from sham‐operated mice and MCAO mice treated with saline, 2‐DG or 4‐CIN. H) GO enrichment analysis of downregulated and upregulated genes associated with MeCP2 binding sites in the penumbra from MCAO mice. I) Genome browser tracks of MeCP2 CUT&Tag peaks at *Pdcd4* and *Pla2g6* loci across treatment groups. The purple rectangles indicate the peak regions of MeCP2 on target‐gene promoters. J) Luciferase reporter assay showing relative activity at *Pdcd4* and *Pla2g6* peaks in HEK293T cells expressing HA‐MeCP2 or HA‐GFP (*n* = 3 per group). K) Western blot showing over‐expression of MeCP2 in HEK293 cells, with β‐actin as a loading control. L) Relative luciferase activity at *Pdcd4* and *Pla2g6* peaks in wild‐type (WT) and MeCP2^−/−^ HEK293T cells (*n* = 4 per group). M) Motif enrichment analysis of MeCP2 binding sites, highlighting top enriched motifs with corresponding *p*‐values. N) EMSA demonstrating MeCP2 binding to the CpG‐rich promoters of the target genes *Pdcd4* and *Pla2g6*. O) ChIP‐qPCR quantification of MeCP2 enrichment at *Pdcd4* and *Pla2g6* promoter in sham mice and MCAO mice treated with saline, 2‐DG or 4‐CIN (*n* = 4 per group). P) Cortical expression of *Pdcd4* and *Pla2g6* determined by qPCR in sham mice and MCAO mice treated with saline, 2‐DG or 4‐CIN (*n* = 5–6 per group). Data are presented as mean ± SEM. *^#^p* < 0.05 versus sham control; *p < 0.05, **p < 0.01, ***p < 0.001 versus MCAO control; ns, not significant.

Considering protein lactylation critically contributes to neuronal death cascade of ischemic stroke, we further dissected stroke‐regulated Kla proteins from neuronal death associated BP (BP‐GO:1901214, *p* = 0.03). Heatmap analysis revealed significant changes in lactylation of non‐histone proteins involved in the regulation of neuronal death following stroke, including MeCP2, AP‐2 complex subunit beta (Ap2b1), microtubule‐associated protein tau (Mapt), nucleophosmin (Npm1), alpha‐synuclein (Snca), and beta‐synuclein (Sncb) (**Figure** **3**A). In an effort to validate these candidate Kla markers in MCAO mice, we applied parallel reaction monitoring (PRM) to quantify potential lactylated proteins in sham and MCAO mice. We confirmed that lactylation of MeCP2 at lysine 210 (K210), Npm1 at K221, and Snca at K96 were significantly up‐regulated in MCAO mice (Figure 3B, Figure S4A, Supporting Information). Thus, these findings suggest that lactylation may act as a regulatory nexus for neuronal death in ischemic stroke.

MeCP2 is known to play a critical role in modulating neuronal death.^[^ ^16^, ^17^ ^]^ To further explore the role of MeCP2 in the response to ischemic injury, we analyzed its temporal expression in the ischemic cortex of MCAO mice. We observed that MeCP2 expression remained relatively stable in the early phase following cerebral ischemia (Figure S4B–E, Supporting Information). Additionally, MeCP2 was predominantly localized in the nucleus, and its nuclear levels did not exhibit significant alterations following ischemic stroke (Figure S4F–G, Supporting Information). With LC/MS analysis, we identified eight lactylation sites on MeCP2 lysine residues, namely K210, K311, K249, K256, K266, K271, K200, and K447, in the penumbra area of mice 24 h after MCAO (Figure S5A–H, Supporting Information). Specifically, stroke induced significant lactylation of MeCP2 at the K210 and K249 sites, which were involved in several top‐enriched biological processes in acute cerebral ischemia (Tables S1 and S3, Supporting Information). Therefore, we hypothesized that Kla modification of MeCP2 might play a role in regulating neuronal death in acute ischemic stroke. Co‐immunoprecipitation (Co‐IP) assays revealed upregulation of MeCP2 lactylation in MCAO mice, which was significantly reduced by treatment with either 2‐DG or 4‐CIN (Figure 3C). Additionally, immunofluorescence staining of cortical sections using NeuN and proximity ligation assay (PLA) markers demonstrated the co‐localization of MeCP2 lactylation with neuronal cells, confirming that this modification predominantly occurs in neurons (Figure 3D).

### 2.7 Lactylation of MeCP2 Transcriptionally Modulates Neuronal Death in Stroke

Furthermore, heatmap analysis showed significant changes in the lactylation levels of key transcription‐related proteins including MeCP2 in the MCAO group compared to the sham group, indicating MeCP2 as a transcriptional regulator under ischemic condition (Figure 3E). To further investigate this, we utilized the cleavage under targets and tagmentation (CUT&Tag) to explore the of MeCP2 role in transcriptional regulation post‐stroke. As shown in Figure 3F, MeCP2‐binding peaks were predominantly enriched at transcription start sites (TSS). The distribution of MeCP2 binding sites across genomic regions revealed a predominance in promoter regions, with notable differences observed under various treatment conditions (Figure 3G). GO enrichment analysis of MeCP2 target genes revealed that lactylation inhibition by 2‐DG or 4‐CIN led to the enrichment of differentially regulated genes in biological processes related to neuronal death, apoptosis, synaptic plasticity, and protein localization to the synapse, highlighting a consistent role of lactylation in regulating neuronal death and synaptic function (Figure 3H; Table S4, Supporting Information). We then examined the differential peaks associated with the apoptotic pathway, and found increased MeCP2 binding in the promoter regions of *Pdcd4* and *Pla2g6* post‐MCAO, which could be reduced by either 2‐DG or 4‐CIN treatment (Figure 3I).

To illustrate the role of MeCP2 in regulating the promoter regions of apoptosis‐related genes, we performed reporter gene assays in HEK293T cells. We observed that MeCP2 over‐expression resulted in a significant decrease in luciferase activity for *Pdcd4* peak4099 and *Pla2g6* peak2736 compared to the control (Figure 3J,K). These findings indicate that MeCP2 can bind to the promoters of genes related to the apoptotic pathway, such as *Pdcd4* and *Pla2g6*, exerting transcriptional repressive effects. To further validate this, we generated *Mecp2* knockout (KO) HEK293T cell lines using the CRISPR/Cas9 system. As shown in Figure S7B (Supporting Information), the target sequence within exon 3 of the MeCP2 gene was disrupted in two KO clones. Both clones exhibited the successful deletion of MeCP2, as evidenced by the absence of MeCP2 protein in KO lines (Figure S7C, Supporting Information). Subsequent luciferase assays in *Mecp2* KO cells demonstrated a significant increase in luciferase activity at the identified binding peaks (Figure 3L). The motif analysis identified high *P*‐value binding sequences, suggesting strong affinity sites for MeCP2 within the promoter regions of *Pdcd4* and *Pla2g6* (Figure 3M). Electrophoretic mobility shift assays (EMSA) demonstrated that both mouse and human recombinant MeCP2 proteins could bind to the methylated probes (Figure 3N, Figure S7F, Supporting Information). These results indicate that MeCP2 binds to specific sequences in the promoter regions of *Pdcd4* and *Pla2g6*.

To further investigate the role of MeCP2 in promoter regulation, we designed constructs with different promoter regions linked to Renilla luciferase (Rluc) reporters including a full promoter (Full), a promoter with the MeCP2 binding peak (Peak), and a promoter lacking the peak (ΔPeak) (Figure S7G, Supporting Information). Luciferase assays demonstrated that the Δpeak4099 and Δpeak2736 construct exhibited significantly lower luciferase activity compared to the full promoter constructs (Figure S7H,I, Supporting Information). This suggests that the peak regions (peak4099 for *Pdcd4* and peak2736 for *Pla2g6*) are essential for MeCP2‐mediated transcriptional regulation of these genes. Chromatin immunoprecipitation‐quantitative polymerase chain reaction (ChIP‐qPCR) analysis revealed significant differences in MeCP2 binding to the peak regions of *Pdcd4* and *Pla2g6* promoters under varying degrees of lactylation. The MCAO mice treated with saline demonstrated significantly higher binding levels than did the sham group. However, treatment with 2‐DG and 4‐CIN, which reduces lactylation levels, resulted in a marked decrease in MeCP2 binding to these regions (Figure 3O). Additionally, qPCR analysis further confirmed the impact of lactylation on downstream gene expression. In the MCAO group treated with saline, the expression level of both *Pdcd4* and *Pla2g6* was significantly down‐regulated compared with that in the control group. However, blocking lactylation led to higher expression levels of these genes compared to the MCAO group (Figure 3P). These observations suggest that MeCP2 lactylation enhances its binding affinity to target gene promoters, and transcriptionally inhibits the expression of genes involved in neuronal apoptosis.

### 2.8 PDCD4 and GVI PLA2 Contributes to Neuronal Apoptosis after Stroke

To investigate the role of PDCD4 and GVI PLA2 in ischemic stroke, we performed Western blot analysis on cortical tissue collected at 1, 3, and 7 d post‐MCAO. Our results showed that PDCD4 expression remained unchanged within 3 d, with a remarkable induction at 7 d post‐MCAO (Figure S8A,B, Supporting Information). Moreover, immunofluorescence staining revealed that PDCD4 expression in NeuN‐positive neurons was significantly elevated as early as 24 h post‐MCAO, indicating a rapid upregulation of neuronal PDCD4 in response to ischemic stress (Figure S8C,D, Supporting Information).

Whereas, GVI PLA2 expression gradually increased over time following stroke (Figure S9A,B, Supporting Information). In vitro, we further examined the impact of overexpressing GVI PLA2 on neuronal apoptosis. Western blot analysis revealed that GVI PLA2 overexpression (OE) led to an increase in cleaved caspase‐3 levels, indicating enhanced apoptosis (Figure S9E,F, Supporting Information). Moreover, treatment with BEL, a selective inhibitor of GVI PLA2, significantly reduced the expression of GVI PLA2 and CHOP, a marker of endoplasmic reticulum stress, under OGD/R conditions (Figure S9G–I, Supporting Information). These findings suggest that both PDCD4 and GVI PLA2 are upregulated following ischemic stroke, with GVI PLA2 contributing to increased neuronal apoptosis and endoplasmic reticulum stress under ischemic conditions.

### 2.9 Lactylation of MeCP2 at K210 and K249 Transcriptionally Inhibits *Pdcd4* and *Pla2g6*

**Figure 4:**
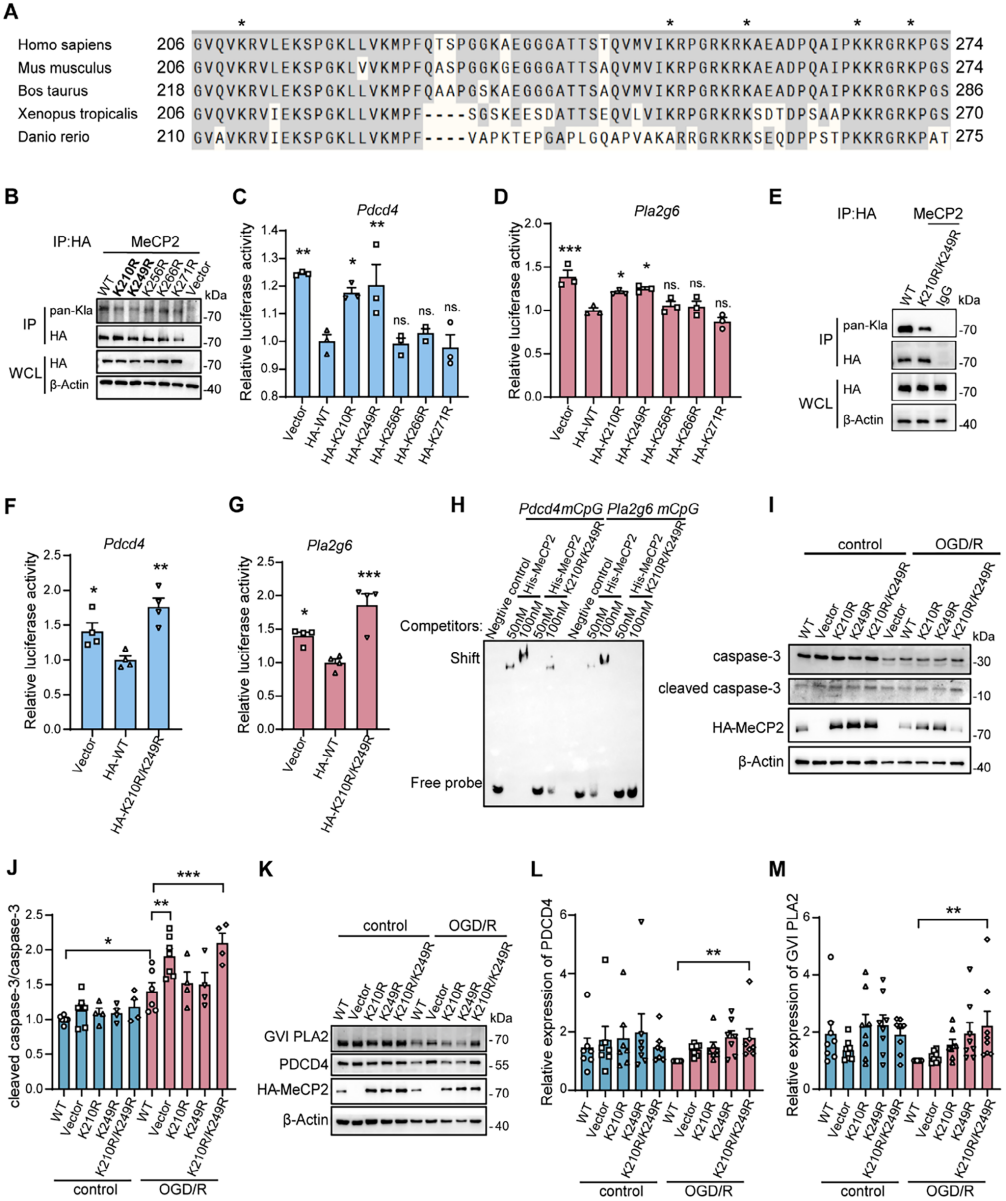
Lactylation of MeCP2 at K210 and K249 regulates apoptotic gene expression in stroke. A) Sequence alignment of the transcriptional repression domain of MeCP2 across species, highlighting conserved MeCP2 lactylation sites. B) Co‐immunoprecipitation and Western blot analysis showing reduced MeCP2 lactylation levels upon K210R or K249R mutation. Luciferase reporter assays showing increased transcriptional activity at the C) *Pdcd4* and D) *Pla2g6* promoters in HEK293T cells expressing K210R or K249R MeCP2 mutants compared to wild‐type MeCP2 (*n* = 3 per group). E) MeCP2 was immunoprecipitated with anti‐HA from HEK293T cells expressing WT or K210R/K249R mutants, and pan‐Kla and HA‐MeCP2 levels were detected by Western blot. Luciferase reporter assays showing transcriptional activity at the F) *Pdcd4* and G) *Pla2g6* promoters in HEK293T cells expressing WT MeCP2 or K210/K249 mutants (*n* = 4 per group). H) EMSA analysis showing recombinant wild‐type and mutant MeCP2 binding to the CpG‐rich promoters of *Pdcd4* and *Pla2g6*. I,J) Western blot analysis of cleaved caspase‐3 levels in primary neurons expressing wild‐type MeCP2, or MeCP2 mutants under OGD/R conditions (*n* = 4–7 per group). K–M) Western blot analysis of GVI PLA2 and PDCD4 protein levels in neurons expressing wild‐type MeCP2, or MeCP2 mutants under OGD/R conditions (*n* = 7–8 per group). Data are presented as mean ± SEM. *p < 0.05, **p < 0.01, ***p < 0.001; ns, not significant.

MeCP2 contains four domains: the transcriptional repression domain (TRD) recruits co‐repressors to silence transcription, the methyl‐CpG‐binding domain (MBD) binds to methylated CpGs, the N‐terminal domain (NTD) interacts with heterochromatin protein 1, and the C‐terminal domain (CTD) aids in binding to DNA and the nucleosome. We performed structure‐binding experiments by expressing HA‐tagged full length or truncated MeCP2 in *Mecp2* KO HEK293T cells (Figure S10A, Supporting Information). It was worthy to note that MeCP2 lactylation predominantly occurred at the TRD domain (Figure S10B, Supporting Information). To confirm the functional significance of MeCP2 lactylation, we analyzed the sequence alignment of the TRD of MeCP2 across multiple species, and identified conserved lactylation sites along with their surrounding regions (**Figure** **4**A). Co‐IP assays demonstrated that mutations at either K210 or K249 significantly reduced MeCP2 lactylation levels, underscoring the critical importance of these sites for this post‐translational modification (Figure 4B). Subsequent luciferase reporter assays showed that K210R and K249R MeCP2 mutations led to a remarkable increase in *Pdcd4* and *Pla2g6* transcription compared to wild‐type MeCP2 group, suggesting lactylation at these sites is essential for the transcriptional repression of these genes (Figure 4C,D). The double‐point mutation K210R/K249R in MeCP2 significantly diminished its lactylation and corresponding transcriptional repression of *Pdcd4* and *Pla2g6* (Figure 4E–G). Furthermore, recombinant MeCP2 exhibited a strong binding to both *Pdcd4* and *Pla2g6* promoters, with a dose‐dependent increase in band intensity observed at 50 and 100 nm concentrations (Figure 4H). However, the K210R/K249R mutant of MeCP2 displayed a markedly reduced binding affinity for both promoters (Figure 4H), highlighting the role of lactylation in enhancing its DNA binding capacity.

In addition, we assessed the impact of these mutations on neuronal apoptosis under OGD/R conditions. Primary neuronal cultures transfected with the K210R/K249R mutant exhibited increased levels of cleaved caspase‐3 compared with the WT groups (Figure 4I,J). These findings suggest that mutations at K210R/K249R in MeCP2 enhance the susceptibility of neuronal cells to apoptosis under OGD conditions. Further investigation into the expression of GVI PLA2 and PDCD4, downstream targets involved in apoptosis regulation, showed that the protein levels of both GVI PLA2 and PDCD4 were upregulated in neurons expressing the MeCP2 K210R/K249R double mutant under OGD conditions (Figure 4K–M). These findings suggest that lactylation of MeCP2 at K210 and K249 enhances its ability to bind to methyl‐CpG‐rich regions in the promoters of *Pdcd4* and *Pla2g6*, thereby modulating the expression of these genes and protecting against ischemia‐induced neuronal apoptosis.

### 2.10 Temporal Dynamics and Pharmacological Modulation of MeCP2 Lactylation at K210 and K249 Post‐Ischemia

To explore the role of MeCP2 lactylation in ischemic stroke, we examined the levels of MeCP2 lactylation at K210 and K249 in the MCAO mouse. Western blot analysis revealed a significant increase in MeCP2 lactylation levels at both K210 and K249 in the ischemic cortex at 1 d post‐MCAO compared to sham controls (Figure S13A–C, Supporting Information). These lactylation levels declined at 3 and 7 d post‐MCAO, returning to near baseline levels, indicating a transient increase in MeCP2 lactylation early after ischemic injury.

We further assessed the effect of metabolic interventions on MeCP2 lactylation sites by treating MCAO mice with 2‐DG or 4‐CIN. Both treatments significantly reduced the MeCP2 lactylation level at K210 and K249 compared to saline‐treated MCAO mice, suggesting that both glycolysis inhibition and lactate transport blockade can reduce ischemia‐induced lactylation of MeCP2 (Figure S13D–F, Supporting Information). These findings suggest that MeCP2 lactylation at K210 and K249 is dynamically regulated following ischemic stroke, with a rapid increase observed during the acute phase. Moreover, the reduction in MeCP2 lactylation by 2‐DG and 4‐CIN indicates that metabolic alterations can modulate this epigenetic modification.

### 2.11 Neuronal MeCP2 Lactylation at K210 and K249 Improves Stroke Outcomes

**Figure 5:**
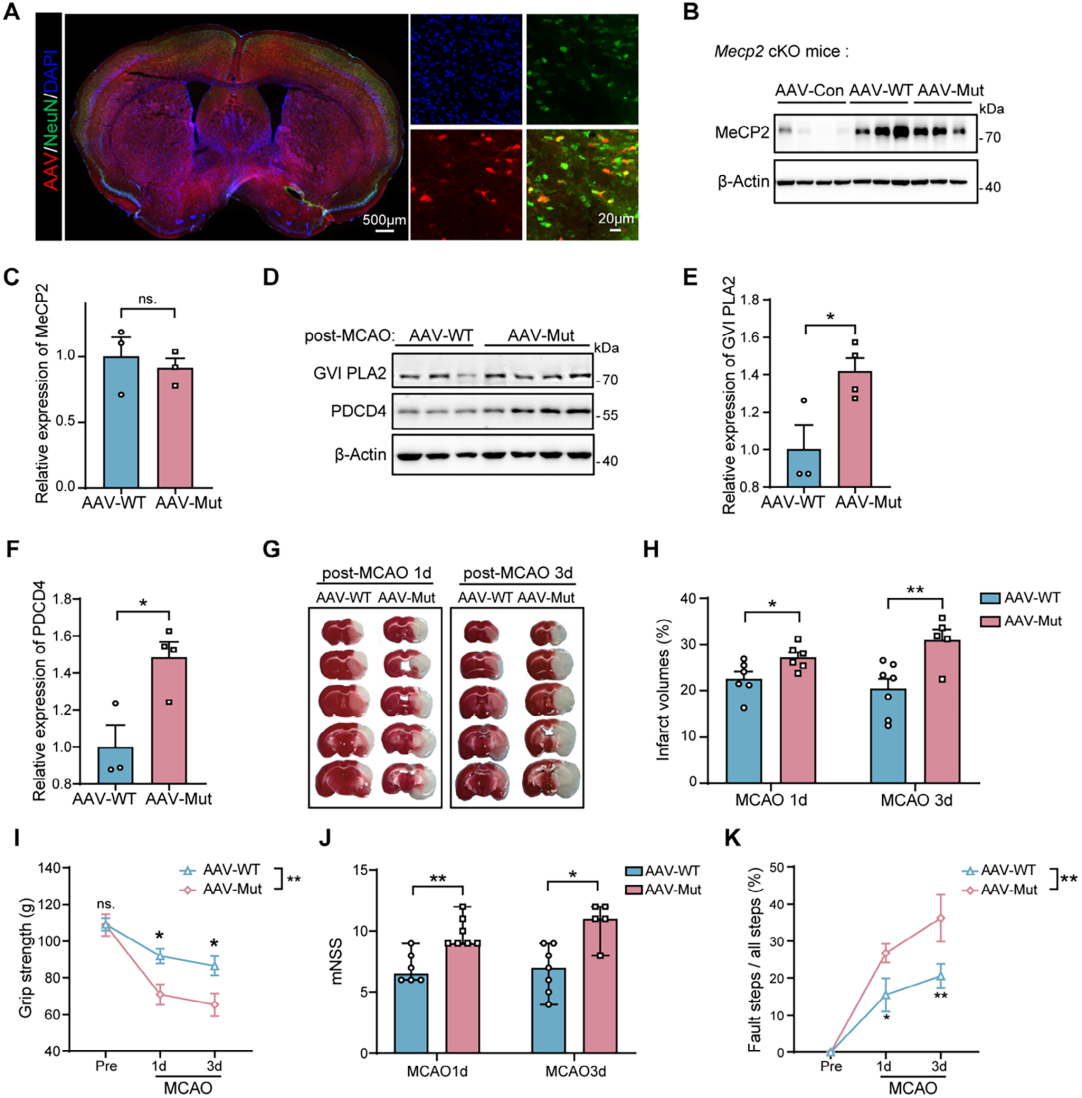
Neuronal MeCP2 lactylation at K210 and K249 improves stroke outcomes. A) Representative immunofluorescence images of brain sections co‐stained with DAPI (blue) and NeuN (green) at 21 d after adenoviruses (red) injection. B) Western blot analysis of MeCP2 expression in MeCP2 cKO mice injected with AAV‐Control (AAV‐Con), AAV‐wild type MeCP2 (AAV‐WT), or AAV‐K210R/K249R MeCP2 mutant (AAV‐Mut). C) Quantification of MeCP2 expression levels in the AAV‐WT and AAV‐Mut groups, showing no significant difference (*n* = 3 per group). D) Western blot analysis of GVI PLA2 and PDCD4 expression in the AAV‐WT and AAV‐Mut groups post‐MCAO. E) Quantification of GVI PLA2 and F) PDCD4 expression in AAV‐WT and AAV‐Mut groups post‐MCAO, showing increased levels in the AAV‐Mut group (*n* = 3–4 per group). G) Representative TTC staining of brain sections from AAV‐WT and AAV‐Mut mice at 1 and 3 d post‐MCAO. H) Quantification of infarct volumes in AAV‐WT and AAV‐Mut mice at 1 and 3 d post‐MCAO (*n* = 5–7 per group). I) Assessment of grip strength, J) mNSS scores, and K) fault step ratio in AAV‐WT and AAV‐Mut mice post‐MCAO (*n* = 5–7 per group). Modified Neurological Severity Scores (I) are shown as median ± ranges, and other values are presented as mean ± SEM. *p < 0.05, **p < 0.01; ns, not significant.

To further investigate the role of neuron‐specific MeCP2 lactylation in ischemic stroke, we employed *Mecp2* conditional knock out (cKO) mice lacking neuronal MeCP2, and then over‐expressed either wild‐type (WT) MeCP2 or the K210R/K249R double mutant via adeno‐associated virus (AAV) vectors (**Figure** **5**A,B). Western blot analysis confirmed comparable levels of MeCP2 expression in the cortex between the AAV‐WT and AAV‐Mutant groups, indicating effective transduction (Figure 5C). Post‐MCAO, we observed a significant increase in the expression of the pro‐apoptotic proteins GVI PLA2 and PDCD4 in the AAV‐Mutant group compared to the AAV‐WT group (Figure 5D–F). These findings confirm that the lactylation at K210 and K249 is crucial for suppressing the expression of these apoptotic markers in stroke. Furthermore, 2,3,5‐triphenyltetrazolium chloride (TTC) staining indicated a larger infarct volume in the AAV‐Mutant group at 1 and 3 d post‐MCAO (Figure 5G,H). Functional assessments revealed that grip strength, neurological severity scores, and fault step ratios were all significantly worse in the AAV‐Mutant group, indicating more severe functional impairments (Figure 5I–K). These findings indicate that lactylation at the K210 and K249 sites of MeCP2 in neurons functions as an intrinsic neuroprotective mechanism after ischemic stroke, with its absence leading to exacerbated ischemic damage and worsened functional outcomes.

To elucidate the regulatory mechanisms of MeCP2 lactylation, we conducted a series of knockdown experiments targeting various histone deacetylases (HDACs) and acetyltransferases in Neuro‐2a. Trichostatin A (TSA) is a broad‐spectrum inhibitor primarily targeting Class I and Class II histone deacetylases (HDACs). Treatment with TSA significantly increased MeCP2 lactylation at both K210 and K249 residues. Whereas, nicotinamide (NAM), the Class III HDACs inhibitor, did not affect MeCP2 lactylation (Figure S14A,B, Supporting Information). Furthermore, we targeted individual HDACs including HDAC1, HDAC2 and HDAC3, and found that the knockdown of HDAC3 resulted in a marked increase in MeCP2 lactylation at both sites, confirming its role as a MeCP2 delactylase (Figure S14C–J, Supporting Information). Conversely, the knockdown of p300 acetyltransferases significantly decreased MeCP2 lactylation, highlighting its critical role in facilitating this post‐translational modification. Additionally, knockdown of other previously reported acyltransferase including CBP, AARS1, GCNS, and PCAF did not induce significant changes in MeCP2 lactylation levels (Figure S14K–T, Supporting Information). Co‐IP assays showed interactions between MeCP2, p300, and HDAC3 (Figure S14U, Supporting Information). These findings reveal a complex regulatory mechanism involving HDAC3 and p300 in the modulation of MeCP2 lactylation.

### 2.12 HDAC3 and p300 Regulate MeCP2 Lactylation in Ischemic Stroke

**Figure 6:**
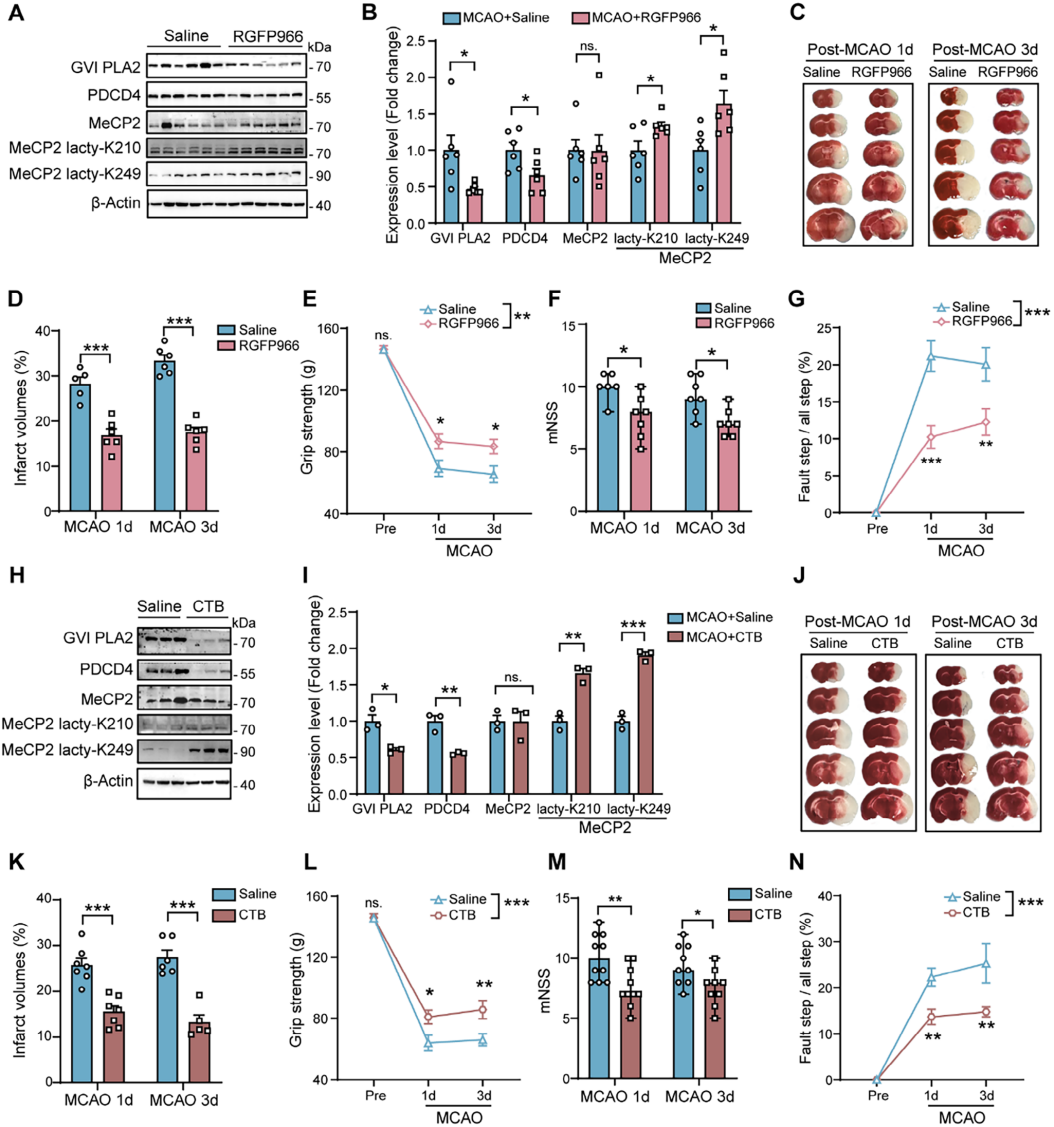
HDAC3 and p300 play roles in regulating of MeCP2 lactylation in ischemic stroke. A) Western blot analysis of MeCP2 lactylation at K210 and K249 in cortical samples from MCAO mice treated with saline or RGFP966. B) Quantification of GVI PLA2 and PDCD4 expression levels in the saline or RGFP966‐treated MCAO mice (*n* = 6 per group). C) Representative images of TTC staining showing RGFP966 reduced infarct volume of MCAO mice. D) Quantification of brain infarct volume in mice treated with saline and RGFP966 at 1 d and 3 d post‐MCAO (*n* = 5–7 per group). E) Evaluation of grip strength, F) mNSS scores, and G) fault step ratio in stroke mice treated with saline and RGFP966 (*n* = 6–7 per group). H) Western blot analysis showing the effect of CTB treatment on MeCP2 lactylation at K210 and K249 compared to saline. I) Quantification of GVI PLA2 and PDCD4 expression levels in mice treated with saline or CTB (*n* = 3 per group). J) Representative images of brain sections stained for infarction from mice treated with saline or CTB. K) Quantification of brain infarct volume in mice treated with saline or CTB (*n* = 5–7 per group). L) Grip strength test, M) mNSS scores, and N) fault step ratio for mice treated with saline or CTB (*n* = 9–10 per group). Modified Neurological Severity Scores (F, M) are shown as median ± ranges, and other values are presented as mean ± SEM. *p < 0.05, **p < 0.01, ***p < 0.001; ns, not significant.

To investigate the regulatory effects of HDAC3 and p300 on MeCP2 lactylation and their impact on ischemic stroke, we treated MCAO mice with RGFP966, a selective HDAC3 inhibitor, or CTB, an activator of the p300 enzyme, 12 h after reperfusion. Western blot analysis revealed that post‐MCAO treatment with RGFP966 significantly increased MeCP2 lactylation at K210 and K249. This increase was accompanied by a reduction in the expression of GVI PLA2 and PDCD4, compared to the saline‐treated group (**Figure** **6**A,B). RGFP966 treatment also resulted in a significant reduction in brain infarct volume at both 1 d and 3 d post‐MCAO (Figure 6C,D). Functional assessments indicated that RGFP966‐treated mice exhibited improved grip strength (Figure 6E), reduced modified Neurological Severity Scores (mNSS) (Figure 6F), and decreased fault step ratios (Figure 6G).

Consistently, stroke mice treated with CTB demonstrated a remarkable increase of MeCP2 lactylation at K210 and K249, along with lower expression level of GVI PLA2 and PDCD4 compared with the saline group (Figure 6H,I). CTB treatment also significantly reduced the brain infarct volume (Figure 6J,K) and enhanced neurological function, as demonstrated by better grip strength (Figure 6L), lower mNSS scores (Figure 6M), and fewer fault steps (Figure 6N) compared to saline‐treated controls. Collectively, these results indicate that both HDAC3 inhibition and p300 activation enhance MeCP2 lactylation, which in turn reduces ischemic brain damage and improves neurological outcomes. This highlights the potential therapeutic value of targeting HDAC3 and p300 to regulate MeCP2 lactylation in the treatment of ischemic stroke.

## 3 Discussion

In this study, we systematically explored the role of protein lactylation in acute ischemic stroke. Utilizing a mouse transient MCAO model, we observed significant increases in lactylation of non‐histone proteins within the ischemic penumbra. Notably, MeCP2 lactylation at K210 and K249 has emerged as a key modulator of apoptosis‐related gene expression, contributing to neuroprotection during the acute phase of stroke. Our findings provide critical insights into MeCP2 lactylation, suggesting a potential link between lactate metabolism and neuronal survival mechanisms in ischemic stroke.

Ischemic stroke is a major cause of morbidity and mortality worldwide. After the onset of cerebral ischemia, cerebral blood flow and oxygen consumption decrease, along with an increase in lactate production, suggesting a shift in cerebral metabolism from aerobic to anaerobic glycolysis.^[^ ^5^ ^]^ Lactate was once considered a waste product of anaerobic glycolysis and a marker of severe disease states and poor outcomes.^[^ ^4^, ^18^ ^]^ However, accumulating evidence suggests that lactate is a promising neuroprotective agent.^[^ ^19^, ^20^ ^]^ In experimental stroke models, the intracerebroventricular or intravenous administration of lactate after reperfusion effectively protected against ischemic brain damage and alleviated neurological deficits.^[^ ^7^, ^20^ ^]^ Notably, Zhang et al. described a novel epigenetic modification known as lactylation, which is derived from the cellular metabolite lactate.^[^ ^11^ ^]^ The cumulative data indicate that Kla is sensitive to lactate production via glycolysis.^[^ ^11^, ^12^, ^14^ ^]^ Consistently, we found that the Kla modification of proteins in the penumbra was induced in the acute phase of cerebral ischemia, paralleling changes in brain lactate. Blocking glycolysis with 2‐DG reduced both lactate and protein lactylation, exacerbating brain injury and neurological deficits in stroke mice. In contrast, lactate replacement with NALA enhanced lactylation, leading to smaller infarct sizes and improved neurological function. Notably, inhibiting lactate transport with 4‐CIN reduced lactylation without altering lactate levels, yet resulted in similar detrimental effects on stroke outcomes. Our findings suggest that protein lactylation may directly function as an intrinsic neuroprotective mechanism in response to cerebral ischemia, with effects partially independent of lactate itself.

Lactate is produced by glycolysis in various cell types and is enhanced under certain conditions, including hypoxia and ischemia.^[^ ^8^, ^21^ ^]^ In the CNS, active neurons preferentially consume lactate over glucose, with lactate utilization occurring shortly after activation begins.^[^ ^22^, ^23^ ^]^ Although the increased energy demand occurs mainly within neurons, astrocytes are highly efficient at using glycolysis to produce lactate after stimulation, which is then constitutively exported by astrocytes and shuttled into neurons as an alternative fuel source.^[^ ^9^, ^24^ ^]^ Astrocyte‐neuron lactate transport is required for fine‐tuning neuronal activity.^[^ ^9^, ^12^, ^24^ ^]^ Kla, derived from lactate molecules, reveals a mechanism by which cells connect cellular functions to lactate metabolism. In this study, we found that Kla modifications were widespread across different neural cells but predominantly located in the neuronal nucleus and processes. Stroke induced a marked increase in protein lactylation in neurons, with significant yet less pronounced lactylation in astrocytes. These distinct lactylation patterns between neurons and astrocytes likely reflect their different roles in the pathophysiology of ischemic stroke. Kla modification may also play a role in biochemical communication between neural cells following stroke.

Following ischemia, hypoperfusion of the brain tissue leads to a decrease in oxygen and glucose levels, which induces cell death over time.^[^ ^25^ ^]^ Glutamate excitotoxicity contributes to ischemic cascades. Excessive release of glutamate and activation of neuronal glutamate receptors are thought to be important triggers of ischemia‐induced neuronal damage.^[^ ^3^, ^26^ ^]^ In this study, bioinformatic analysis of lactylation proteomics revealed that protein lactylation may play a significant role in regulating neuronal cell death following ischemic stroke. In ischemic stroke, neuronal cell death is mediated by multiple mechanisms, each contributing uniquely to the overall pathology.^[^ ^27^, ^28^ ^]^ Our in vivo and in vitro studies demonstrated that protein lactylation plays a crucial role in regulating neuronal apoptosis following ischemic stroke while having minimal impact on other cell death pathways, such as pyroptosis and necroptosis. Flow cytometry and TUNEL staining, which detects DNA fragmentation as a marker of apoptosis, revealed that reducing lactylation through 2‐DG and 4‐CIN treatment significantly increased neuronal apoptosis, indicating that lower lactylation levels are directly associated with enhanced apoptotic cell death. In contrast, enhancing lactylation with NALA effectively reduced neuronal apoptosis and improved stroke outcomes. Importantly, despite the observed upregulation of necroptosis markers, such as p‐RIP3 post‐stroke, interventions modulating lactylation did not significantly influence these pathways. These findings highlight the specific role of lactylation in protecting neurons from apoptosis during the acute phase of ischemic injury.

Emerging evidence indicates that histone lactylation plays a crucial role in modulating gene expression, which contributes to the pathophysiology of CNS disorders. Hagihara et al. identified histone H1 lactylation as a key regulatory mechanism induced by neural excitation, which was associated with the expression of the neuronal activity marker c‐Fos and linked to altered social behavior.^[^ ^14^ ^]^ Histone lactylation has been implicated in the pathogenesis of Alzheimer's disease (AD). Histone H4K12 lactylation in microglia has been shown to alter the expression of glycolysis‐related genes, thereby contributing to the metabolic dysfunction and neuroinflammation observed in AD.^[^ ^15^ ^]^ Our study highlights the role of non‐histone protein lactylation in ischemic stroke. While histone lactylation remained largely unchanged, non‐histone proteins, particularly MeCP2, exhibited significant lactylation changes that correlated with neuroprotective outcomes, emphasizing the potential importance of non‐histone lactylation in neuronal survival during ischemic events (Table S5, Supporting Information).

MeCP2 plays a critical role in neuronal maturation throughout the CNS in mammals. Any disruption in the *Mecp2* gene, whether through loss of function or increased dosage, leads to impaired neuronal function and can result in a range of neuropsychiatric disorders.^[^ ^16^ ^]^ MeCP2 is a transcriptional regulator primarily localized in neuronal nuclei. It modulates gene expression epigenetically by binding to methylated CpG dinucleotides and core histones across the genome. Neuronal MeCP2 has been reported to function as a transcriptional silencer or activator of specific target genes.^[^ ^29^, ^30^ ^]^ In a previous study, MeCP2 was found to participate in neuronal plasticity during stroke via its downstream target genes.^[^ ^31^ ^]^ In our study, we uncovered a novel neuroprotective mechanism of MeCP2 during ischemic stroke. Notably, we observed that the nuclear distribution of MeCP2 in neurons shifts from a punctate to a more diffuse pattern following ischemic stroke. This alteration was partially reversed by inhibiting lactylation, highlighting the role of lactylation in modulating MeCP2 function in response to cerebral ischemia (Figure S5, Supporting Information). MeCP2 lactylation significantly enhanced the binding of MeCP2 to the promoters of apoptosis‐related genes, such as *Pdcd4* and *Pla2g6*, leading to their transcriptional repression. This, in turn, results in a reduction in neuronal apoptosis during the early phase of ischemic stroke. Inhibiting lactylation counteracted MeCP2 transcriptional repression, resulting in pro‐apoptotic gene upregulation and increased neuronal death. Furthermore, we demonstrated that neuronal MeCP2 lactylation at specific lysine residues (K210 and K249) was crucial for its transcriptional inhibition of *Pdcd4* and *Pla2g6* and neuroprotection in stroke. The human MeCP2 K210 site contains a single nucleotide polymorphism (rs61749730), potentially introducing a coding variant that alters lactylation. The impact of rs61749730 on cerebral ischemia pathophysiology, particularly in humans, remains to be further elucidated.

MeCP2 also play a key role on regulation of glial cell function, contributing to neuroinflammation, metabolic support, and neuronal survival.^[^ ^32^, ^33^ ^]^ MeCP2 deficiency in astrocytes disrupts metabolic homeostasis and neuroinflammatory balance, impairing neuronal support through aberrant secretion of soluble factors and dysregulated calcium signaling.^[^ ^34^, ^35^ ^]^ Transcriptomic analyses reveal that MeCP2 modulates astrocytic gene networks involved in proliferation and neuronal support, while its phosphorylation (pS292) in reactive astrocytes plays a role in VEGF‐mediated responses following stroke.^[^ ^36^, ^37^ ^]^ In microglia, the absence of MeCP2 induces a hyper‐inflammatory phenotype, reduces cell numbers, and perturbs homeostatic gene expression, exacerbating neuroinflammation and synaptic dysfunction.^[^ ^38^ ^]^ Additionally, MeCP2‐null microglia contribute to neural circuit damage by engulfing weakened synapses, whereas RIPK1 inhibition reduces oxidative stress, cytokine production, and glutamate release, mitigating neurodegenerative pathology.^[^ ^39^, ^40^ ^]^ MeCP2 also plays a critical role in regulating oligodendrocyte function, directly binding to the promoters of *Mbp*, *Plp*, and *Bdnf* to regulate myelin‐associated gene expression, and its deficiency in oligodendrocyte leads to impaired myelination.^[^ ^41^, ^42^ ^]^ Restoration of MeCP2 in oligodendrocyte lineage cells only partially rescues myelin‐related deficits, indicating additional non‐cell‐autonomous influences on oligodendrocyte function.^[^ ^43^ ^]^

HDAC3 and p300 are critical epigenetic regulators that are implicated in the pathophysiology of ischemic stroke.^[^ ^44^ ^]^ In this study, we demonstrated that enhancing MeCP2 lactylation via HDAC3 inhibition or p300 activation significantly mitigated ischemic brain injury and improved neurological outcomes. These interventions specifically increased MeCP2 lactylation at K210 and K249, which correlated with improvements in stroke outcomes in mice. Our findings suggest that targeting the enzymatic modulation of lactylation could offer a novel therapeutic approach for ischemic stroke, opening new avenues for exploring the epigenetic regulation of neuroprotection.

Overall, our findings reveal that MeCP2 lactylation at K210/K249 plays a neuroprotective role by repressing the transcription of apoptosis‐associated genes. We uncover a neuroprotective mechanism and offer potential novel strategies for improving stroke outcomes through epigenetic interventions.

## 4 Experimental Section

### Postmortem Human Brain Tissues

Detailed information of the postmortem human brain tissues is provided in Table S6 (Supporting Information). Informed consent documents were signed by donors or their legal representatives prior to donation. The peri‐infarct regions of postmortem human brain tissues were dissected into blocks and placed into fresh fixative (15% formalin in 0.1 m phosphate buffer, pH 7.4) for 24–48 h. The blocks were then transferred to 20% and 30% sucrose (0.1 m phosphate buffer with 0.1% Na‐azide) for 2–3 d sequentially. The dehydrated tissue blocks were stored in a −80 °C freezer and used for sectioning and immunostaining.^[^ ^45^ ^]^

### Animals

MeCP2 KO mice were generated using CRISPR/Cas9 technology purchased from GemPharmatech (Nanjing, China). Neuronal‐specific MeCP2 cKO mice were generated by breeding *Syn1*‐Cre mice with *MeCP2^fl/fl^* mice. Both strains were provided by Cyagen Bioscience Inc. (Guangzhou, China). Male C57BL/6 mice, ≈8 weeks old and weighing 25–30 g, were provided by the Animal Center of Nanjing Drum Tower Hospital. The mice were genotyped by PCR and sequencing using the primers listed in Table S7 (Supporting Information).

Mice were maintained under specific pathogen‐free conditions and used for experiments at 8–12 weeks of age. Female pregnant MeCP2^+/−^ mice were used for primary neuronal culture. MeCP2 cKO mice were intravenously injected with AAV, followed by 3 weeks of overexpression.

Mice were maintained under specific pathogen‐free conditions and used for experiments at 8–12 weeks of age. Female pregnant MeCP2^+/−^ mice were used for primary neuronal culture. MeCP2 cKO mice were intravenously injected with AAV, followed by 3 weeks of overexpression.

### Cell Cultures

All cells were cultured at 37 °C in a humidified 5% CO_2_ incubator. Primary cortical neurons were isolated from E16‐17 mouse embryo as previously described.^[^ ^46^ ^]^ Briefly, fetal cortices were dissected and treated with trypsin to prepare cell suspensions. The cells were then plated at a density of 5 × 10^5^ cells mL^−1^ on poly‐D‐lysine‐coated 12‐well or 96‐well plates. Cells were cultured in Neurobasal medium (Cat. 21103049, Gibco, United Kingdom) with B27 (Invitrogen, Carlsbad, USA) and 25 nm glutamine. The cells were used for experiments after 2 weeks of culture. Neuro‐2a cell line and HEK293T cell line were purchased from American Type Culture Collection (ATCC; USA). All cells were cultured in DMEM (Cat. 11965092, Gibco, USA) supplemented with 10% fetal bovine serum (FBS, Cat. 10099141C, Gibco, Australia) and 1% penicillin‐streptomycin (Cat. 15070063, Gibco, India).

### The Mouse Stroke Model

Mice were intraperitoneally anesthetized with sodium pentobarbital (1%) at a dose of 45 mg kg^−1^. MCAO model was developed as previously described.^[^ ^47^ ^]^ Briefly, a midline cervical incision was made under a dissecting microscope, and the right common and external carotid arteries (ECA) were isolated. A 6‐0 monofilament nylon suture with a heat‐rounded tip was introduced into a wedge‐shaped incision on the ECA and advanced to obstruct the origin of the middle cerebral artery. After 40 min of occlusion, reperfusion was initiated by withdrawal of the filament. The sham‐treated mice were subjected to the same procedure without MCAO. Mice were randomly assigned to the sham or MCAO groups. During the procedure, rectal temperature was maintained at 37 ± 0.5 °C.

### Behavior Test

The evaluation of neurological function included the mNSS test, grip strength test, and foot fault test. The mNSS test was used to assess motor functions, sensory functions, and reflexes 24 h after MCAO,^[^ ^48^ ^]^ as previously described. The investigators were blinded to the grouping of the experimental mice. Neurological function was graded on a scale of 0–18 points according to the NSS. One point was awarded for inability to perform tasks or for the absence of a tested reflex. The grip strength test assessed forelimb movement and muscle function by recording the maximum grip strength of an experimental mouse as it was held by the tail and pulled horizontally. The foot fault test measured the percentage of missed steps when the mice freely explored an elevated grid with 12 × 12 mm^2^ cells.^[^ ^49^ ^]^

### Measurement of Infarct Size

Brain tissues were harvested 24 h after reperfusion. Five 2 mm‐thick coronal sections from each brain were prepared for staining using TTC (Cat. 17779, Sigma, USA) in saline.^[^ ^50^ ^]^ Infarct size was calculated using image analysis software (Image Pro Plus 6.0; Media Cybernetics, Silver Spring, MD, USA). To rule out the effect of brain edema, the infarct volume was calculated as a percentage of the contralateral hemisphere.

### Drug Treatment

Forty minutes after MCAO treatment, the mice were intraperitoneally administered 2‐DG (2 g kg^−1^, Cat. HY‐13966, MCE, China), or 4‐CIN (120 mg kg^−1^; Cat. S8612, Selleck, USA). Lactate production was inhibited by disrupting glycolysis with 2‐DG or blocking extracellular lactate transport with 4‐CIN, using previously established effective doses.^[^ ^14^ ^]^ NALA (0.2 g kg^−1^, Cat. 71718, Sigma, China) was intraperitoneally injected into mice for 14 d consecutively before MCAO treatment.^[^ ^51^ ^]^ Additionally, 30 µmol kg^−1^ of the p300 activator CTB (Cat. E1107, Selleck, USA) or 10 mg kg^−1^ RGFP966 (Cat. S7229, Selleck, USA) was intraperitoneally injected 12 h post‐MCAO.^[^ ^51^, ^52^ ^]^ The control group received the same dosage of normal saline before and after surgery.

### Western Blot

To analyze protein lactylation levels, mice in each group were sacrificed, and cortical and striatal tissues were dissected from the ipsilateral hemispheres after 50 min of MCAO followed by 24 h of reperfusion. This procedure was previously described.^[^ ^48^ ^]^ Equal amounts of protein were separated by sodium dodecyl sulfate (SDS)‐polyacrylamide gel electrophoresis and blotted onto polyvinylidene fluoride (PVDF) membranes (Cat. 1620177, Bio‐Rad, USA).^[^ ^53^ ^]^ Membranes were probed with the primary antibodies at 4 °C overnight. The following primary antibodies were used: Anti‐L‐Lactyl Lysine Rabbit mAb (Cat. PTM‐1401, PTM BIO, China), Anti‐lacty‐MeCP2(Lys210) rabbit pAb (PTM BIO, China), Anti‐lacty‐MeCP2(Lys249) rabbit pAb (PTM BIO, China), Rabbit monoclonal anti‐MeCP2 (Cat. 3456, Cell Signaling Technology, USA), Rabbit monoclonal anti‐PDCD4 (Cat. 9535, Cell Signaling Technology, USA), Rabbit monoclonal anti‐HA‐Tag (Cat. 3724, Cell Signaling Technology, USA), Rabbit polyclonal anti‐Cleaved Caspase‐3 (Cat. 9661, Cell Signaling Technology, USA), Rabbit monoclonal anti‐HDAC1 (Cat. 34589, Cell Signaling Technology, USA), Mouse monoclonal anti‐HDAC2 (Cat. 5113, Cell Signaling Technology, USA), Mouse monoclonal anti‐HDAC3 (Cat. 3949, Cell Signaling Technology, USA), Rabbit monoclonal anti‐CHOP (Cat. 5554, Cell Signaling Technology, USA), Rabbit monoclonal anti‐Caspase‐1 (Cat. 24232, Cell Signaling Technology, USA), Rabbit monoclonal anti‐Phospho‐RIP3 (Thr231/Ser232) (Cat. 91702, Cell Signaling Technology, USA), Mouse monoclonal anti‐AlaRS (Cat. sc‐165990, Santa Cruz Biotechnology, USA), Mouse monoclonal anti‐p300 (Cat. sc‐48343, Santa Cruz Biotechnology, USA), Mouse monoclonal anti‐CBP (Cat. sc‐7300, Santa Cruz Biotechnology, USA), Mouse monoclonal anti‐group VI iPLA2 (Cat. sc‐376563, Santa Cruz Biotechnology, USA), Mouse monoclonal anti‐PCAF (Cat. sc‐13124, Santa Cruz Biotechnology, USA), Mouse monoclonal anti‐KAT2A/GCN5(Cat. 66575‐1‐lg, Proteintech, China), Rabbit polyclonal anti‐RIP3 (Cat. 17563‐1‐lg, Proteintech, China), Mouse monoclonal anti‐b‐actin (Cat. 66009‐1‐lg, Proteintech, China), Mouse GAPDH Monoclonal antibodies (Cat. 60004‐1‐Ig, Protsintech, China). Proteins were detected using horseradish peroxidase‐conjugated anti‐rabbit (Cat. ab6721, Abcam, USA) or anti‐mouse (Cat. ab6728, Abcam, USA) secondary antibodies and visualized using chemiluminescence reagents from the ECL kit (Bio‐Rad, 1705060, USA). All samples were normalized using β‐actin or GAPDH and semi‐quantitatively compared to the sham control.

### Immunostaining

Details of immunostaining protocols can be found in the previous publications.^[^ ^54^ ^]^ Primary neuronal cultures were fixed in 4% formaldehyde and incubated overnight with primary antibodies listed as following: Mouse monoclonal anti‐NeuN (Cat. ab104224, Abcam, USA), Chicken polyclonal anti‐MAP2 (Cat. ab5392, Abcam, USA), Rabbit polyclonal anti‐GFAP (Cat. ab7260, Abcam, USA), Goat polyclonal anti‐Iba1 (Cat. 5076, Abcam, USA), S100A10 Monoclonal antibody (Cat. 66227‐1‐Ig, Proteintech, China), Rabbit monoclonal anti‐MeCP2 (Cat. 3456, Cell Signaling Technology, USA), Anti‐L‐Lactyl Lysine Rabbit mAb (Cat. PTM‐1401, PTM BIO, China), Rabbit monoclonal anti‐PDCD4 (Cat. 9535, Cell Signaling Technology, USA), Mouse monoclonal anti‐HA‐tag (Cat. 2367, Cell Signaling Technology, USA). Antibody binding was visualized using fluorescent‐tagged secondary antibodies (1:500, Invitrogen, USA), and fluorescent images were captured using a confocal laser‐scanning microscope (Olympus, Japan).

### TUNEL Assay

Apoptosis in primary neurons was assessed using a TUNEL assay (Vazyme, Cat. A113‐01, China). Cells were fixed with 4% paraformaldehyde, permeabilized with 0.1% Triton X‐100, and labeled with the TUNEL reaction mixture according to the manufacturer's instructions. Fluorescent images were acquired using a confocal microscope (Olympus FV3000, Japan), and the percentage of TUNEL‐positive cells was quantified.

### Co‐IP Assay

Proteins from primary neurons were used for Co‐IP assays.^[^ ^50^ ^]^ The lysate was preincubated for 1 h at 4 °C with 25 µL of protein A/G‐agarose beads (Cat. IP10, Millipore, USA), then centrifuged to remove nonspecifically adhered proteins, and the resulting supernatant was collected for the subsequent IP experiment.^[^ ^55^ ^]^ The supernatant was rotated overnight at 4 °C with primary antibodies listed here: Rabbit monoclonal anti‐MeCP2 (Cat. 3456, Cell Signaling Technology, USA) and Mouse monoclonal anti‐HA‐tag (Cat. 2367, Cell Signaling Technology, USA). The following morning, protein A/G‐agarose beads were added. Immune complexes were isolated by centrifugation, washed, and boiled for 5 min in loading buffer. Proteins were analyzed by western blotting as previously described.

### EMSA

EMSA were conducted to analyze the protein‐DNA interactions. Recombinant proteins were expressed in 293F cells and purified using High Affinity Ni‐Charged Resin FF. Double‐stranded DNA probes were generated by annealing complementary single‐stranded oligonucleotides, labeled with biotin, and purified. Binding reactions were performed in 10 µL volumes containing 10 mm HEPES (pH 7.6), 50 mm KCl, 1 mm EDTA, 0.05% Triton X‐100, 5% glycerol, 1 mm dithiothreitol, and 50–100 nm of purified protein with a 2 nm labeled DNA probe. Reactions were incubated at room temperature for 30 min, then loaded onto a 6% native polyacrylamide gel in 0.5 × TBE buffer and electrophoresed at 4 °C for 2 h at 100 V. The DNA oligonucleotides were transferred onto nylon membranes at 200 mA for 90 min on ice. A Chemiluminescent Nucleic Acid Detection Module was used to detect binding reactions.

### OGD

Cortical cultures were subjected to OGD to simulate ischemia in vitro.^[^ ^50^ ^]^ Briefly, the cultures were switched from a normal culture medium to an oxygen‐depleted, glucose‐free medium. Cells were incubated in a hypoxia chamber previously flushed for 10 min with 5% CO_2_/95% N_2_ at 2 psi (1 psi = 6.89 kPA). Valves were closed, and chambers were incubated at 37 °C for 40 min. The cells were then returned to normal culture conditions for subsequent treatments.

### LC‐MS/MS

Samples were ground in liquid nitrogen and lysed in a buffer containing 8 m urea and 1% protease inhibitor. The lysates were sonicated, reduced with dithiothreitol, alkylated with iodoacetamide, and digested with trypsin. The resulting peptides were desalted, labeled with a TMT/iTRAQ kit, and fractionated using high‐pH reverse‐phase high‐performance liquid chromatography. The fractions were dried and reconstituted for analysis. Peptides were separated using an EASY‐nLC 1000 UPLC system and analyzed by MS/MS with a Q ExactiveTM Plus. MS data were processed using MaxQuant and searched against the human Uniprot database. The key parameters included trypsin/P specificity, up to four missed cleavages, precursor mass tolerances of 20 ppm (first search) and 5 ppm (main search), a fragment mass tolerance of 0.02 Da, and fixed/variable modifications of carbamidomethylation (Cys), acetylation (N‐terminus), and oxidation (Met). The false discovery rate was set to <1%.

### CUT&Tag

Peri‐infarct region samples were collected to detect the DNA binding sites of lactyl‐MeCP2 using CUT&Tag. The CUT&Tag assay was performed as described in the previous publications.^[^ ^56^ ^]^ Briefly, each sample was incubated with 10 µL of concanavalin A‐coated magnetic beads (Bangs Laboratories, USA) at room temperature for 15 min. Subsequently, the bead‐bound cells were resuspended in Dig‐wash Buffer (20 mm HEPES pH 7.5; 150 mm NaCl; 0.5 mm Spermidine; 1× Protease inhibitor cocktail; 0.05% Digitonin) with a 1:50 dilution of anti‐MeCP2 (Cat. 3456, Cell Signaling Technology, USA) antibodies or normal rabbit IgG (Cat. 2729, Cell Signaling Technology, USA) and incubated on a rotating platform overnight at 4 °C. After removing the primary antibody, the appropriate secondary antibody was added. The samples were then incubated with pA‐Tn5 at room temperature for 1 h. The samples were resuspended in tagmentation buffer and incubated at 37 °C for 1 h, after which the DNA was extracted and purified. Sequencing was performed on an Illumina (USA) NovaSeq 6000 using 150 bp paired‐end sequencing, according to the manufacturer's instructions. Analysis was performed using the deepTools tool (Shanghai Jiayin Biotechnology, China).

### Tissue Extraction and Flow Cytometry

Mice were anesthetized and transcardially perfused with ice‐cold phosphate‐buffered saline (PBS) to remove red blood cells. Cortical tissues from the ischemic hemisphere were collected, minced, and digested with 2000 µL accutase (Cat. A1110501, Gibco, USA) at 37 °C for 10 min. After digestion, the suspension was filtered through a 70 µm filter, and cells were pelleted by centrifugation at 300 *g* for 10 min at 4 °C. The pellet was resuspended in PBS, mixed with a debris removal solution, and a gradient was formed by adding PBS along the tube wall. After centrifugation at 3000 *g* for 10 min, the bottom layer was collected and centrifuged again at 2000 *g*, and the final pellet was resuspended in PBS for further analysis.^[^ ^57^ ^]^

For flow cytometry, the cells were stained with anti‐Kla (Alexa Fluor 647) and cell type markers: anti‐GLAST (PE) for astrocytes (Cat. 130‐118‐483, Miltenyi Biotec, Germany), anti‐CD45 (PE‐Cy7) (Cat. 561868, BD Biosciences, USA) and anti‐CD11b (FITC) (Cat. 561688, BD Biosciences, USA) for microglia, and anti‐NCAM1 (BV421) (Cat. 742657, BD Biosciences, USA) for neurons. Dead cells were identified using the Fixable Viability Stain 780 (Cat. 565388, BD Biosciences, USA). Staining was performed in the dark at 4 °C for 30 min, and the cells were analyzed using a FACSAria II (BD Biosciences, USA).^[^ ^58^ ^]^

### ChIP‐qPCR

ChIP‐qPCR was performed using the Hyperactive pG‐MNase CUT&RUN Assay Kit (Cat. HD101‐01, Vazyme, China) according to the manufacturer's instructions. Briefly, the cortical tissues were crosslinked with 1% formaldehyde for 10 min and quenched with glycine (125 mm final concentration). The tissues were lysed in lysis buffer (1% SDS, 5 mm EDTA, and 50 mm Tris‐HCl, pH 8.1) with protease inhibitors and sonicated to produce chromatin fragments. The chromatin was diluted and incubated overnight at 4 °C with either anti‐MeCP2 or control IgG antibodies to allow for specific binding. Immunocomplexes were pulled down using protein A/G Sepharose beads, followed by sequential washing with TSE I, II, and III buffers. The chromatin was eluted with TE buffer (1% SDS, 0.1 m NaHCO_3_), de‐crosslinked, and purified. qPCR was then performed using SYBR Green PCR Master Mix (Cat. 4309155, Applied Biosystems, USA).

### Measurement of Lactate Levels

Lactate levels were measured using an L‐Lactate Assay Kit (Cat. ab65331, Abcam, UK) according to the manufacturer's instructions. Briefly, the right hemispheres from sham and MCAO mice were harvested, washed in cold PBS, and homogenized in a Lactate Assay Buffer on ice using a Dounce homogenizer. The homogenates were centrifuged at 4 °C for 5 min at top speed to remove insoluble material. The supernatant was collected, and 50 µL of each sample was added to a 96‐well plate. The reaction mix consisting of lactate assay buffer, lactate substrate mix, and lactate enzyme mix was prepared and added to the wells. After incubation at room temperature for 30 min, absorbance was measured at 450 nm using a microplate reader (Tecan, Spark 10 m). Lactate concentrations were determined by comparing absorbance readings to a standard curve generated from known lactate concentrations.

### Intravenous Administration of AAV in Mice

AAV was administered to the mice via intravenous injection. Mice were anesthetized with isoflurane. AAV particles (≈1 × 10^11^ viral genomes per mouse) were diluted in sterile PBS to a final injection volume of 200 µL. Diluted AAV was slowly injected into the tail vein using a 30‐G insulin syringe. After injection, the mice were monitored until they fully recovered from the anesthesia and observed daily for any signs of discomfort or complications. The mice were allowed to recover for 3 weeks to ensure sufficient viral expression.

### Nuclear and Cytoplasmic Protein Extraction

Cell lysates were isolated using cytoplasmic lysis buffer (20 mm HEPES, 100 mm KCl, 10 mm EDTA, 10% glycerol, 0.1% NP‐40, and protease inhibitors) and centrifuged at 1700 *g* for 5 min. The supernatants were collected as cytoplasmic extracts for subsequent western blot analysis. The pellets were washed four times with the cytoplasmic lysis buffer and then lysed with the nuclear lysis buffer (20 mm HEPES, 100 mm KCl, 420 mm NaCl, 10 mm EDTA, 20% glycerol, 1% NP‐40, and a protease inhibitor). After centrifugation at 14 000 *g* for 10 min, the supernatants were collected as nuclear extracts for western blot analysis.

### PLA

To assess protein‐protein interactions and lactylation modifications, PLA was performed according to the manufacturer's instructions (Cat. DUO92202, Sigma, Germany). Mouse brain sections were fixed with 4% paraformaldehyde, permeabilized with 0.1% Triton X‐100, and blocked using Duolink Blocking Solution for 60 min at 37 °C. Sections were then incubated overnight at 4 °C with primary antibodies against MeCP2 (Cat. M4773, Sigma, USA) and lactylation (Cat. PTM‐1401, PTM BIO, China). The following day, PLA probes (PLUS and MINUS) specific to the species of the primary antibodies were applied. After washing, ligation and amplification were performed using Duolink Detection Reagents. Fluorescent PLA signals, indicating protein interactions or post‐translational modifications, were visualized using a fluorescence microscope. Images were captured, and the PLA signal was quantified using ImageJ software (HIN), reporting the number of PLA dots per field of view.

### CCK8

Primary neurons were cultured in 96‐well plates and treated with different concentrations. After 2‐DG or NALA treatment, cells were incubated with a new medium containing CCK8 working solution (Cat. KGA317, KeyGEN BioTECH, China) at 37 °C in dark for 4 h. The absorption at 450 nm was measured to assess cell viability in each group.

### Real‐Time Quantitative Polymerase Chain Reaction (RT‐qPCR)

Total RNA was isolated from the peri‐infarcted cortex using TRIzol reagent (Cat. 15596026CN, Invitrogen, USA) and reverse transcribed using the Evo M‐MLV RT Mix Kit (Cat. AG11728, Accurate Biotechnology, China) according to the manufacturer's instructions. RT‐qPCR was performed using a SYBR Green kit (Cat. 4309155, Applied Biosystems, UK). The cycle time values were normalized relative to β‐actin. Detailed primer sequences are listed in Table S7 (Supporting Information).

### Dot Blot

The PVDF membrane, cut into the appropriate size, was activated in methanol for 1 min, then washed and dried at room temperature until the water film disappeared. Different amounts of the modified or control peptides were dropped onto the PVDF membrane. To assess the specificity of the antibody purified from the rabbit serum, the membrane was incubated overnight at 4 °C. After binding with the secondary antibody, the membrane was visualized using a confocal laser‐scanning microscope (Olympus, Japan).

### PRM Analysis

Following proteomic screening, PRM was used for the targeted quantification of lactylated peptides. Protein samples were extracted from the brain tissues, digested with trypsin, and desalted. The peptides were then separated using a reversed‐phase liquid chromatography system (EASY‐nLC 1000 UPLC) with a gradient from 6% to 80% acetonitrile. MS analysis was performed using a Q Exactive Plus mass spectrometer (Thermo Fisher Scientific) in PRM mode, with the electrospray voltage set to 2.0 kV. The mass‐to‐charge ratio (*m*/*z*) scan range was 350–1000 for full MS, and the peptide fragments were analyzed at a resolution of 17 500. PRM data were processed using Skyline (v3.6), and lactylated peptides, such as MeCP2 K210, were quantified based on the peak area. The relative peptide abundance was normalized to internal standards. All experiments were conducted in triplicate.

### Bioinformatics Analysis

GO annotation was conducted using the UniProt‐GOA database to classify proteins into BP, CC, and MF categories. Protein domain annotations were obtained using InterProScan, which utilizes data from the InterPro database. Pathway analysis was performed using the KEGG database, with KAAS for annotation and KEGG Mapper for pathway mapping. Subcellular localization was predicted using WoLF PSORT for eukaryotic sequences and CELLO for prokaryotic sequences. Functional enrichment analysis for GO terms, KEGG pathways, and protein domains was performed using Fisher's exact test, with significance set at a corrected *p* < 0.05. Enrichment‐based clustering was visualized via heat maps using the heatmap.2 function in the R package gplots. Protein‐protein interaction networks were constructed using the STRING database, with interactions having a confidence score of 0.7 or higher. These networks were then visualized using the networkD3 R package.

### Plasmids and Transfection

The full‐length MeCP2 sequence and the indicated mutants were obtained from mouse brain tissue cDNA. The promoter region sequences of *Pdcd4* and *Pla2g6* were synthesized by General Biology and then cloned into the pGL‐SV40 luciferase reporter vector. shRNAs were purchased from Sigma‐Aldrich (Table S7, Supporting Information). All plasmids were confirmed by sequencing. Cells were transfected using either Lipofectamine 3000 or Polyethylenimine (MW25000). For lentivirus packaging, viral supernatants were collected after co‐transfection with the lentiviral vectors, psPAX2 and pMD2.G.

### CRISPR/Cas9‐Mediated MeCP2 KO in HEK293T Cells

Single‐guide sequences (AGAAGGGTCAGGCTCCGCCC) of MeCP2 were cloned into the lentiCRISPRv2 vector. HEK293T cells were selected with puromycin (1 μ mL^−1^) 5 d after transfection. Stable cell clones were isolated and detected by IB analysis.

### Luciferase Reporter Assay

HEK293T cells were transfected with luciferase reporter constructs containing *Pdcd4* or *Pla2g6* promoters. After 24 h, luciferase activity was measured using a Dual‐Luciferase Reporter Assay System (Cat. E1980, Promega, USA) according to the manufacturer's instructions. Firefly luciferase activity was normalized to Rluc activity, and the results were expressed as relative luciferase activity.

### Preparation of Lactylation Antibody

Three peptides (two modified and one non‐modified) were designed based on the target protein sequence and identified by MS. After immunizing New Zealand white rabbits, serum samples (titer of 1:10 000, OD450 > 1) were analyzed using enzyme‐linked immunosorbent assay, Dot blot, and western blotting. The optimal serum was selected for Protein A affinity chromatography and immunoaffinity column purification to screen for a modified MeCP2 antibody.

### Statistical Analysis

Statistical analyses were performed using the GraphPad Prism software (version 9.0.0; GraphPad, La Jolla, CA, USA). A two‐tailed unpaired Student's *t*‐test was used to compare the two groups. One‐way or two‐way analysis of variance was performed to determine the differences among three or more groups, followed by Tukey's multiple comparison test for equal variances or Dunnett's multiple comparison test when the variances were unequal. For normally distributed data, values were presented as mean ± SEM. For data that were not normally distributed, values were expressed as median ± ranges and analyzed by the Mann–Whitney test. Statistical significance was set at *p*‐values < 0.05. Details regarding the number of individual mice and experiments (*n*) are provided in the figure legends.

## Conflict of Interest

The authors declare no conflict of interest.

## Author Contributions

Y.X. and Y.C. (Yanting Chen) conceived and directed the study. M.S., Y.Z., and R.M. designed and performed most of the experiments. M.S., Y.C. (Yan Chen), and P.L. were responsible for data acquisition and analysis. L.Y., S.X. (Siyi Xu), and S.X. (Shengnan Xia) conducted the mouse model experiments and genotyping. S.S. and L.H. performed the bioinformatic analysis of LC/MS and CUT&Tag data. Y.Y. was responsible for the primary neuron cultures, and J.J. performed immunofluorescence staining on human brain samples. Y.C. (Yanting Chen) and M.S. prepared the manuscript and figures. M.S., Y.Z., and R.M. contributed equally to this work.

## Ethics Approval Statement

Frozen postmortem human brains from patients who died of cerebral ischemic stroke were obtained from the Chinese Brain Bank Center (approval number: 2021‐scuec‐034). The animal study was conducted in accordance with the National Regulations of Experimental Animal Administration, and all the experimental protocols were approved by the Animal Care and Use Committee of the Model Animal Research Center, Nanjing Drum Tower Hospital, Affiliated Hospital of Medical School, Nanjing University (approval number: 2023AE01068).

## Supporting information



Supporting Information

## Data Availability

The data that support the findings of this study are available in the supplementary material of this article.

## References

[1] R. Mao , N. Zong , Y. Hu , Y. Chen , Y. Xu , Neurosci. Bull. 2022, 38, 1229. 35513682 10.1007/s12264-022-00859-0 PMC9554175

[2] W. A. Pulsinelli , D. E. Levy , T. E. Duffy , Ann. Neurol. 1982, 11, 499. 7103426 10.1002/ana.410110510

[3] D. J. Rossi , J. D. Brady , C. Mohr , Nat. Neurosci. 2007, 10, 1377. 17965658 10.1038/nn2004 PMC8906499

[4] P. J. Magistretti , I. Allaman , Nat. Rev. Neurosci. 2018, 19, 235. 29515192 10.1038/nrn.2018.19

[5] J. S. Meyer , T. Sawada , A. Kitamura , M. Toyoda , Circulation 1968, 37, 1036. 5653046 10.1161/01.cir.37.6.1036

[6] R. Leigh , L. Knutsson , J. Zhou , P. C. van Zijl , J. Cereb. Blood Flow Metab. 2018, 38, 1500. 28345479 10.1177/0271678X17700913 PMC6125975

[7] C. Berthet , X. Castillo , P. J. Magistretti , L. Hirt , Cerebrovasc. Dis. 2012, 34, 329. 23154656 10.1159/000343657

[8] D. C. Lee , H. A. Sohn , Z.‐Y. Park , S. Oh , Y. K. Kang , K.‐M. Lee , M. Kang , Y. J. Jang , S.‐J. Yang , Y. K. Hong , H. Noh , J.‐A. Kim , D. J. Kim , K.‐H. Bae , D. M. Kim , S. J. Chung , H. S. Yoo , D.‐Y. Yu , K. C. Park , Y. I. Yeom , Cell 2015, 161, 595. 25892225 10.1016/j.cell.2015.03.011

[9] A. Suzuki , S. A. Stern , O. Bozdagi , G. W. Huntley , R. H. Walker , P. J. Magistretti , C. M. Alberini , Cell 2011, 144, 810. 21376239 10.1016/j.cell.2011.02.018 PMC3073831

[10] W. G. Kaelin , S. L. McKnight , Cell 2013, 153, 56. 23540690 10.1016/j.cell.2013.03.004 PMC3775362

[11] D. Zhang , Z. Tang , H. Huang , G. Zhou , C. Cui , Y. Weng , W. Liu , S. Kim , S. Lee , M. Perez‐Neut , J. Ding , D. Czyz , R. Hu , Z. Ye , M. He , Y. G. Zheng , H. A. Shuman , L. Dai , B. Ren , R. G. Roeder , L. Becker , Y. Zhao , Nature 2019, 574, 575. 31645732 10.1038/s41586-019-1678-1 PMC6818755

[12] Y. A. Vladimirov , E. V. Proskurnina , Biochemistry (Moscow) 2009, 74, 1545. 20210708 10.1134/s0006297909130082

[13] R. A. Irizarry‐Caro , M. M. McDaniel , G. R. Overcast , V. G. Jain , T. D. Troutman , C. Pasare , Proc. Natl. Acad. Sci. U. S. A. 2020, 117, 30628. 33199625 10.1073/pnas.2009778117 PMC7720107

[14] H. Hagihara , H. Shoji , H. Otabi , A. Toyoda , K. Katoh , M. Namihira , T. Miyakawa , Cell Rep. 2021, 37, 109820. 34644564 10.1016/j.celrep.2021.109820

[15] R.‐Y. Pan , L. He , J. Zhang , X. Liu , Y. Liao , J. Gao , Y. Liao , Y. Yan , Q. Li , X. Zhou , J. Cheng , Q. Xing , F. Guan , J. Zhang , L. Sun , Z. Yuan , Cell Metab. 2022, 34, 634. 35303422 10.1016/j.cmet.2022.02.013

[16] A. J. Sandweiss , V. L. Brandt , H. Y. Zoghbi , Lancet Neurol. 2020, 19, 689.32702338

[17] S. R. D'Mello , J. Neurochem. 2021, 159, 29.33638179

[18] G. A. Dienel , J. Cereb. Blood Flow Metab. 2012, 32, 1107. 22186669 10.1038/jcbfm.2011.175 PMC3390802

[19] H. Roumes , U. Dumont , S. Sanchez , L. Mazuel , J. Blanc , G. Raffard , J.‐F. Chateil , L. Pellerin , A.‐K. Bouzier‐Sore , J. Cereb. Blood Flow Metab. 2021, 41, 342. 32208801 10.1177/0271678X20908355 PMC7812521

[20] C. Berthet , H. Lei , J. Thevenet , R. Gruetter , P. J. Magistretti , L. Hirt , J. Cereb. Blood Flow Metab. 2009, 29, 1780. 19675565 10.1038/jcbfm.2009.97

[21] J. Zhang , J. Muri , G. Fitzgerald , T. Gorski , R. Gianni‐Barrera , E. Masschelein , G. D'Hulst , P. Gilardoni , G. Turiel , Z. Fan , T. Wang , M. Planque , P. Carmeliet , L. Pellerin , C. Wolfrum , S.‐M. Fendt , A. Banfi , C. Stockmann , I. Soro‐Arnáiz , M. Kopf , K. De Bock , Cell Metab. 2020, 31, 1136. 32492393 10.1016/j.cmet.2020.05.004 PMC7267778

[22] I. A. Simpson , A. Carruthers , S. J. Vannucci , J. Cereb. Blood Flow Metab. 2007, 27, 1766. 17579656 10.1038/sj.jcbfm.9600521 PMC2094104

[23] A. Aubert , R. Costalat , P. J. Magistretti , L. Pellerin , Proc. Natl. Acad. Sci. U. S. A. 2005, 102, 16448. 16260743 10.1073/pnas.0505427102 PMC1297516

[24] D. Jimenez‐Blasco , A. Busquets‐Garcia , E. Hebert‐Chatelain , R. Serrat , C. Vicente‐Gutierrez , C. Ioannidou , P. Gómez‐Sotres , I. Lopez‐Fabuel , M. Resch‐Beusher , E. Resel , D. Arnouil , D. Saraswat , M. Varilh , A. Cannich , F. Julio‐Kalajzic , I. Bonilla‐Del Río , A. Almeida , N. Puente , S. Achicallende , M.‐L. Lopez‐Rodriguez , C. Jollé , N. Déglon , L. Pellerin , C. Josephine , G. Bonvento , A. Panatier , B. Lutz , P.‐V. Piazza , M. Guzmán , L. Bellocchio , et al., Nature 2020, 583, 603. 32641832 10.1038/s41586-020-2470-y

[25] A. Datta , D. Sarmah , L. Mounica , H. Kaur , R. Kesharwani , G. Verma , P. Veeresh , V. Kotian , K. Kalia , A. Borah , X. Wang , K. R. Dave , D. R. Yavagal , P. Bhattacharya , Transl. Stroke Res. 2020, 11, 1185. 32219729 10.1007/s12975-020-00806-z

[26] T. W. Lai , S. Zhang , Y. T. Wang , Prog. Neurobiol. 2014, 115, 157. 24361499 10.1016/j.pneurobio.2013.11.006

[27] Q.‐Z. Tuo , S.‐T. Zhang , P. Lei , Med. Res. Rev. 2022, 42, 259. 33957000 10.1002/med.21817

[28] M. G. Naito , D. Xu , P. Amin , J. Lee , H. Wang , W. Li , M. Kelliher , M. Pasparakis , J. Yuan , Proc. Natl. Acad. Sci. U. S. A. 2020, 117, 4959. 32071228 10.1073/pnas.1916427117 PMC7060720

[29] M. Chahrour , S. Y. Jung , C. Shaw , X. Zhou , S. T. C. Wong , J. Qin , H. Y. Zoghbi , Science 2008, 320, 1224. 18511691 10.1126/science.1153252 PMC2443785

[30] M. J. Lyst , A. Bird , Nat. Rev. Genet. 2015, 16, 261. 25732612 10.1038/nrg3897

[31] L. Yang , B. Han , Z. Zhang , S. Wang , Y. Bai , Y. Zhang , Y. Tang , L. Du , L. Xu , F. Wu , L. Zuo , X. Chen , Y. Lin , K. Liu , Q. Ye , B. Chen , B. Li , T. Tang , Y. Wang , L. Shen , G. Wang , M. Ju , M. Yuan , W. Jiang , J. H. Zhang , G. Hu , J. Wang , H. Yao , Circulation 2020, 142, 556. 32441115 10.1161/CIRCULATIONAHA.120.045765

[32] X.‐R. Jin , X.‐S. Chen , L. Xiao , Front. Mol. Neurosci. 2017, 10, 316. 29046627 10.3389/fnmol.2017.00316 PMC5632713

[33] U. Kahanovitch , K. C. Patterson , R. Hernandez , M. L. Olsen , Int. J. Mol. Sci. 2019, 20, 3813. 31387202 10.3390/ijms20153813 PMC6696322

[34] N. Ballas , D. T. Lioy , C. Grunseich , G. Mandel , Nat. Neurosci. 2009, 12, 311. 19234456 10.1038/nn.2275 PMC3134296

[35] Q. Dong , Q. Liu , R. Li , A. Wang , Q. Bu , K. H. Wang , Q. Chang , Elife 2018, 7, 33417. 10.7554/eLife.33417 PMC5902163 29595472

[36] D. H. Yasui , H. Xu , K. W. Dunaway , J. M. Lasalle , L.‐W. Jin , I. Maezawa , Mol. Autism 2013, 4, 3. 23351786 10.1186/2040-2392-4-3 PMC3561260

[37] F. Liu , J.‐J. Ni , J.‐J. Huang , Z.‐W. Kou , F.‐Y. Sun , Brain Res. 2015, 1599, 32. 25511996 10.1016/j.brainres.2014.12.014

[38] D. P. Schafer , B. Stevens , Immunity 2015, 42, 600. 25902477 10.1016/j.immuni.2015.04.002

[39] D. P. Schafer , C. T. Heller , G. Gunner , M. Heller , C. Gordon , T. Hammond , Y. Wolf , S. Jung , B. Stevens , Elife 2016, 5, 15224. 10.7554/eLife.15224 PMC4961457 27458802

[40] Z. Cao , X. Min , X. Xie , M. Huang , Y. Liu , W. Sun , G. Xu , M. He , K. He , Y. Li , J. Yuan , Proc. Natl. Acad. Sci. U. S. A. 2024, 121, 2320383121. 10.1073/pnas.2320383121 PMC10861890 38289948

[41] K. Sharma , J. Singh , P. P. Pillai , J. Mol. Neurosci. 2018, 65, 343. 29992497 10.1007/s12031-018-1112-4

[42] Z. S. Parikh , A. Tripathi , P. P. Pillai , J. Mol. Neurosci. 2017, 62, 309. 28616777 10.1007/s12031-017-0939-4

[43] M. V. C. Nguyen , C. A. Felice , F. Du , M. V. Covey , J. K. Robinson , G. Mandel , N. Ballas , J. Neurosci. 2013, 33, 18764. 24285883 10.1523/JNEUROSCI.2657-13.2013 PMC3841446

[44] Y. Liao , J. Cheng , X. Kong , S. Li , X. Li , M. Zhang , H. Zhang , T. Yang , Y. Dong , J. Li , Y. Xu , Z. Yuan , Theranostics 2020, 10, 9644. 32863951 10.7150/thno.47651 PMC7449914

[45] H. J. Waldvogel , M. A. Curtis , K. Baer , M. I. Rees , R. L. M. Faull , Nat. Protoc. 2006, 1, 2719. 17406528 10.1038/nprot.2006.354

[46] L. Ye , S. Shu , J. Jia , M. Sun , S. Xu , X. Bao , H. Bian , Y. Liu , M. Zhang , X. Zhu , F. Bai , Y. Xu , Aging Cell 2023, 22, 13860. 10.1111/acel.13860 PMC10352562 37177836

[47] H. Meng , H. Zhao , X. Cao , J. Hao , H. Zhang , Y. Liu , M.‐S. Zhu , L. Fan , L. Weng , L. Qian , X. Wang , Y. Xu , Proc. Natl. Acad. Sci. U. S. A. 2019, 116, 5558. 30819895 10.1073/pnas.1814394116 PMC6431175

[48] J. Chen , X. Li , S. Xu , M. Zhang , Z. Wu , X. Zhang , Y. Xu , Y. Chen , Front. Cell. Neurosci. 2020, 14, 77. 32317937 10.3389/fncel.2020.00077 PMC7146057

[49] P. Liu , Y. Chen , Z. Zhang , Z. Yuan , J.‐G. Sun , S. Xia , X. Cao , J. Chen , C.‐J. Zhang , Y. Chen , H. Zhan , Y. Jin , X. Bao , Y. Gu , M. Zhang , Y. Xu , PLoS Biol. 2023, 21, 3002199. 10.1371/journal.pbio.3002199 PMC10365314 37486903

[50] Y. Chen , Z. Wu , X. Zhu , M. Zhang , X. Zang , X. Li , Y. Xu , Exp. Neurol. 2019, 316, 52. 30981804 10.1016/j.expneurol.2019.04.005

[51] N. Zhang , Y. Zhang , J. Xu , P. Wang , B. Wu , S. Lu , X. Lu , S. You , X. Huang , M. Li , Y. Zou , M. Liu , Y. Zhao , G. Sun , W. Wang , D. Geng , J. Liu , L. Cao , Y. Sun , Cell Res. 2023, 33, 679. 37443257 10.1038/s41422-023-00844-w PMC10474270

[52] M.‐J. Zhang , Q.‐C. Zhao , M.‐X. Xia , J. Chen , Y.‐T. Chen , X. Cao , Y. Liu , Z.‐Q. Yuan , X.‐Y. Wang , Y. Xu , FASEB J. 2020, 34, 648. 31914678 10.1096/fj.201900394RRR

[53] L. Ye , M. Hu , R. Mao , Y. Tan , M. Sun , J. Jia , S. Xu , Y. Liu , X. Zhu , Y. Xu , F. Bai , S. Shu , CNS Neurosci. Ther. 2024, 30, 14555. 10.1111/cns.14555 PMC11163192 38105588

[54] S. Shu , S.‐Y. Xu , L. Ye , Y. Liu , X. Cao , J.‐Q. Jia , H.‐J. Bian , Y. Liu , X.‐L. Zhu , Y. Xu , Neuropsychopharmacology 2023, 48, 391. 36229597 10.1038/s41386-022-01435-w PMC9750960

[55] S.‐Y. Xu , J.‐Q. Jia , M. Sun , X.‐Y. Bao , S.‐N. Xia , S. Shu , P.‐Y. Liu , S.‐L. Ji , L. Ye , X. Cao , Y. Xu , iScience. 2023, 26, 107268. 37496671 10.1016/j.isci.2023.107268 PMC10366503

[56] J. Chen , Z. Zhang , Y. Liu , L. Huang , Y. Liu , D. Yang , X. Bao , P. Liu , Y. Ge , Q. Li , X. Shu , L. Xu , Y. S. Shi , X. Zhu , Y. Xu , Alzheimers Dement. 2024, 20, 3504. 38605605 10.1002/alz.13819 PMC11095431

[57] X. Dong , Z. Zhang , X. Shu , Z. Zhuang , P. Liu , R. Liu , S. Xia , X. Bao , Y. Xu , Y. Chen , Neurosci. Bull. 2024, 40, 483. 37979054 10.1007/s12264-023-01147-1 PMC11003935

[58] J. Jia , L. Zheng , L. Ye , J. Chen , S. Shu , S. Xu , X. Bao , S. Xia , R. Liu , Y. Xu , M. Zhang , Cell Death Dis. 2023, 14, 156. 36828819 10.1038/s41419-023-05689-0 PMC9958101

