# Supplementary material for: MeCP2 Lactylation Protects against Ischemic Brain Injury by Transcriptionally Regulating Neuronal Apoptosis
Source: Adv Sci (Weinh). 2025 Apr 24;12(21):2415309. doi: 10.1002/advs.202415309 (PMC12140320; doi:10.1002/advs.202415309)
Supplement: Supplementary file 1 — Supporting Information [file ADVS-12-2415309-s001.pdf]

ADVANCED SCIENCE
Open Access

Supporting Information

for *Adv. Sci.*, DOI 10.1002/advs.202415309

MeCP2 Lactylation Protects against Ischemic Brain Injury by Transcriptionally Regulating Neuronal Apoptosis

Min Sun, Yuxin Zhang, Rui Mao, Yan Chen, Pinyi Liu, Lei Ye, Siyi Xu, Junqiu Jia, Shu Shu, Huiya Li, Yanping Yin, Shengnan Xia, Yanting Chen and Yun Xu**

Supporting Information

MeCP2 Lactylation Protects Against Ischemic Brain Injury by Transcriptionally Regulating Neuronal Apoptosis

Min Sun [1,10], Yuxin Zhang [2,10], Rui Mao [1,3,4,5,6,10], Yan Chen [1,3,4,5,6], Pinyi Liu [1,3,4,5,6], Lei Ye [1,3,4,5,6], Siyi Xu [7], Junqiu Jia [8], Shu Shu [1,3,4,5,6], Huiya Li [1,3,4,5,6], Yanping Yin [2], Shengnan Xia [1,3,4,5,6], Yanting Chen [1,3,4,5,6,*] and Yun Xu [1,2,3,4,5,6,9,11,*]

1 Department of Neurology, Nanjing Drum Tower Hospital, Affiliated Hospital of Medical School, Nanjing University, Nanjing 210008, China

2 Nanjing Drum Tower Hospital Clinical College of Nanjing University of Chinese Medicine, Nanjing 210008, China

3 State Key Laboratory of Pharmaceutical Biotechnology and Institute of Translational Medicine for Brain Critical Diseases, Nanjing University, Nanjing 210008, China

4 Jiangsu Key Laboratory for Molecular Medicine and Institute of Translational Medicine for Brain Critical Diseases, Nanjing University, Nanjing 210008, China

5 Jiangsu Provincial Key Discipline of Neurology, Nanjing 210008, China

6 Nanjing Neurology Clinical Medical Center, and Nanjing Gulou Hospital Brain disease and brain Science Center, Nanjing 210008, China

7 Nanjing Drum Tower Hospital Clinical College of Jiangsu University, Nanjing 210008, China

8 Department of Neurology, Nanjing Drum Tower Hospital, Chinese Academy of Medical Science & Peking Union Medical College, Nanjing 210008, China

9 Nanjing Key Laboratory for Cardiovascular Information and Health Engineering Medicine, Nanjing 210008, China

10 These authors contributed equally

11 Lead contact

*Correspondence: y.chen860606@gmail.com (Y.C.), xuyun20042001@aliyun.com (Y.X.)

Supplemental Results

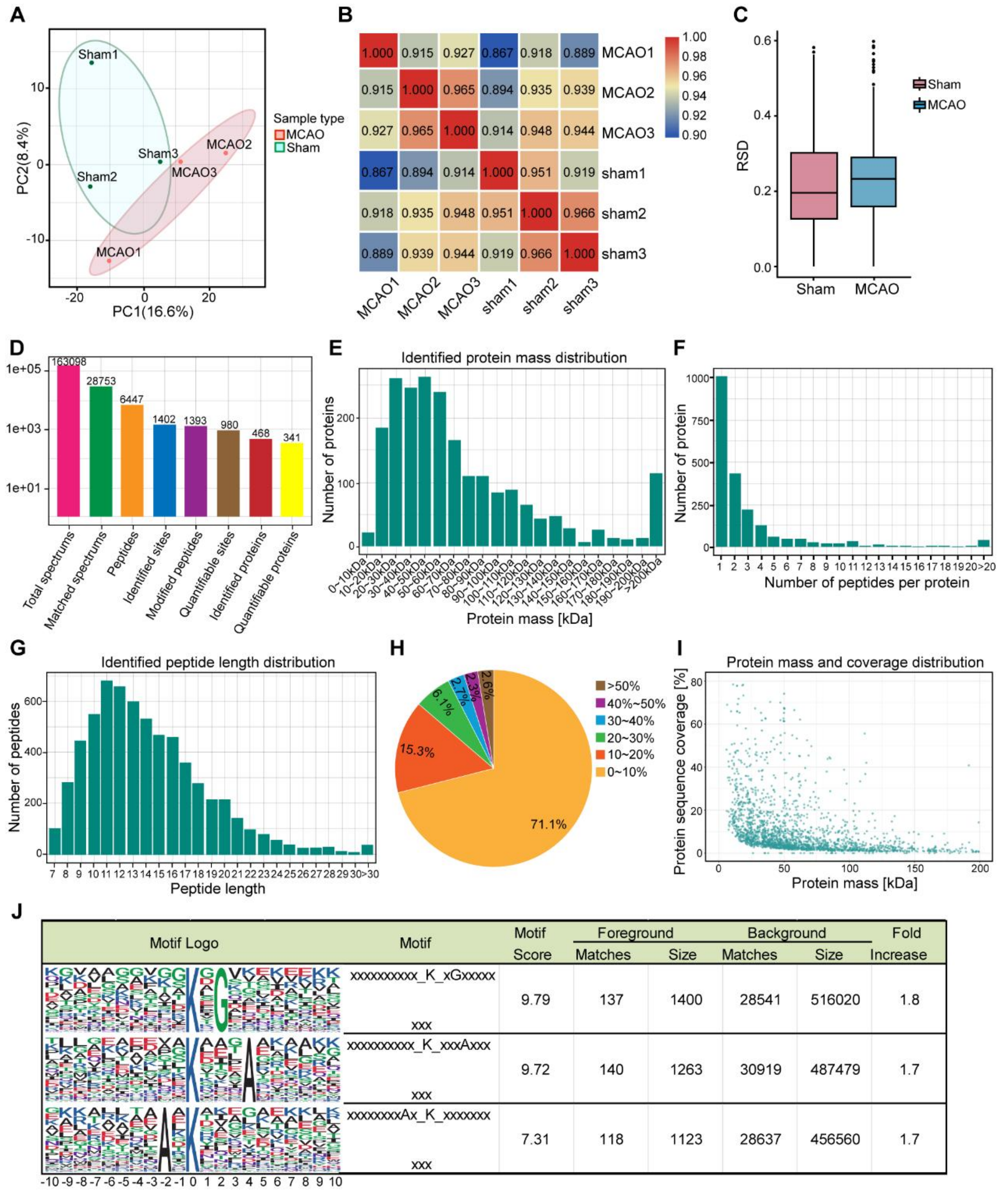

Figure S1. Quality control of lactylation modification in sham and MCAO mice.

(A) Principal component analysis (PCA) plot differentiating MCAO and sham mouse brain samples based on lactylation profiles.

(B) Pearson's correlation analysis with each value that represents the correlation coefficient between the two samples.

(C) Boxplot comparing the relative standard deviation (RSD) of protein Kla intensity between MCAO and sham groups.

(D) Overview of the proteomics dataset.

(E) Distribution of identified proteins based on their molecular mass.

(F) Histogram depicting the number of peptides identified per protein.

(G) Distribution of identified peptide lengths across all samples.

(H) Pie chart showing the distribution of proteins sequence coverage.

(I) Scatter plot illustrating the relationship between protein size and coverage in the dataset.

(J) Motif analysis identifying the top enriched lactylation motifs in the dataset.

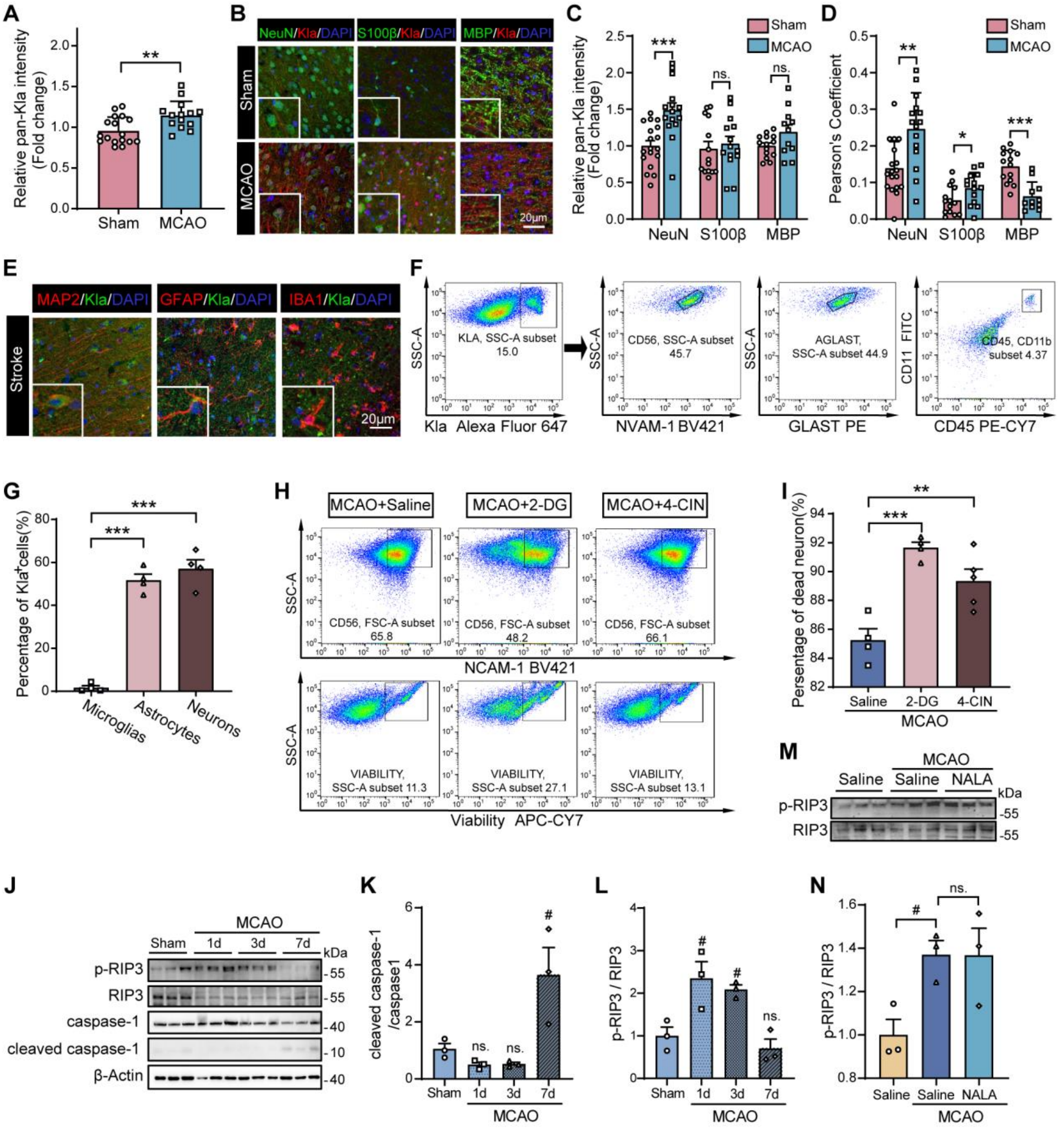

Figure S2. Neuronal protein lactylation and its role in regulating neuronal death in MCAO mice

(A) Quantification of pan-Kla intensity in sham and MCAO brain sections, showing increased lactylation in the penumbra 1 d after reperfusion (n = 11-18 slices from 4-6 mice per group).

(B) Representative immunofluorescence images showing colocalization of pan-Kla (red) with NeuN, S100β, or MBP (green) in the cortex of sham and MCAO mice.

(C) Quantification of pan-Kla intensity in NeuN$^+$ neurons, S100β$^+$ astrocytes or MBP$^+$ oligodendrocytes in sham and MCAO brain tissues (n = 11-18 slices from 4-6 mice per group).

(D) Pearson's correlation coefficient showing the relationship between pan-Kla intensity and NeuN, S100β, or MBP expression in sham and MCAO tissues (n = 11-18 slices from 4-6 mice per group).

(E) Representative images of pan-Kla (green) colocalization with MAP2, GFAP, or IBA1 (red) in brain tissues of stroke patients.

(F) Flow cytometry gating strategy for isolating Kla^+ cells from MCAO brain tissues, including neurons ($NeuN^+$), astrocytes ($GLAST^+$), and microglia ($CD45^+/CD11b^+$).

(G) Quantification of Kla^+ cells across different cell types (neurons, astrocytes, microglia) in MCAO brain tissues (n = 4 per group).

(H) Flow cytometry analysis showing the effects of 2-DG and 4-CIN treatment on neuronal death in MCAO brain tissues.

(I) Quantification of dead cells ($Viability^+$) in MCAO brain tissues treated with saline, 2-DG, or 4-CIN (n = 4-5 per group).

(J-L) Western blot analysis (L) and quantification of cleaved caspase-1 (K) and p-RIP3 levels in sham and MCAO brain tissues at 1, 3, and 7 d post-MCAO (n = 3 per group).

(M and N) Western blot analysis (M) and quantification (N) of p-RIP3 in brain tissues of MCAO mice treated with saline or sodium lactate (NALA) (n = 3 per group).

Data are shown as mean ± SEM. $^{\#}P < 0.05$ *vs.* sham control; $^{**}P < 0.01$, $^{***}P < 0.001$ *vs.* MCAO control; ns., not significant.

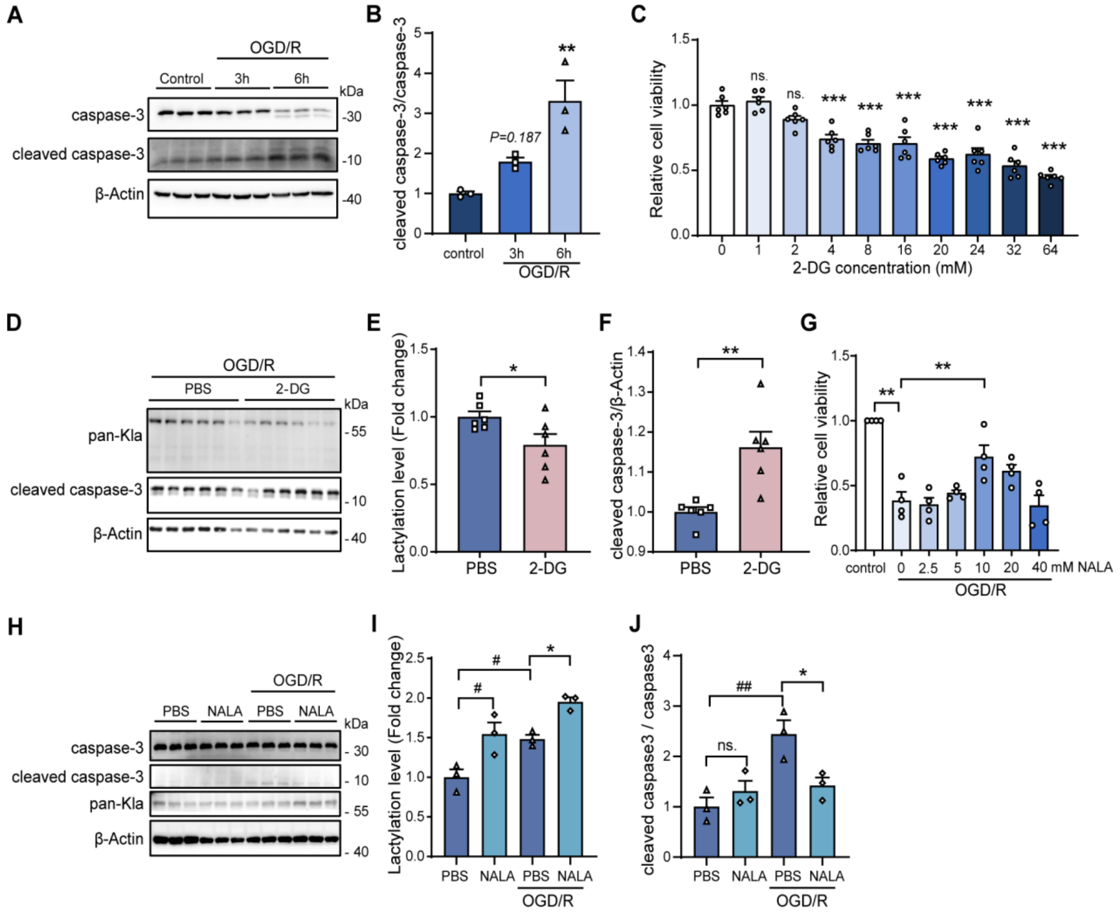

Figure S3. Role of lactylation in neuronal apoptosis under OGD/R conditions

(A) Western blot analysis of caspase-3 and cleaved caspase-3 in primary neurons subjected to OGD/R for 3 and 6 h.

(B) Quantification of cleaved caspase-3 in neurons under OGD/R (n = 3 per group).

(C) Dose-response analysis of cell viability in neurons treated with increasing concentrations of

2-DG under OGD/R conditions, showing reduced viability at higher concentrations (n = 6 per group).

(D) Western blot analysis of pan-Kla and cleaved caspase-3 levels in neurons treated with 2-DG compared to PBS under OGD/R.

(E, F) Quantification of lactylation levels (E) and cleaved caspase-3 relative to β-actin (F), demonstrating decreased lactylation and increased apoptosis with 2-DG treatment (n = 6 per group).

(G) Dose-response analysis of cell viability in neurons treated with increasing concentrations of NALA under OGD/R, confirming reduced viability at higher concentrations (n = 3 and 4 per group).

(H) Western blot analysis of pan-Kla and cleaved caspase-3 levels in neurons treated with NALA compared to PBS under OGD/R.

(I, J) Quantification of lactylation levels (I) and cleaved caspase-3 (J), indicating increasd lactylation and reduced apoptosis with NALA treatment (n = 3 per group).

Data are presented as mean ± SEM. $^{\#}P < 0.05$, $^{\#\#}P<0.01$ *vs.* sham control; *$P < 0.05$; **$P < 0.01$, ***$P < 0.001$; ns., not significant.

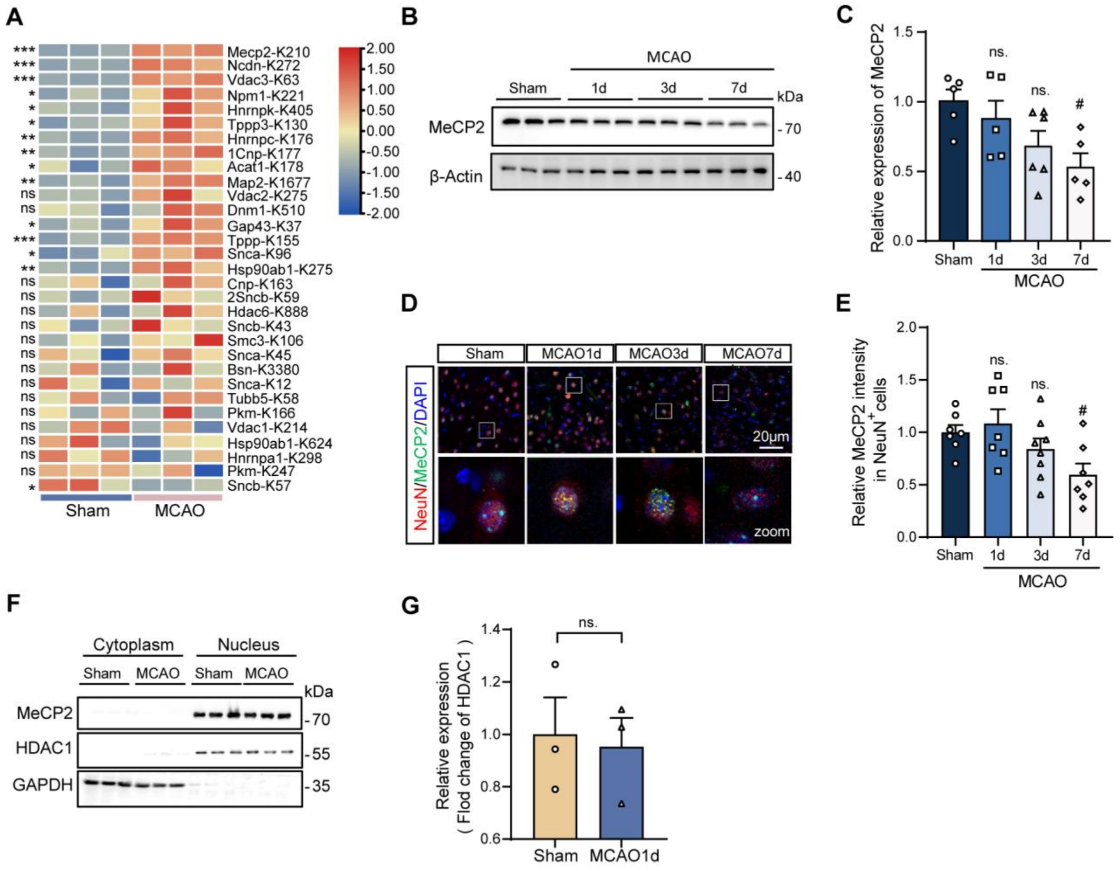

Figure S4. MeCP2 expression and lactylation in the cortex after stroke

(A) Heatmap based on PRM data showing differentially lactylated proteins between sham and MCAO groups.

(B) Western blot analysis of MeCP2 expression in sham and MCAO brain tissues at 1, 3, and 7 d post-MCAO.

(C) Quantification of MeCP2 expression levels in the cortex of sham and MCAO mice (n = 5-6

per group).
(D) Representative immunofluorescence images of MeCP2 (green) co-stained with NeuN (red) in the cortex of sham and MCAO mice at different time points.
(E) Quantification of MeCP2 fluorescence intensity in neurons over time post-MCAO (n = 3 per group).
(F) Western blot analysis of MeCP2 in cytoplasmic and nuclear fractions of sham and MCAO brain tissues.
(G) Quantification of MeCP2 expression in nuclear fractions relative to HDAC1, showing no significant change of MeCP2 expression at 1 d post-MCAO (n = 3 per group).
Data are presented as mean ± SEM. $^{\#}P < 0.05$ *vs.* sham control; ns., not significant.

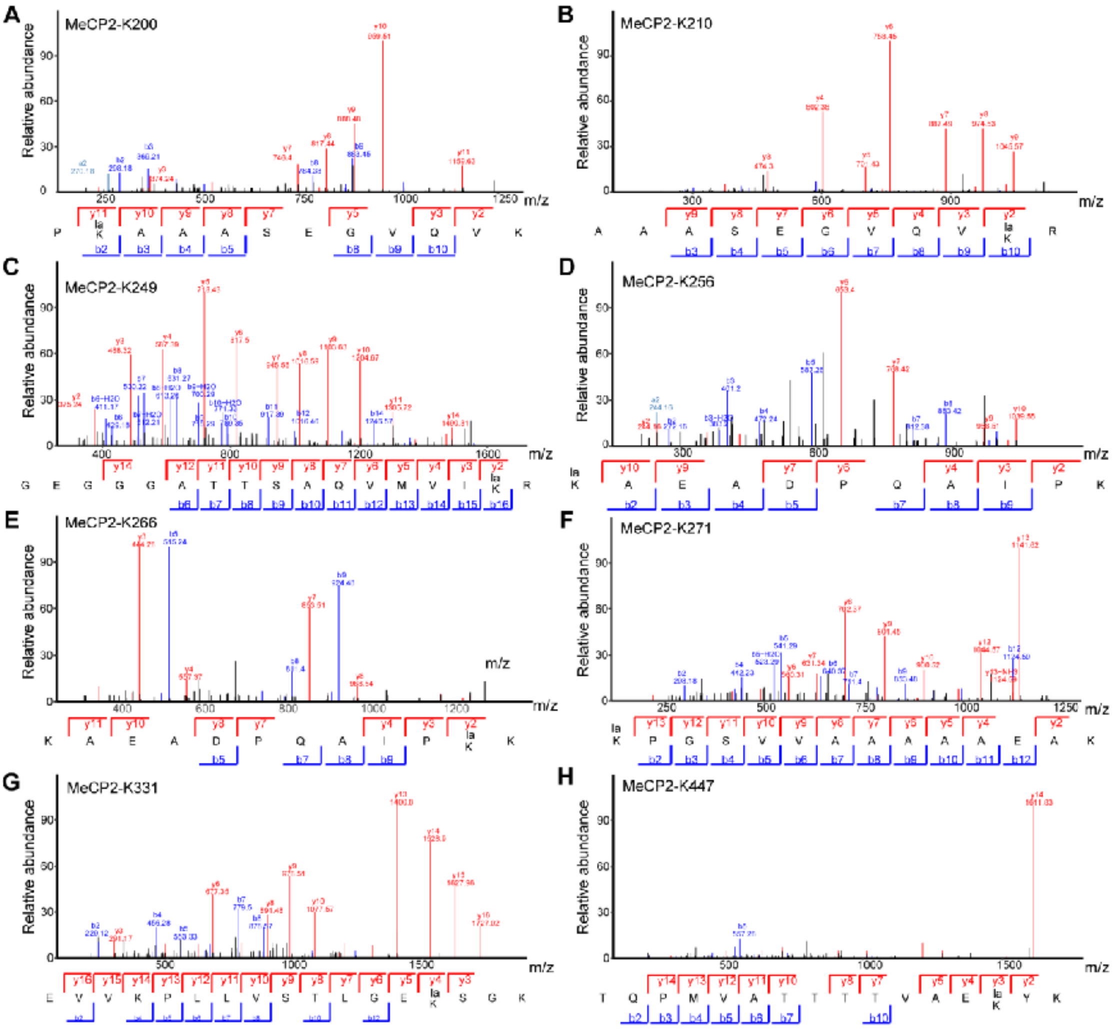

Figure S5. LC-MS/MS spectra identifying lactylation sites on MeCP2
(A–H) Representative MS/MS spectra confirming lactylation at specific lysine residues on MeCP2, including K200 (A), K210 (B), K249 (C), K256 (D), K266 (E), K271 (F), K331 (G), and K447 (H).

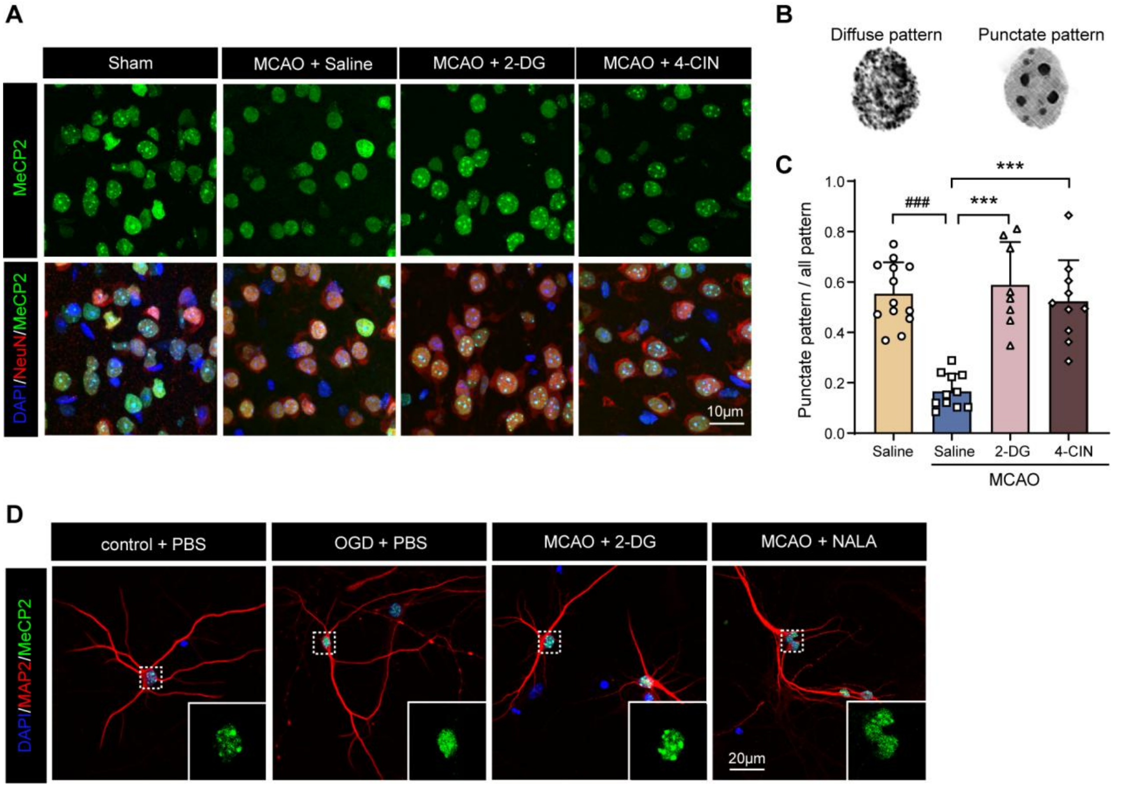

Figure S6. Metabolic regulation of MeCP2 nuclear distribution patterns in stroke models

(A) Immunofluorescence analysis showing nuclear localization of MeCP2 in cortical neurons of sham and MCAO mice treated with saline, 2-DG, or 4-CIN at 1 d after reperfusion.

(B) Schematic representation of the two typical MeCP2 nuclear patterns: diffuse and punctate.

(C) Quantification of the punctate pattern relative to all patterns in neurons under different treatment conditions (n = 8-13 per group), showing increased ratios of punctate patterns with 2-DG and 4-CIN treatments under MCAO conditions.

(D) Representative images showing MeCP2 (green) and neuronal marker MAP2 (red) localization in control neuronal cultures and OGD neurons treated with PBS, 2-DG, or NALA.

Data are presented as mean ± SEM. $^{###}P<0.001$ *vs.* sham control; $^{***}P < 0.001$ *vs.* MCAO control; ns., not significant.

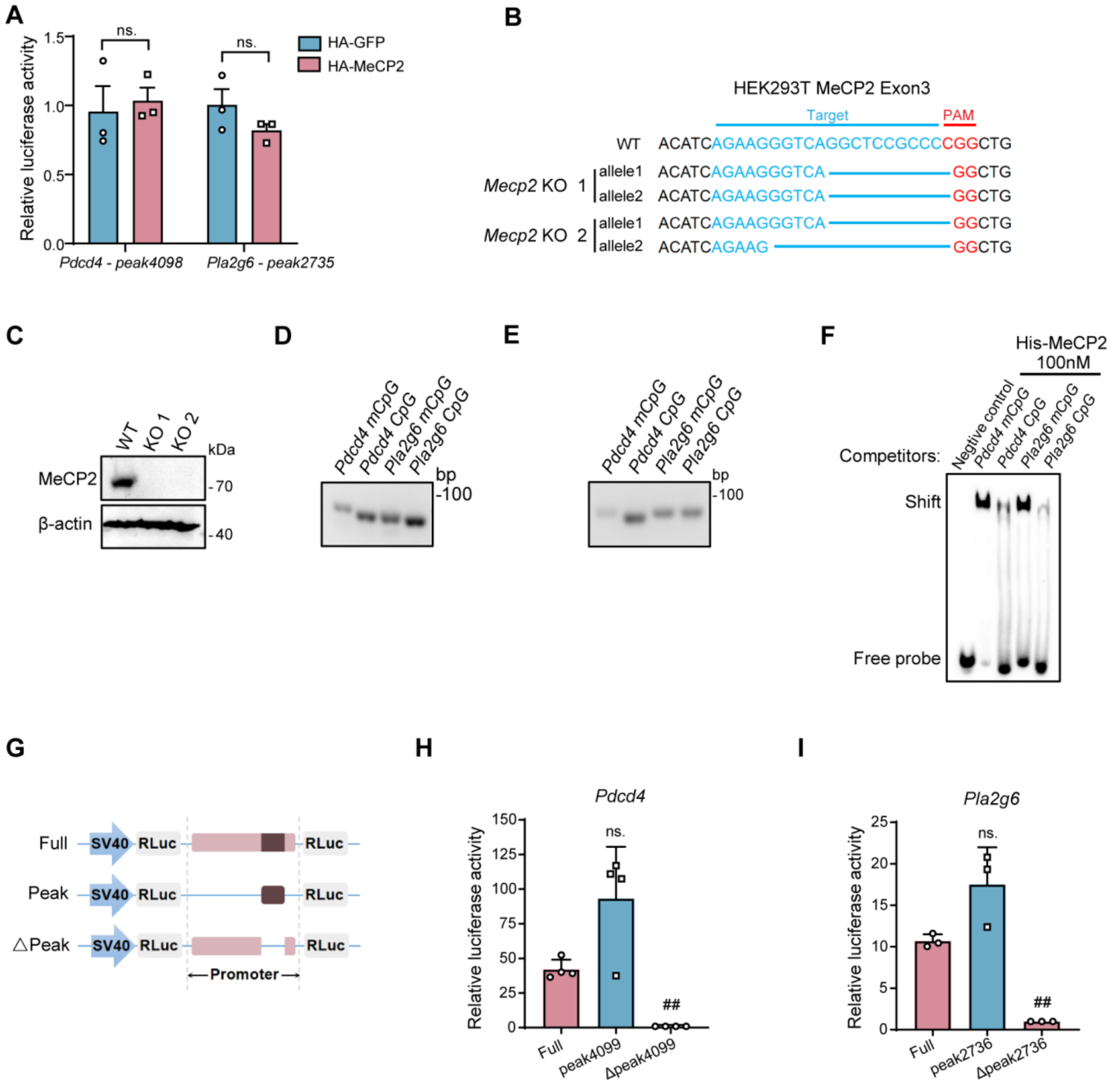

Figure S7. MeCP2 regulates transcriptional activity of *Pdcd4* and *Pla2g6*

(A) Luciferase reporter assays showing the relative transcriptional activity of *Pdcd4* and *Pla2g6* promoters at peak regions in HEK293T cells expressing HA-GFP or HA-MeCP2 (n = 3 per group).

(B) CRISPR/Cas9-mediated deletion of *Mecp2* in HEK293T cells, targeting exon 3, with sequences of wild-type and MeCP2 knockout alleles shown.

(C) Western blot confirming the loss of MeCP2 expression in two independent *Mecp2* knockout clones (KO1 and KO2) compared to wild-type (WT).

(D and E) Agarose gel electrophoresis showing the competitor probes used in the EMSA analysis for *Pdcd4* and *Pla2g6* promoter regions.

(F) Electrophoretic mobility shift assays (EMSA) demonstrating the binding of human recombinant MeCP2 proteins to mCpG and CpG motifs in *Pdcd4* and *Pla2g6* promoter regions.

(G) Schematic representation of luciferase constructs containing full promoter, peak, or Δpeak regions.

(H and I) Luciferase activity assays for *Pdcd4* (E) and *Pla2g6* (F) promoter constructs (Full, Peak, ΔPeak) performed in HEK293T cells (n = 3 and 4 per group).

Data are presented as mean ± SEM. $^{\#\#}P<0.01$ *vs.* control; ns., not significant.

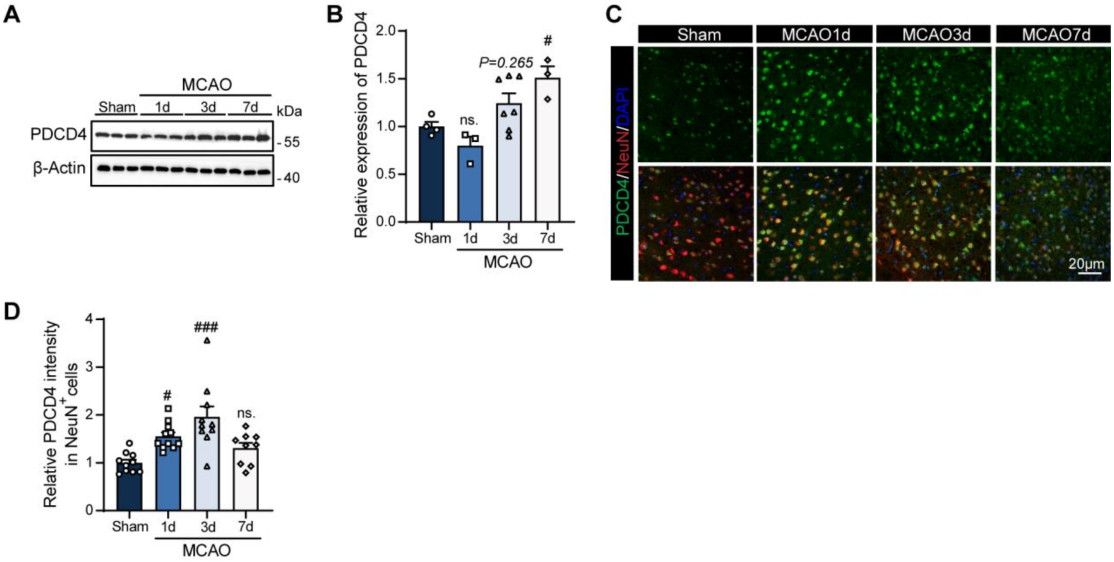

Figure S8. PDCD4 expression patterns in brain tissues and neurons following ischemic stroke

(A) Western blot analysis of PDCD4 expression in sham and MCAO brain tissues at 1, 3, and 7 d following reperfusion, showing a time-dependent increase in PDCD4 levels.

(B) Quantification of PDCD4 protein levels in the cortex of sham and MCAO mice (n = 3-7 per group).

(C) Representative immunofluorescence images showing the co-localization of PDCD4 (green) and NeuN (red) in the cortex of sham and MCAO mice.

(D) Quantification of PDCD4 fluorescence intensity in neurons over time post-MCAO (n = 9-11 per group).

Data are presented as mean ± SEM. $^{\#}P < 0.05$, $^{\#\#\#}P<0.001$ *vs.* sham control; ns., not significant.

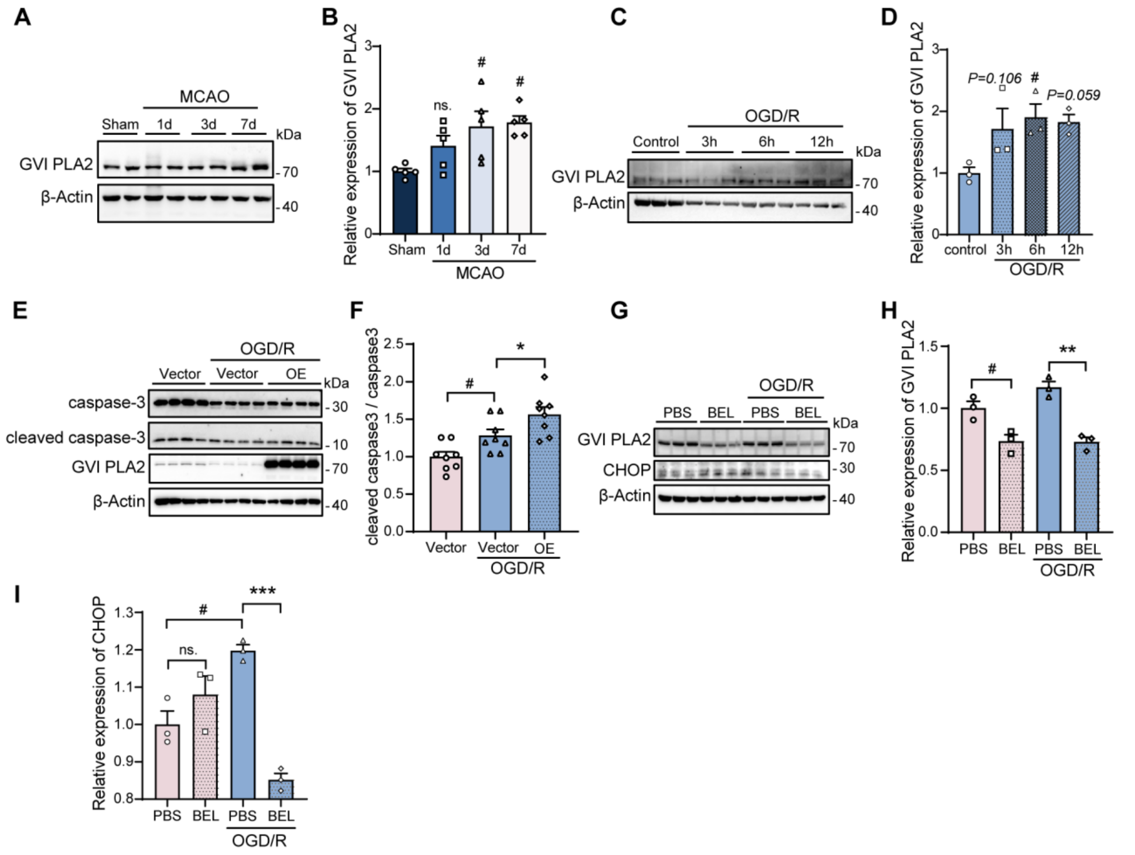

Figure S9. Expression dynamics of GVI PLA2 in stroke models *in vivo* and *in vitro*

(A) Western blot analysis of GVI PLA2 expression in sham and MCAO brain tissues at 1, 3, and 7 d following reperfusion, a time-dependent increase in GVI PLA2 levels.

(B) Quantification of GVI PLA2 protein levels in the cortex of sham and MCAO mice (n = 5 per group).

(C) Western blot analysis of GVI PLA2 expression in primary neurons subjected to OGD/R for 3, 6, and 12 h, demonstrating an early upregulation.

(D) Quantification of GVI PLA2 expression relative to control at different time points under OGD/R conditions (n = 3 per group).

(E) Western blot analysis of caspase-3, cleaved caspase-3, and GVI PLA2 in neuronal cultures overexpressing (OE) GVI PLA2 or vector control under OGD/R conditions.

(F) Quantification of cleaved caspase-3 in primary neurons subjected to OGD/R, indicating enhanced apoptosis with GVI PLA2 overexpression (n = 8 per group).

(G) Western blot analysis of GVI PLA2 and CHOP in neurons treated with BEL (GVI PLA2 inhibitor) or PBS under OGD/R conditions, showing inhibition of GVI PLA2 by BEL.

(H and I) Quantification of GVI PLA2 (H) and CHOP (I) expression in neurons treated with BEL or PBS under OGD/R conditions (n = 3 per group).

Data are presented as mean ± SEM. Data are presented as mean ± SEM. $^{\#}P$, *P < 0.05; **P <0.01; ***P < 0.001; ns., not significant.

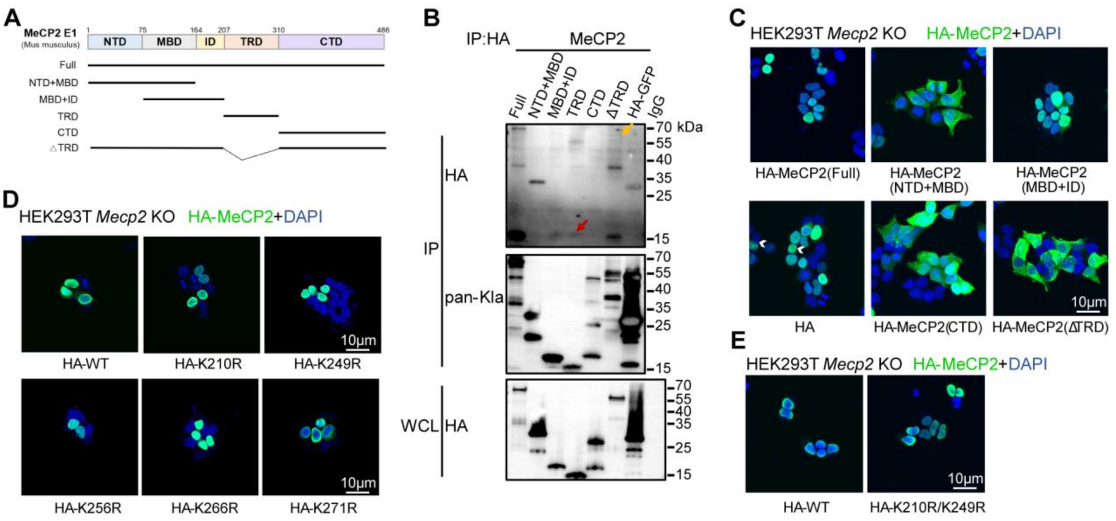

Figure S10. MeCP2 lactylation predominantly occurs at the transcriptional repressor domain

(A) Schematic representation of MeCP2 protein domains and recombinant constructs.

(B) Co-immunoprecipitation assay demonstrating that MeCP2 lactylation primarily occurs at the TRD domain. The positions of the full-length and truncated proteins are indicated.

(C) Immunofluorescence analysis of *Mecp2* KO HEK293T cells transfected with HA-tagged MeCP2 constructs. Cells were stained with HA (green) and DAPI (blue).

(D) Immunofluorescence analysis of *Mecp2* KO HEK293T cells transfected with different HA-tagged MeCP2 constructs (HA-WT, HA-K210R, HA-K249R, HA-K256R, HA-K266R, HA-K271R). Cells were stained with HA (green) and DAPI (blue). .

(E) Immunofluorescence analysis of *Mecp2* KO cells transfected with WT MeCP2 or the K210R/K249R MeCP2 mutant. Cells were stained with HA (green) and DAPI (blue).

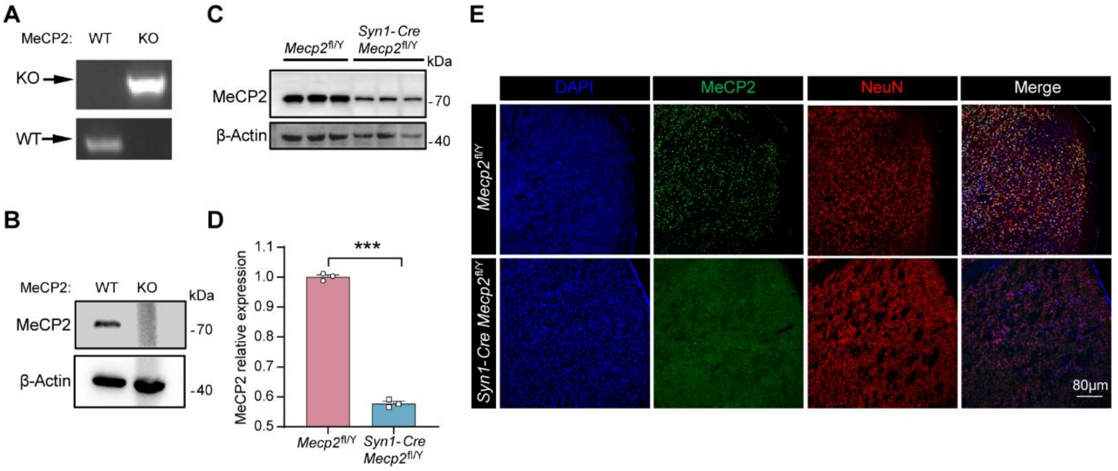

Figure S11. Validation of *Mecp2* knockout in conditional and constitutive mouse models

(A) PCR genotyping confirming the successful knockout (KO) of the *Mecp2* gene in constitutive MeCP2 KO mice. Bands corresponding to WT and KO alleles are indicated.

(B) Western blot analysis showing the absence of MeCP2 protein in brain tissue from *Mecp2* KO mice compared to WT controls.

(C) Western blot analysis showing decreased MeCP2 expression in brain tissue from Syn1-Cre

Mecp2 $^{flox/Y}$ mice.

(D) Quantification of MeCP2 protein levels relative to β-actin, demonstrating a significant reduction in MeCP2 expression in Syn1-Cre *Mecp2* $^{flox/Y}$ mice (n = 3 per group).

(E) Immunofluorescence images of brain sections from *Mecp2* $^{flox/Y}$ and Syn1-Cre *Mecp2* $^{flox/Y}$ mice, showing diminished MeCP2 expression in the Syn1-Cre *Mecp2* $^{flox/Y}$ group.

Data are presented as mean ± SEM. ***$P < 0.001$ vs. *Mecp2* $^{flox/Y}$ control.

A

Dilution	Lactyl-Mecp2 (K210) peptide A	Lactyl-Mecp2 (K210) peptide B	Control peptide
1 : 6K	1.657	1.67	0.111
1 : 18K	1.644	1.635	0.098
1 : 54K	1.497	1.617	0.066
1 : 162K	1.118	1.22	0.06
1 : 486K	0.724	0.788	0.063

B

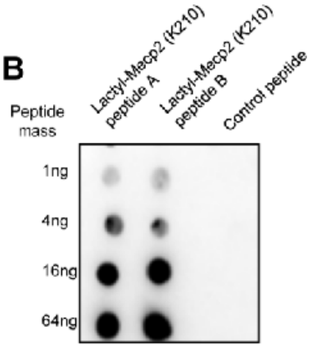

C

Lactyl-Mecp2 (K210) peptide A: CSEGVQV-(lactyl)K-RVLEKS
Lactyl-Mecp2 (K210) peptide B: CEGVQV-(lactyl)K-RVLEK
Control peptide: CSEGVQVKRVLEKS

D

Dilution	Lactyl-Mecp2 (K249) peptide A	Lactyl-Mecp2 (K249) peptide B	Control peptide
1 : 6K	1.927	2.009	1.123
1 : 18K	1.787	1.82	0.546
1 : 54K	1.199	1.252	0.247
1 : 162K	0.621	0.641	0.126
1 : 486K	0.287	0.285	0.081

E

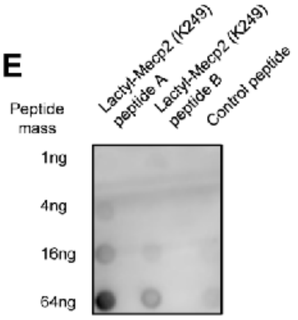

F

Lactyl-Mecp2 (K249) peptide A: CAQVMVI-(lactyl)K-RPGRKR
Lactyl-Mecp2 (K249) peptide B: CVMVI-(lactyl)K-RPGRKR
Control peptide: CAQVMVIKRPGRKR

Figure S12. Verification of MeCP2 lactylation-specific antibodies related to Figure 6

(A) ELISA quantification of lactyl-MeCP2 (K210) peptides A and B compared to a control peptide at various dilution ratios.

(B) Dot blot detection of lactyl-MeCP2 (K210) peptides A and B across different peptide masses.

(C) Peptide sequences used to generate lactylation-specific antibodies for K210.

(D) ELISA quantification of lactyl-MeCP2 (K249) peptides A and B compared to a control peptide at various dilution ratios.

(E) Dot blot detection of lactyl-MeCP2 (K249) peptides A and B across different peptide masses.

(F) Peptide sequences used to generate lactylation-specific antibodies for K249.

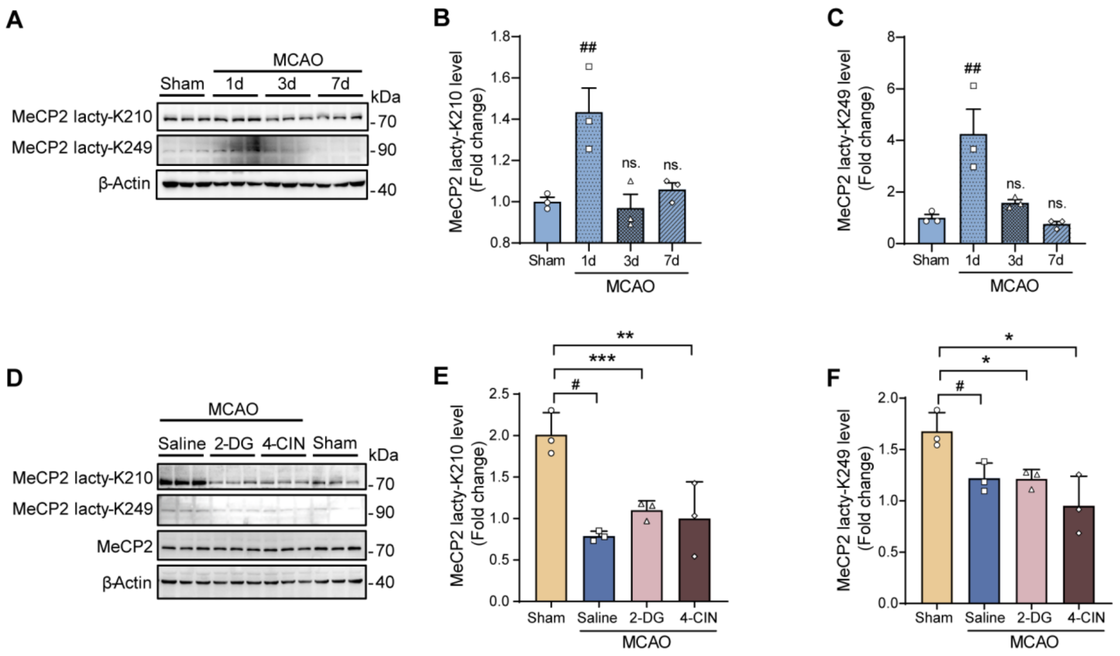

Figure S13. Dynamic regulation of MeCP2 K210 and K249 lactylation after ischemic stroke

(A) Western blot analysis showing MeCP2 lactylation at K210 and K249 in the cortex from sham and MCAO mice at 1, 3, and 7 d following reperfusion.

(B and C) Quantification of MeCP2 lactylation levels at K210 (B) and K249 (C) in sham and MCAO groups (n = 3 per group).

(D) Western blot analysis of MeCP2 lactylation at K210 and K249 in MCAO mice treated with saline, 2-DG, or 4-CIN.

(E, F) Quantification of MeCP2 lactylation levels at K210 (E) and K249 (F) in the different treatment groups (n = 3 per group).

Data are presented as mean ± SEM. $^{\#}P < 0.05$, $^{\#\#}P<0.01$ *vs.* sham control; *$P< 0.05$, **$P < 0.01$, ***$P < 0.001$ *vs.* MCAO control; ns., not significant.

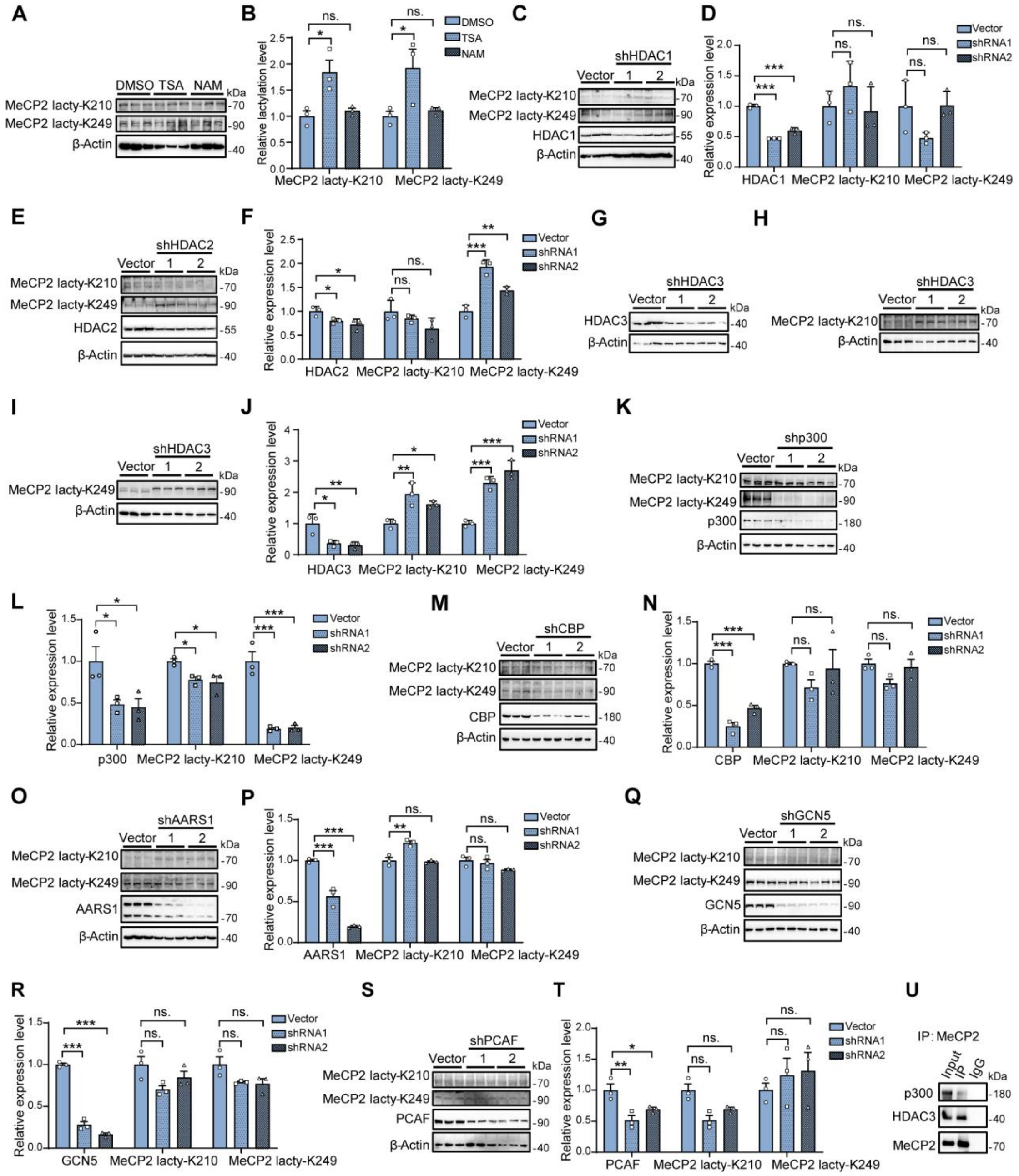

Figure S14. Regulation of MeCP2 K210 and K249 lactylation by histone deacetylases and acetyltransferases

(A and B) Western blot analysis and quantification of MeCP2 K210 and K249 lactylation in Neuro-2a cells treated with Trichostatin A (TSA) or nicotinamide (NAM).

(C-T) Western blot analysis and quantification of MeCP2 K210 and K249 lactylation in Neuro-2a cells with HDAC1 (C and D), HDAC2 (E and F), HDAC3 (G-J), P300 (K and L), CBP (M and N), AARS1 (O and P), GCN5 (Q and R), or PCAF knockdown (S and T).

(U) Co-immunoprecipitation analysis showing MeCP2 interaction with P300 and HDAC3, supporting their involvement in regulating MeCP2 lactylation.

Data are presented as mean ± SEM of independent experiments (n = 3 biological replicates). **P* < 0.05, ***P*< 0.01, ****P* < 0.001; ns., not significant.

Supplementary Information

Table S1

Differentially expressed lactylated proteins between sham group and MCAO group (n = 3)

Accession	Position	Amino acid	Gene name	MCAO1	MCAO2	MCAO3	Sham1	Sham2	Sham3	Regulated Type	Subcellular localization	Relative level of lactylated protein in MCAO compared to Sham	
												Fold change	*P* value
O08756	107	K	*Hsd17b10*	-	-	-	0.971	0.75	1.347	Down	mitochondria	0.001	0.01
Q80SW1	478	K	*Ahcyl1*	-	-	-	0.696	0.864	1.188	Down	cytoplasm	0.001	0.01
Q9R0P5	22	K	*Dstn*	1.008	1.362	1.317	0.74	0.717	0.999	Up	mitochondria	1.501	0.045233689
P52480	166	K	*Pkm*	-	1.363	1.25	0.753	0.793	1.033	Up	cytoplasm	1.52	0.046532467
O55042	34	K	*Snca*	1.101	1.398	1.226	0.768	0.752	0.926	Up	extracellular	1.523	0.011735582
Q9Z2V5	888	K	*Hdac6*	1.141	1.264	1.13	0.887	0.646	-	Up	nucleus	1.537	0.040138924
Q6PIC6	619	K	*Atp1a3*	1.23	1.294	1.128	0.666	0.75	0.943	Up	plasma membrane	1.548	0.01525259
P10637	677	K	*Mapt*	-	1.43	1.232	0.815	0.799	0.955	Up	nucleus	1.554	0.017024439
P10637	667	K	*Mapt*	1.079	1.452	1.27	0.881	0.82	0.722	Up	nucleus	1.569	0.012614385
P10637	573	K	*Mapt*	1.107	1.451	1.27	0.95	0.766	0.692	Up	nucleus	1.59	0.018628079
P08553	803	K	*Nefm*	1.2	1.425	1.012	-	0.704	0.808	Up	nucleus	1.604	0.0430547
P05064	147	K	*Aldoa*	1.045	1.445	1.309	0.798	0.731	0.835	Up	cytoplasm	1.607	0.010746288
P31650	612	K	*Slc6a11*	1.093	1.533	1.22	0.727	0.7	0.96	Up	plasma membrane	1.611	0.027605875
Q6PER3	151	K	*Mapre3*	1.366	1.362	1.063	0.967	0.679	0.697	Up	cytoplasm	1.618	0.025746524
P97825	8	K	*Jpt1*	-	1.363	1.419	0.807	0.911	-	Up	nucleus	1.619	0.016994364
Q9Z204	176	K	*Hnrnpc*	1.054	1.424	1.256	0.66	-	0.836	Up	nucleus	1.664	0.037901034
Q61937	237	K	*Npm1*	-	1.412	1.388	0.86	0.854	0.786	Up	nucleus	1.68	0.000836331
Q9CRB6	130	K	*Tppp3*	1.151	1.422	1.291	0.677	0.663	0.934	Up	cytoplasm	1.699	0.013079685
Q61990	115	K	*Pcbp2*	1.113	1.474	1.262	0.794	0.677	0.783	Up	cytoskeleton	1.708	0.005201382
P63260	328	K	*Actg1*	0.999	1.509	1.362	0.584	0.718	0.94	Up	cytoskeleton	1.726	0.04123282
P80316	265	K	*Cct5*	1.179	1.456	1.204	0.693	0.795	0.731	Up	cytoplasm	1.73	0.002217055
O08997	60	K	*Atox1*	1.186	1.42	1.336	0.745	0.649	0.875	Up	extracellular	1.737	0.005320807
P99024	58	K	*Tubb5*	1.347	1.386	1.161	0.544	0.828	0.859	Up	cytoplasm,nucleus	1.745	0.021381434
O55042	12	K	*Snca*	1.1	1.577	1.318	0.54	0.712	0.977	Up	extracellular	1.792	0.039769335
P68372	252	K	*Tubb4b*	1.156	1.529	-	0.682	0.7	0.858	Up	cytoplasm,nucleus	1.798	0.025341955
P16330	163	K	*Cnp*	-	1.484	1.343	0.668	0.693	0.987	Up	mitochondria	1.806	0.034904953
Q91ZZ3	57	K	*Sncb*	1.453	1.545	1.172	0.619	0.756	0.925	Up	cytoplasm	1.813	0.013637921
P06837	37	K	*Gap43*	0.983	1.449	1.296	0.622	0.703	-	Up	cytoplasm	1.876	0.028599763
O55042	43	K	*Snca*	-	1.543	1.396	0.706	-	0.84	Up	extracellular	1.901	0.023335674
Q91ZZ3	59	K	*Sncb*	1.452	1.611	1.223	0.78	0.665	0.807	Up	cytoplasm	1.903	0.003056917
P08553	221	K	*Nefm*	-	1.609	1.296	0.663	0.626	0.983	Up	nucleus	1.918	0.044700887
O55042	96	K	*Snca*	1.183	1.672	1.266	0.575	0.7	0.851	Up	extracellular	1.938	0.012854391
Q61937	221	K	*Npm1*	1.087	1.517	1.219	0.613	-	0.696	Up	nucleus	1.947	0.016395884

Q91ZZ3	12	K	*Sncb*	1.419	1.667	1.238	0.554	0.734	0.923	Up	cytoplasm	1.956	0.016014412
P11499	275	K	*Hsp90ab1*	1.065	1.638	1.359	0.527	0.615	0.886	Up	cytoplasm	2.003	0.023771715
Q91ZZ3	34	K	*Sncb*	1.452	1.621	1.311	0.653	0.694	0.834	Up	cytoplasm	2.01	0.001861099
Q61792	112	K	*Lasp1*	1.385	1.614	1.265	0.742	0.525	0.805	Up	nucleus	2.058	0.007944456
P62761	7	K	*Vsnl1*	1.217	1.64	1.237	0.52	0.635	0.81	Up	cytoplasm	2.083	0.009937394
Q9D394	420	K	*Rufy3*	1.093	1.734	1.12	0.513	-	0.724	Up	cytoplasm	2.127	0.048873463
Q61937	141	K	*Npm1*	-	1.435	1.508	0.743	0.639	-	Up	nucleus	2.13	0.010775788
Q9R0P4	62	K	*Smap*	1.385	1.759	1.528	0.639	0.684	0.859	Up	nucleus	2.141	0.002504867
O55042	45	K	*Snca*	1.15	1.782	1.377	0.511	0.663	0.818	Up	extracellular	2.163	0.014208885
Q9DCT8	112	K	*Crip2*	1.459	1.791	0.891	0.747	0.668	0.476	Up	nucleus	2.19	0.037165302
Q7TQD2	155	K	*Tppp*	1.348	1.519	1.176	-	0.568	0.638	Up	cytoplasm	2.235	0.004662171
Q62419	76	K	*Sh3gl1*	1.02	1.824	1.374	0.706	0.549	-	Up	nucleus	2.241	0.044616724
Q8QZT1	178	K	*Acat1*	-	1.648	1.357	-	0.561	0.728	Up	mitochondria	2.331	0.034654319
Q8BIZ1	1221	K	*Anks1b*	1.014	1.69	1.332	0.462	-	0.674	Up	nucleus	2.369	0.036140993
Q62261	1633	K	*Sptbn1*	1.144	1.956	1.231	0.459	0.517	0.836	Up	cytoplasm	2.39	0.024274458
P08553	807	K	*Nefm*	1.247	1.669	1.473	0.535	0.596	0.702	Up	nucleus	2.394	0.001652267
Q7TQF7	159	K	*Amph*	1.267	1.553	1.221	-	0.472	0.631	Up	nucleus	2.442	0.008486579
P61979	405	K	*Hnrnpk*	1.139	1.689	1.328	0.6	0.476	-	Up	nucleus	2.575	0.011937295
Q8K0T0	767	K	*Rtn1*	1.253	1.687	1.299	-	0.474	0.62	Up	nucleus	2.583	0.009081731
P39053	510	K	*Dnm1*	1.351	1.737	1.487	0.544	0.455	0.599	Up	cytoplasm	2.863	0.000639475
P97315	112	K	*Csrp1*	1.281	1.663	1.396	-	0.472	0.517	Up	nucleus	2.926	0.00200956
P20357	1698	K	*Map2*	1.077	2.244	1.527	-	0.445	0.635	Up	nucleus	2.993	0.039211345
P20357	1677	K	*Map2*	1.258	2.268	1.637	0.622	0.476	0.618	Up	nucleus	3.009	0.004903462
Q60931	63	K	*Vdac3*	1.256	2.075	1.26	0.533	0.42	0.525	Up	cytoplasm	3.106	0.003786037
P97427	7	K	*Crmp1*	1.142	1.951	1.667	0.528	0.42	0.556	Up	cytoplasm,nucleus	3.165	0.003286027
Q62261	2176	K	*Sptbn1*	1.263	1.875	1.117	-	0.348	0.503	Up	cytoplasm	3.333	0.016269478
Q9Z0E0	272	K	*Ncdn*	1.361	2.017	1.356	0.48	0.402	0.405	Up	plasma membrane	3.678	0.000862598
P11499	607	K	*Hsp90ab1*	1.16	1.597	1.561	-	0.311	0.449	Up	cytoplasm	3.788	0.005951753
P62761	74	K	*Vsnl1*	1.248	2.103	1.655	0.402	0.352	0.402	Up	cytoplasm	4.33	0.00077712
Q91ZZ3	43	K	*Sncb*	2.291	2.074	-	-	0.312	0.271	Up	cytoplasm	7.487	0.001827843
O08583	80	K	*Alyref*	1.087	1.441	0.96	-	-	-	Up	cytoplasm	1000	0.01
O88737	3380	K	*Bsn*	1.008	1.345	1.078	-	-	-	Up	nucleus	1000	0.01
P07724	588	K	*Alb*	0.487	0.784	0.709	-	-	-	Up	extracellular	1000	0.01
P07724	212	K	*Alb*	0.517	0.789	0.676	-	-	-	Up	extracellular	1000	0.01
P07724	549	K	*Alb*	0.502	0.858	0.637	-	-	-	Up	extracellular	1000	0.01
P07724	376	K	*Alb*	0.504	0.887	0.611	-	-	-	Up	extracellular	1000	0.01
P07724	305	K	*Alb*	0.576	0.706	0.681	-	-	-	Up	extracellular	1000	0.01
P09405	116	K	*Ncl*	0.9	1.228	0.988	-	-	-	Up	nucleus	1000	0.01
P09405	71	K	*Ncl*	0.776	1.397	0.991	-	-	-	Up	nucleus	1000	0.01
P09405	469	K	*Ncl*	0.912	1.221	0.978	-	-	-	Up	nucleus	1000	0.01
P11499	624	K	*Hsp90ab1*	0.841	1.281	0.926	-	-	-	Up	cytoplasm	1000	0.01
P20357	1634	K	*Map2*	0.455	2.313	1.438	-	-	-	Up	nucleus	1000	0.01

P23198	92	K	*Cbx3*	0.888	1.339	0.993	-	-	-	Up	nucleus	1000	0.01
P40124	286	K	*Cap1*	0.852	1.219	0.994	-	-	-	Up	cytoplasm	1000	0.01
P51859	39	K	*Hdgf*	0.964	1.501	0.916	-	-	-	Up	nucleus	1000	0.01
P52480	247	K	*Pkm*	0.724	1.527	1.06	-	-	-	Up	cytoplasm	1000	0.01
P60879	103	K	*Snap25*	0.661	1.522	1.026	-	-	-	Up	cytoplasm	1000	0.01
P61089	92	K	*Ube2n*	0.701	1.524	1.098	-	-	-	Up	mitochondria	1000	0.01
P63017	524	K	*Hspa8*	0.807	1.267	1.081	-	-	-	Up	cytoplasm	1000	0.01
P68254	122	K	*Ywhaq*	0.883	1.059	1.182	-	-	-	Up	cytoplasm	1000	0.01
Q5SQX6	425	K	*Cyfip2*	1.172	1.09	0.646	-	-	-	Up	cytoplasm	1000	0.01
Q60930	75	K	*Vdac2*	0.845	1.292	0.887	-	-	-	Up	extracellular	1000	0.01
Q61686	91	K	*Cbx5*	0.882	1.378	0.968	-	-	-	Up	nucleus	1000	0.01
Q6PH08	769	K	*Erc2*	0.878	1.099	1.13	-	-	-	Up	nucleus	1000	0.01
Q6PIC6	458	K	*Atp1a3*	0.598	1.376	1.17	-	-	-	Up	plasma membrane	1000	0.01
Q8VDN2	502	K	*Atp1a1*	0.803	1.06	1.201	-	-	-	Up	plasma membrane	1000	0.01
Q91XV3	52	K	*Basp1*	0.937	0.86	1.069	-	-	-	Up	nucleus	1000	0.01
Q9CW03	106	K	*Smc3*	0.97	1.237	1.11	-	-	-	Up	cytoplasm	1000	0.01
Q9D6R2	177	K	*Idh3a*	0.847	1.381	0.807	-	-	-	Up	cytoplasm	1000	0.01
Q9D8N0	147	K	*Eef1g*	0.758	1.578	0.865	-	-	-	Up	cytoplasm	1000	0.01
Q9DBG3	5	K	*Ap2b1*	0.883	1.157	1.059	-	-	-	Up	plasma membrane	1000	0.01
Q9QYR6	312	K	*Map1a*	1.008	1.805	0.831	-	-	-	Up	nucleus	1000	0.01
Q9WUK2	130	K	*Eif4h*	0.964	1.199	1.209	-	-	-	Up	nucleus	1000	0.01
Q9Z1N5	36	K	*Ddx39b*	0.954	1.252	0.958	-	-	-	Up	cytoplasm,nucleus	1000	0.01
Q9Z2D6	210	K	*Mecp2*	0.889	1.424	1.026	-	-	-	Up	nucleus	1000	0.01
Q9Z2D6	249	K	Mecp2	0.95	1.401	0.968	-	-	-	Up	nucleus	1000	0.01

99 lactylated modified sites were significantly differentially expressed between the Sham group and MCAO group using cut offs of fold change > 1.5/ fold change < 0.67, and *P* value < 0.05

Table S2

Multiple-lactylated proteins upregulated after ischemic stroke

Protein accession	Protein description	Gene name	Number of Kla site
Q9Z2D6	Methyl-CpG-binding protein 2	*Mecp2*	2
P11499	Heat shock protein HSP 90-beta	*Hsp90ab1*	2
P52480	Pyruvate kinase	*Pkm*	2
P62761	Visinin-like protein 1	*Vsnl1*	2
Q62261	Spectrin beta chain, non-erythrocytic 1	*Sptbn1*	2
Q6PIC6	Sodium/potassium-transporting ATPase subunit alpha-3	*Atp1a3*	2
Q61937	Nucleophosmin	*Npm1*	3
P09405	Nucleolin	*Ncl*	3
P08553	Neurofilament medium polypeptide	*Nefm*	3
P10637	Microtubule-associated protein tau	*Mapt*	3
P20357	Microtubule-associated protein 2	*Map2*	3
O55042	Alpha-synuclein	*Snca*	5
Q91ZZ3	Beta-synuclein	*Sncb*	5
P07724	Serum albumin	*Alb*	5

Table S3

GO enrichment analysis, related to Figure2I

**GO Terms Level 1**	**GO Terms ID**	**GO Terms Description**	**Fold Enrichment**	***p* value**	**Gene name**
Cellular Component	GO:0005604	basement membrane	6.83	0.021149818	*Alb*
Cellular Component	GO:0000118	histone deacetylase complex	6.83	0.021149818	*Hdac6/Cbx5*
Cellular Component	GO:0005790	smooth endoplasmic reticulum	6.83	0.021149818	*Rtn1/Map2*
Cellular Component	GO:0010369	chromocenter	6.83	0.021149818	*Cbx3/Cbx5*
Cellular Component	GO:0031618	nuclear pericentric heterochromatin	6.83	0.021149818	*Cbx3/Cbx5*
Cellular Component	GO:0005604	basement membrane	6.83	0.021149818	*Smc3*
Cellular Component	GO:0005721	pericentric heterochromatin	6.83	0.021149818	*Cbx3/Cbx5*
Cellular Component	GO:0045298	tubulin complex	6.83	0.003014567	*Mapt/Tubb4b/Tubb5*
Cellular Component	GO:0035578	azurophil granule lumen	5.12	0.010786817	*Tubb4b/Tubb5/Cap1*
Cellular Component	GO:0005775	vacuolar lumen	4.55	0.005027401	*Tubb5/Tubb4b/Hspa8/Cap1*
Cellular Component	GO:0000792	heterochromatin	4.55	0.005027401	*Mecp2/Cbx5/Cbx3/Aldoa*
Cellular Component	GO:0016234	inclusion body	4.27	0.002290873	*Hdac6/Sncb/Mapt/Nefm/Snca*
Cellular Component	GO:0005720	nuclear heterochromatin	4.1	0.024140957	*Aldoa*
Cellular Component	GO:0005766	primary lysosome	4.1	0.024140957	*Tubb5/Tubb4b/Cap1*
Cellular Component	GO:0042582	azurophil granule	4.1	0.024140957	*Tubb5/Tubb4b/Cap1*
Cellular Component	GO:0005720	nuclear heterochromatin	4.1	0.024140957	*Cbx3/Cbx5*
Cellular Component	GO:0043230	extracellular organelle	3.42	0.018586216	*Hspa8/Actg1/Alb/Aldoa*
Cellular	GO:0070062	extracellular	3.42	0.018586216	*Aldoa/Alb/Hspa8/Actg1*

Component		exosome			
Cellular Component	GO:1903561	extracellular vesicle	3.42	0.018586216	*Aldoa/Alb/Hspa8/Actg1*
Cellular Component	GO:0016363	nuclear matrix	3.42	0.043254513	*Ddx39b/Npm1/Smc3*
Cellular Component	GO:0001917	photoreceptor inner segment	3.42	0.043254513	*Hspa8/Pkm/Dnm1*
Cellular Component	GO:0034399	nuclear periphery	3.1	0.013189462	*Map2/Mapt/Npm1/Ddx39b/Smc3*
Cellular Component	GO:0005875	microtubule associated complex	3.04	0.029798609	*Hdac6/Map1a/Map2/Mapt*
Cellular Component	GO:0030864	cortical actin cytoskeleton	3.04	0.029798609	*Dstn/Sptbn1/Lasp1/Cap1*
Cellular Component	GO:0000790	nuclear chromatin	2.63	0.028951742	*Aldoa/Cbx3/Hnrnpk/Mecp2/Cbx5*
Cellular Component	GO:0000785	chromatin	2.56	0.018967526	*Mecp2/Smc3/Cbx5/Hnrnpk/Cbx3/Aldoa*
Cellular Component	GO:0044304	main axon	2.44	0.039998008	*Map1a/Sptbn1/Dnm1/Map2/Mapt*
Cellular Component	GO:0030863	cortical cytoskeleton	2.44	0.039998008	*Dstn/Sptbn1/Lasp1/Cap1/Bsn*
Cellular Component	GO:0005938	cell cortex	2.36	0.007158514	*Snca/Dstn/Crip2/Sptbn1/Lasp1/Cap1/Ncl/Bsn/Hnrnpk*
Cellular Component	GO:0005730	nucleolus	2.36	0.004641661	*Ddx39b/Sptbn1/Npm1/Cbx5/Jpt1/Hspa8/Ube2n/Map2/Ncl/Cct5*
Cellular Component	GO:0000228	nuclear chromosome	2.28	0.034517694	*Mecp2/Smc3/Cbx5/Hnrnpk/Cbx3/Aldoa*
Cellular Component	GO:0005874	microtubule	2.12	0.024423567	*Hdac6/Map1a/Mapre3/Tubb5/Cct5/Tubb4b/Hspa8/Cnp/Mapt*
Cellular Component	GO:0015629	actin cytoskeleton	2.1	0.007041602	*Sptbn1/Lasp1/Csrp1/Actg1/Snap25/Cap1/Map2/Aldoa/Snca/Amph/Dstn/Hnrnpc*
Cellular Component	GO:0030427	site of polarized growth	2.1	0.038532272	*Map2/Mapt/Gap43/Snca/Snap25/Erc2/Sncb*
Cellular Component	GO:0030426	growth cone	2.1	0.038532272	*Sncb/Erc2/Snap25/Map2/Mapt/Gap43/Snca*
Cellular Component	GO:0030427	site of polarized growth	2.1	0.038532272	*Rufy3/Sncb*
Cellular Component	GO:0030426	growth cone	2.1	0.038532272	*Rufy4/Sncb/Snca*

Cellular Component	GO:0099568	cytoplasmic region	2.09	0.011084582	*Cap1/Dstn/Crip2/Sptbn1/Lasp1/Hnrnpk/Map2/Mapt/Ncl/Bsn/Snca*
Cellular Component	GO:0098984	neuron to neuron synapse	1.98	0.015097579	*Atp1a1/Anks1b/Sh3gl1/Hspa8/Hnrnpk/Map2/Mapt/Nefm/Gap43/Bsn/Map1a*
Cellular Component	GO:1990904	ribonucleoprotein complex	1.95	0.011633436	*Snca/Hsd17b10/Snca/Ncl/Hnrnpc/Ddx39b/Eif4h/Npm1/Cbx5/Tubb5/Hspa8/Hnrnpk/Mapt*
Cellular Component	GO:0005783	endoplasmic reticulum	1.86	0.041430628	*Snca/Hsd17b10/Atp1a1/Rtn1/Atp1a3/Vdac3/Map2/Alb/Ahcyl1*
Cellular Component	GO:0014069	postsynaptic density	1.85	0.047290207	*Map1a/Atp1a1/Anks1b/Hspa8/Hnrnpk/Map2/Mapt/Nefm/Bsn*
Cellular Component	GO:0099572	postsynaptic specialization	1.85	0.047290207	*Nefm/Gap43/Bsn/Mapt/Map1a/Atp1a1/Anks1b/Hspa8/Hnrnpk/Map2*
Cellular Component	GO:0032279	asymmetric synapse	1.85	0.047290207	*Map1a/Atp1a1/Anks1b/Hspa8/Hnrnpk/Map2/Mapt/Nefm/Gap43*
Cellular Component	GO:0005856	cytoskeleton	1.68	0.001610261	*Snap25/Cap1/Cbx3/Map2/Cnp/Mapt/Nefm/Aldoa/Bsn/Snca/Actg1/Hspa8/Tubb4b/Cct5/Hdac6/Hnrnpc/Dstn/Map1a/Smc3/Amph/Mapre3/Sptbn1/Npm1/Lasp1/Tubb5/Csrp1/Tppp*
Cellular Component	GO:0043228	non-membrane-bounded organelle	1.46	0.002308679	*Aldoa/Bsn/Snca/Hsd17b10/Nefm/Ncl/Vdac2/Tubb5/Jpt1/Csrp1/Cct5/Tubb4b/Hspa8/Actg1/Hnrnpk/Ube2n/Cap1/Cbx3/Map2/Cnp/Mapt/Snap25/Cbx5/Lasp1/Npm1/Hdac6/Mecp2/Hnrnpc/Ddx39b/Dstn/Map1a/Smc3/Amph/Tppp/Mapre3/Sptbn1*
Cellular Component	GO:0043232	intracellular non-membrane-bounded organelle	1.46	0.002308679	*Hdac6/Mecp2/Hnrnpc/Ddx39b/Dstn/Map1a/Smc3/Amph/Tppp/Mapre3/Sptbn1/Npm1/Lasp1/Cbx5/Vdac2/Tubb5/Jpt1/Cct5/Hsd17b10/Snca/Bsn/Aldoa/Nefm/Tubb4b/Hspa8/Actg1/Hnrnpk/Ube2n/Snap25/Cap1/Cbx3/Csrp1/Map2/Cnp/Mapt/*

					Ncl
Cellular Component	GO:0031981	nuclear lumen	1.44	0.031087956	*Hdac6/Mecp2/Ddx39b/Ncl/Aldoa/Mapt/Cnp/Map2/Smc3/Anks1b/Sptbn1/Pcbp2/Npm1/Cbx5/Hnrnpc/Jpt1/Cct5/Hspa8/Hnrnpk/Ube2n/Hdgf/Dnm1/Cbx3/Csrp1*
Biological Process	GO:0031123	RNA 3'-end processing	6.72	0.021833603	*Ahcyl1/Hsd17b10*
Biological Process	GO:0010869	regulation of receptor biosynthetic process	6.72	0.021833603	*Hdac6/Hnrnpk*
Biological Process	GO:0070316	regulation of G0 to G1 transition	6.72	0.021833603	*Cbx5/Cbx3*
Biological Process	GO:0090329	regulation of DNA-dependent DNA replication	6.72	0.021833603	*Smc3/Npm1/Mecp2/Map2*
Biological Process	GO:1903649	regulation of cytoplasmic transport	6.72	0.021833603	*Mecp2/Map2*
Biological Process	GO:2000172	regulation of branching morphogenesis of a nerve	6.72	0.021833603	*Mecp2/Hnrnpk*
Biological Process	GO:1901216	positive regulation of neuron death	6.72	0.021833603	*Mapt/Snca*
Biological Process	GO:0031058	positive regulation of histone modification	6.72	0.021833603	*Mecp2/Ube2n*
Biological Process	GO:1903651	positive regulation of cytoplasmic transport	6.72	0.021833603	*Mecp2/Map2*
Biological Process	GO:1990535	neuron projection maintenance	6.72	0.021833603	*Map1a/Atp1a3*

Biological Process	GO:0070317	negative regulation of G0 to G1 transition	6.72	0.021833603	*Cbx5/Cbx3*
Biological Process	GO:0002228	natural killer cell mediated immunity	6.72	0.021833603	*Tubb5/Tubb4b*
Biological Process	GO:0042267	natural killer cell mediated cytotoxicity	6.72	0.021833603	*Tubb5/Tubb4b*
Biological Process	GO:0046785	microtubule polymerization	6.72	0.021833603	*Tppp/Mapt*
Biological Process	GO:0001774	microglial cell activation	6.72	0.021833603	*Mapt/Snca*
Biological Process	GO:0042116	macrophage activation	6.72	0.021833603	*Mapt/Snca*
Biological Process	GO:0001909	leukocyte mediated cytotoxicity	6.72	0.021833603	*Tubb5/Tubb4b*
Biological Process	GO:0070841	inclusion body assembly	6.72	0.021833603	*Hdac6/Mapt*
Biological Process	GO:0060632	regulation of microtubule-based movement	5.38	0.002004094	*Hdac6/Mecp2/Map2/Mapt*
Biological Process	GO:0035036	sperm-egg recognition	5.04	0.011293243	*Vdac2/Cct5/Aldoa*
Biological Process	GO:0051963	regulation of synapse assembly	5.04	0.011293243	*Mecp2/Snap25/Snca*
Biological Process	GO:0031114	regulation of microtubule depolymerization	5.04	0.011293243	*Hdac6/Map1a/Map2*
Biological Process	GO:0031056	regulation of histone modification	5.04	0.011293243	*Mecp2/Ube2n/Snca*
Biological Process	GO:0001578	microtubule bundle formation	5.04	0.011293243	*Tppp3/Tppp/Map2*
Biological Process	GO:0009988	cell-cell recognition	5.04	0.011293243	*Vdac2/Cct5/Aldoa*
Biological Process	GO:0007339	binding of sperm to zona pellucida	5.04	0.011293243	*Vdac2/Cct5/Aldoa*
Biological Process	GO:0008037	cell recognition	4.48	0.005337006	*Vdac2/Cct5/Gap43/Aldoa*
Biological Process	GO:2001020	regulation of response to DNA	4.2	0.002465825	*Ddx39b/Npm1/Hnrnpk/Ube2n/Mapt*

		damage stimulus			
Biological Process	GO:0007338	single fertilization	4.03	0.025226966	*Vdac2/Cct5/Aldoa*
Biological Process	GO:0048013	ephrin receptor signaling pathway	4.03	0.025226966	*Ap2b1/Actg1/Dnm1*
Biological Process	GO:0010948	negative regulation of cell cycle process	3.84	0.011059408	*Cbx5/Cbx3/Npm1/Smc3*
Biological Process	GO:0007613	memory	3.84	0.011059408	*Mecp2/Map1a/Snap25/Mapt*
Biological Process	GO:1901879	regulation of protein depolymerization	3.36	0.019652991	*Hdac6/Dstn/Map1a/Map2*
Biological Process	GO:0043244	regulation of protein complex disassembly	3.36	0.019652991	*Hdac6/Dstn/Map1a/Map2*
Biological Process	GO:1902275	regulation of chromatin organization	3.36	0.045116925	*Mecp2/Ube2n/Snca*
Biological Process	GO:0031623	receptor internalization	3.36	0.019652991	*Ap2b1/Snap25/Dnm1/Snca*
Biological Process	GO:0006611	protein export from nucleus	3.36	0.045116925	*Ddx39b/Ahcyl1/Npm1*
Biological Process	GO:0071805	potassium ion transmembrane transport	3.36	0.045116925	*Atp1a1/Atp1a3/Snap25*
Biological Process	GO:0000226	microtubule cytoskeleton organization	3.36	0.000150026	*Mapt/Nefm/Mapt/Cnp/Map2/Mecp2/Map1a/Tppp3/Tppp/Npm1/Tubb5*
Biological Process	GO:0009566	fertilization	3.36	0.045116925	*Vdac2/Cct5/Aldoa*
Biological Process	GO:0008088	axo-dendritic transport	3.36	0.008691965	*Bsn/Nefm/Map1a/Hspa8/Mapt*
Biological Process	GO:0097164	ammonium ion metabolic process	3.36	0.045116925	*Snca/Mecp2/Sncb*
Biological Process	GO:0008344	adult locomotory behavior	3.36	0.008691965	*Mecp2/Dnm1/Cnp/Mapt/Snca*
Biological Process	GO:0045807	positive regulation of endocytosis	3.14	0.002853243	*Ap2b1/Amph/Sh3gl1/Hspa8/Hnrnpk/Dnm1/Snca*
Biological Process	GO:0033044	regulation of chromosome organization	3.1	0.006347616	*Snca/Mecp2/Cct5/Ube2n/Mapt/Hnrnpc*

Biological Process	GO:0031113	regulation of microtubule polymerization	2.99	0.031447633	*Mecp2/Map2/Mapt/Snca*
Biological Process	GO:0021987	cerebral cortex development	2.99	0.031447633	*Mecp2/Atp1a3/Hnrnpk/Nefm*
Biological Process	GO:0007017	microtubule-based process	2.82	0.000131342	*Mecp2/Map1a/Tppp3/Tppp/Npm1/Tubb5/Tubb4b/Hspa8/Map2/Cnp/Mapt/Nefm/Bsn*
Biological Process	GO:0048639	positive regulation of developmental growth	2.8	0.021424848	*Rufy3/Hnrnpk/Mapt/Ddx39b/Mecp2*
Biological Process	GO:0032886	regulation of microtubule-based process	2.69	0.004540915	*Hdac6/Mecp2/Map1a/Smc3/Npm1/Map2/Mapt/Snca*
Biological Process	GO:2000278	regulation of DNA biosynthetic process	2.69	0.046618079	*Hnrnpc/Ddx39b/Npm1/Cct5*
Biological Process	GO:0043112	receptor metabolic process	2.69	0.046618079	*Ap2b1/Snap25/Dnm1/Snca*
Biological Process	GO:0045786	negative regulation of cell cycle	2.69	0.046618079	*Smc3/Npm1/Cbx5/Npm1*
Biological Process	GO:0010001	glial cell differentiation	2.69	0.046618079	*Mecp2/Cnp/Mapt/Gap4*
Biological Process	GO:0051054	positive regulation of DNA metabolic process	2.59	0.030848118	*Ube2n/Mecp2/Ddx39b/Npm1/Cct5*
Biological Process	GO:0051494	negative regulation of cytoskeleton organization	2.59	0.030848118	*Hdac6/Map1a/Npm1/Map2/Snca*
Biological Process	GO:0007612	learning	2.59	0.030848118	*Amph/Vdac3/Snap25/Map1a/Mecp2*
Biological Process	GO:0030534	adult behavior	2.59	0.030848118	*Mecp2/Dnm1/Cnp/Mapt/Snca*
Biological Process	GO:0040007	growth	2.52	0.020443764	*Hdac6/Eif4h/Cyfip2/Pkm/Mapt/Gap43*
Biological Process	GO:0048589	developmental growth	2.52	0.020443764	*Hdac6/Eif4h/Cyfip2/Pkm/Mapt/Gap43*
Biological Process	GO:0030705	cytoskeleton-dependent intracellular	2.52	0.020443764	*Nefm/Bsn/Mapt/Map1a/Tubb5/Hspa8*

		transport			
Biological Process	GO:0051052	regulation of DNA metabolic process	2.48	0.013546516	*Ddx39b/Smc3/Npm1/Cct5/Ube2n/Hnrnpc/Mecp2*
Biological Process	GO:0010970	transport along microtubule	2.4	0.042533368	*Bsn/Nefm/Mapt/Map1a/Hspa8*
Biological Process	GO:0031647	regulation of protein stability	2.4	0.042533368	*Hdac6/Npm1/Cct5/Hspa8/Snca*
Biological Process	GO:0045927	positive regulation of growth	2.4	0.042533368	*Mecp2/Ddx39b/Rufy3/Hnrnpk/Mapt*
Biological Process	GO:0030307	positive regulation of cell growth	2.4	0.042533368	*Mecp2/Ddx39b/Rufy3/Hnrnpk/Mapt*
Biological Process	GO:0099111	microtubule-based transport	2.4	0.042533368	*Map1a/Hspa8/Mapt/Bsn/Nefm*
Biological Process	GO:0050808	synapse organization	2.37	0.027933148	*Hdac6/Mecp2/Sncb/Mapt/Bsn/Snca*
Biological Process	GO:0007409	axonogenesis	2.35	0.018395686	*Mapt/Gap43/Cnp/Map2/Hdac6/Sptbn1/Snap25/Map2*
Biological Process	GO:1902903	regulation of supramolecular fiber organization	2.34	0.012118553	*Hdac6/Mecp2/Dstn/Map1a/Hspa8/Map2/Mapt/Snca*
**Biological Process**	**GO:1901214**	**regulation of neuron death**	**2.24**	**0.037052663**	***Npm1/Mapt/Snca/Mecp2/Ap2b1/Sncb***
Biological Process	GO:0080135	regulation of cellular response to stress	2.24	0.024359551	*Hdac6/Ddx39b/Npm1/Hspa8/Hnrnpk/Mapt/Ube2n*
Biological Process	GO:1902679	negative regulation of RNA biosynthetic process	2.24	0.010540288	*Hdac6/Mecp2/Cbx5/Ywhaq/Hspa8/Hnrnpk/Hdgf/Cbx3/Snca*
Biological Process	GO:0007611	learning or memory	2.24	0.037052663	*Mecp2/Map1a/Amph/Snap25/Mapt/Vdac3*
Biological Process	GO:0050890	cognition	2.24	0.037052663	*Mecp2/Map1a/Vdac3/Snap25/Mapt/Amph*
Biological Process	GO:0061564	axon development	2.24	0.016030796	*Hdac6/Sptbn1/Snap25/Map2/Cnp/Mapt/Nefm/Gap43*
Biological Process	GO:0030100	regulation of endocytosis	2.14	0.031544861	*Snca/Ap2b1/Amph/Sh3gl1/Snca/Dnm1/Hspa8/Hnrnpk*
Biological Process	GO:0051253	negative regulation of RNA metabolic	2.11	0.01033928	*Hdac6/Mecp2/Hnrnpc/Npm1/Cbx5/Ywhaq/Hspa8/Hnrnpk/Hdgf/Cbx3/Snca*

		process			
Biological Process	GO:0006897	endocytosis	2.07	0.007663286	*Amph/Sh3gl1/Cyfip2/Hspa8/Actg1/Snap25/Cap1/Dnm1/Ncl/Ap2b1/Snca/Alb*
Biological Process	GO:0009890	negative regulation of biosynthetic process	1.9	0.013829442	*Snca/Hdac6/Mecp2/Hnrnpc/Smc3/Cbx5/Ywhaq/Hspa8/Hnrnpk/Hdgf/Cbx3/Ncl/Atp1a1*
Biological Process	GO:0007010	cytoskeleton organization	1.9	0.00313348	*Npm1/Tubb5/Csrp1/Actg1/Cap1/Map2/Cnp/Mapt/Nefm/Bsn/Sptbn1/Tppp/Tppp3/Rufy3/Mecp2/Dstn/Map1a*
Biological Process	GO:0080134	regulation of response to stress	1.89	0.038255668	*Hdac6/Ddx39b/Pcbp2/Npm1/Hspa8/Hnrnpk/Ube2n/Mapt/Snca*
Biological Process	GO:0051493	regulation of cytoskeleton organization	1.89	0.038255668	*Snca/Mapt/Hdac6/Mecp2/Dstn/Map1a/Smc3/Npm1/Map2*
Biological Process	GO:0098657	import into cell	1.86	0.01541032	*Ap2b1/Atp1a1/Amph/Sh3gl1/Cyfip2/Hspa8/Actg1/Snap25/Cap1/Dnm1/Ncl/Alb/Snca*
Biological Process	GO:0022414	reproductive process	1.82	0.048941804	*Eif4h/Smc3/Vdac2/Cct5/Hspa8/Hnrnpk/Mapt/Ncl/Aldoa/Hsd17b10*
Biological Process	GO:0000003	reproduction	1.82	0.048941804	*Mapt/Ncl/Aldoa/Hsd17b10/Hnrnpk/Hspa8/Cct5/Eif4h/Smc3/Vdac2*
Biological Process	GO:0051726	regulation of cell cycle	1.82	0.048941804	*Mecp2/Smc3/Mapre3/Npm1/Cbx5/Tubb5/Tubb4b/Hspa8/Cbx3/Ddx39b*
Biological Process	GO:0006355	regulation of transcription, DNA-templated	1.71	0.025168494	*Ncl/Snca/Cbx3/Ube2n/Hdgf/Hdac6/Mecp2/Ddx39b/Mapre3/Npm1/Cbx5/Ywhaq/Hspa8/Hnrnpk*
Biological Process	GO:2001141	regulation of RNA biosynthetic process	1.65	0.043395938	*Ncl/Snca/Cbx3/Hdgf/Ube2n/Hdac6/Mecp2/Ddx39b/Mapre3/Npm1/Cbx5/Ywhaq/Hspa8/Hnrnpk*
Biological Process	GO:0051252	regulation of RNA metabolic process	1.61	0.038140236	*Hdac6/Mecp2/Hnrnpc/Ddx39b/Mapre3/Ahcyl1/Npm1/Hspa8/Ywhaq/Cbx5/Hnrnpk/Ube2n/Hdgf/Cbx3/Ncl/Snca*

Biological Process	GO:0030030	cell projection organization	1.6	0.036476739	*Ncdn/Map1a/Atp1a3/Sptbn1/Cyfip2/Tubb5/Tubb4b/Snap25/Map2/Mecp2/Cnp/Mapt/Nefm/Gap43/Hdac6*
Biological Process	GO:0031326	regulation of cellular biosynthetic process	1.54	0.026651994	*Cbx3/Ncl/Snca/Hdgf/Ube2n/Hspa8/Hnrnpk/Mecp2/Hnrnpc/Ddx39b/Eif4h/Smc3/Atp1a1/Mapre3/Cbx5/Cct5/Ywhaq/Hdac6/Npm1*
Biological Process	GO:0010556	regulation of macromolecule biosynthetic process	1.51	0.038289945	*Hdac6/Mecp2/Hnrnpc/Ddx39b/Eif4h/Smc3/Mapre3/Npm1/Cbx5/Cct5/Ywhaq/Hspa8/Hnrnpk/Ube2n/Hdgf/Cbx3/Ncl/Snca*
Biological Process	GO:0051128	regulation of cellular component organization	1.4	0.036590734	*Hdac6/Mecp2/Hnrnpc/Ddx39b/Dstn/Map1a/Ap2b1/Rufy3/Smc3/Tppp/Amph/Npm1/Sh3gl1/Vdac2/Cct5/Hspa8/Ube2n/Snap25/Dnm1/Map2/Hnrnpk/Mapt/Snca/Gap43/Nefm/Aldoa*
Molecular Function	GO:0043024	ribosomal small subunit binding	6.78	0.021423125	*Eif4h/Npm1*
Molecular Function	GO:1990829	C-rich single-stranded DNA binding	6.78	0.021423125	*Pcbp2/Hnrnpk*
Molecular Function	GO:0048027	mRNA 5'-UTR binding	6.78	0.021423125	*Cct5/Ncl*
Molecular Function	GO:0001222	transcription corepressor binding	6.78	0.021423125	*Hdgf/Bsn*
Molecular Function	GO:0050542	icosanoid binding	6.78	0.021423125	*Hspa8/Snca*
Molecular Function	GO:0022839	ion gated channel activity	5.09	0.010973427	*Vdac3/Vdac2/Snap25*
Molecular Function	GO:0022836	gated channel activity	5.09	0.010973427	*Vdac3/Vdac2/Snap25*
Molecular Function	GO:0005244	voltage-gated ion channel activity	5.09	0.010973427	*Vdac3/Vdac2/Snap25*
Molecular Function	GO:0022832	voltage-gated channel activity	5.09	0.010973427	*Vdac3/Vdac2/Snap25*
Molecular Function	GO:0005507	copper ion binding	4.52	0.005131633	*Atox1/Snca/Sncb/Alb*

Molecular Function	GO:0048487	beta-tubulin binding	4.24	0.002344101	*Snca/Cct5/Hdac6/Smc3/Sncb*
Molecular Function	GO:0047485	protein N-terminus binding	4.24	0.002344101	*Mecp2/Npm1/Ywhaq/Snap25/Snca*
Molecular Function	GO:0043014	alpha-tubulin binding	4.07	0.024548001	*Hdac6/Snca/Sncb*
Molecular Function	GO:0070840	dynein complex binding	4.07	0.024548001	*Hdac6/Smc3/Snca*
Molecular Function	GO:0042562	hormone binding	4.07	0.024548001	*Atp1a1/Atp1a3/Pkm*
Molecular Function	GO:0015079	potassium ion transmembrane transporter activity	4.07	0.024548001	*Atp1a1/Atp1a3/Snap25*
Molecular Function	GO:0005504	fatty acid binding	4.07	0.024548001	*Snca/Hspa8/Alb*
Molecular Function	GO:0005216	ion channel activity	4.07	0.024548001	*Vdac3/Vdac2/Snap25*
Molecular Function	GO:0033293	monocarboxylic acid binding	4.07	0.024548001	*Hspa8/Alb/Snca*
Molecular Function	GO:0043531	ADP binding	3.39	0.043964369	*Atp1a1/Hspa8/Pkm*
Molecular Function	GO:0005496	steroid binding	3.39	0.043964369	*Atp1a1/Atp1a3/Hsd17b10*
Molecular Function	GO:0008017	microtubule binding	3.05	0.000830018	*Nefm/Snca/Mapt/Map2/Hdac6/Map1a/Tppp/Mapre3/Dnm1*
Molecular Function	GO:0042393	histone binding	3.01	0.03038521	*Npm1/Cbx5/Ncl/Snca*
Molecular Function	GO:0015631	tubulin binding	2.94	7.34771E-05	*Dnm1/Map2/Mapt/Nefm/Snca/Cct5/Mapre3/Tppp/Sncb/Map1a/Smc3/Tppp3/Hdac6*
Molecular Function	GO:0001067	regulatory region nucleic acid binding	2.91	0.009289889	*Npm1/Hnrnpk/Hdgf/Cbx3/Snca/Hdac6*
Molecular Function	GO:0043021	ribonucleoprotein complex binding	2.83	0.020515093	*Hnrnpc/Eif4h/Npm1/Hnrnpk/Cbx5*
Molecular Function	GO:0043565	sequence-specific DNA binding	2.79	0.006288047	*Hdac6/Mecp2/Hnrnpk/Hdgf/Cbx3/Mapt/Ncl*
Molecular Function	GO:0008270	zinc ion binding	2.71	0.045113098	*Snca/Alb/Crip2/Csrp1*
Molecular Function	GO:0051082	unfolded protein binding	2.71	0.045113098	*Npm1/Cct5/Tubb4b/Hspa8*

Molecular Function	GO:1990837	sequence-specific double-stranded DNA binding	2.61	0.029589221	*Hdac6/Hnrnpk/Hdgf/Cbx3/Mapt*
Molecular Function	GO:0060090	binding, bridging	2.61	0.029589221	*Map1a/Lasp1/Cbx5/Hspa8/Mapt*
Molecular Function	GO:0030674	protein binding, bridging	2.61	0.029589221	*Map1a/Cbx5/Hspa8/Mapt/Lasp1*
Molecular Function	GO:0003690	double-stranded DNA binding	2.54	0.019424592	*Mapt/Hdac6/Mecp2/Hnrnpk/Hdgf/Cbx3*
Molecular Function	GO:0046873	metal ion transmembrane transporter activity	2.42	0.040866628	*Atox1/Slc6a11/Atp1a3/Atp1a1/Atp1a3/Snap25*
Molecular Function	GO:0046914	transition metal ion binding	2.14	0.04576356	*Snca/Atox1/Alb/Sncb/Csrp1/Alb/Crip2*
Molecular Function	GO:0003677	DNA binding	2.13	0.009729336	*Snca/Hdgf/Cbx3/Mapt/Ncl/Alb/Hnrnpk/Hdac6/Mecp2/Pcbp2/Npm1*
Molecular Function	GO:0003723	RNA binding	2.02	0.004050422	*Hsd17b10/Ncl/Mapt/Mecp2/Hnrnpc/Ddx39b/Eif4h/Eef1g/Pcbp2/Npm1/Cct5/Tubb4b/Hspa8/Hnrnpk*
Molecular Function	GO:0008134	transcription factor binding	1.94	0.04834994	*Mecp2/Npm1/Cbx5/Hspa8/Hnrnpk/Hdgf/Bsn/Hsd17b10*
Molecular Function	GO:0003676	nucleic acid binding	1.9	0.002162861	*Hdac6/Mecp2/Ddx39b/Alb/Snca/Hsd17b10/Ncl/Eif4h/Eef1g/Pcbp2/Npm1/Cct5/Hnrnpc/Tubb4b/Hnrnpk/Hdgf/Cbx3/Mapt/Hspa8*
Molecular Function	GO:0008092	cytoskeletal protein binding	1.87	0.000727309	*Atp1a1/Tppp/Mapre3/Sptbn1/Lasp1/Crmp1/Cct5/Hnrnpk/Snap25/Cap1/Dnm1/Map2/Mapt/Sncb/Hdac6/Dstn/Map1a/Smc3/Tppp3/Nefm/Aldoa/Snca*

Fold Enrichment ＞1.30

p value ＜0.05

Table S4

GO enrichment analysis for BP of downregulated MeCP2 binding peaks in the penumbra from 2-DG or 4-CIN treatment group, related to Figure3H

TermID	TermName	MCAO+2-DG vs. MCAO+Saine			MCAO+4-CIN vs. MCAO+Saine		
		Enrichment	FDR	Genes	Enrichment	FDR	Genes
GO:0098989	NMDA selective glutamate receptor signaling pathway	13.5214805	0.014	*Kalrn/Tiam1/Camk2a*	21.200	0.0085	*Kalrn/Tiam1/Camk2a*
GO:0045838	positive regulation of membrane potential	4.30228925	0.0214	*Akt2/Ndufc2/Ank3/Gria1/Tcl1/Akt1/Slc34a1*	5.782	0.0242	*Akt2/Tmem135/Ndufc2/Gria1/Tcl1/Bad*
GO:0035418	protein localization to synapse	2.7042961	0.0225	*Cacng3/Mpp4/Asic2/Gphn/Kalrn/Dlgap1/Camk2a/Nrxn2/Kif5c/Map1a/Mapk10/Lrrtm1/Grip2*	3.588	0.0144	*Grip2/Kif5a/Mpp4/Asic2/Nptx1/Homer1/Kalrn/Dlgap1/Camk2a/Map1a/Mapk10*
GO:0007613	memory	2.50054776	0.0007	*Atp1a3/Cic/Kmt2b/Abcc8/Eif4ebp2/Igf2/Shank2/Crtc1/Gria1/Psen1/Lgmn/Pla2g6/Asic1/Ntan1/Kalrn/Btbd9/Rcan2/Srf/Adrb1/Eif2ak4/Map1a/Chrnb2/Cnr1/Foxo6/Mtor/Cux2/Hrh1*	2.904	0.0025	*Cux2/Hrh1/Abcc8/Jph3/Cck/Gria1/Hrh2/Nfatc4/Pla2g6/Kalrn/Rcan2/Adrb1/Map1a/Kcnk2/Chrnb2/Cnr1/Musk/Foxo6/Mtor*
GO:1902414	protein localization to cell junction	2.43082796	0.0214	*Cacng3/Mpp4/Asic2/Gphn/Mapk8/Heg1/Kalrn/Dlgap1/Camk2a/Nrxn2/Kif5c/Map1a/Mapk10/Actb/Lrrtm1/Grip2*	3.573	0.0023	*Actb/Grip2/Kif5a/Mpp4/Asic2/Nptx1/Homer1/Mapk8/Heg1/Kalrn/Nectin3/Dlgap1/Camk2a/Map1a/Mapk10*
GO:0097485	neuron projection guidance	2.25358008	0.0007	*Rhog/Mypn/Ank3/Dpysl4/Palld/Pou4f2/Ryk/Tmeff2/Slit3/Ngfr/Wnt3/Bcl11b/Unc5a/Wnt5a/Gli2/Gdnf/Ephb3/Lhx4/Sema6a/Dcc/Slit1/Ablim1/Vax1/Kif5c/Bmp7/Cdh4/Efna1/Bmpr1b/Runx3/Ephb2/Epha2/Draxin/Plxna4*	2.141	0.0472	*Plxna4/Mypn/Dpysl4/Pou4f2/Tmeff2/Kif5a/Epha4/Gdnf/Lhx4/Sema6a/Ablim1/Vax1/Bmp7/Cdh4/Efna1/Sema6c/Bmpr1b/Runx3/Epha2/Draxin*
GO:1901216	positive regulation of neuron death	2.23398374	0.0214	*Ube2m/Grik5/Gsk3a/Map2k7/Egln1/Pin1/Elk1/Ngfr/Fos/Wnt5a/Mapk8/Atf4/Daxx/Ralbp1/Ptprf/Casp9/Mtor/Map3k5/Foxo3*	2.581	0.0443	*Nos1/Foxo3/Grik5/Bax/Egln1/Pin1/Mapk8/Pmaip1/Map3k11/Bad/Abl1/Mcl1/Musk/Mtor*
GO:0043087	regulation of GTPase activity	2.11473392	1E-06	*Akt2/Ankrd27/Rhog/Dock1/Ccl25/Gipc1/Cx3cl1/Ccl17/Pin1/Arhgef12/Gnb5/Nprl2/Tbc1d30/Arhgap9/Tbc1d10a/Arhgef15/Rap1gap2/Pafah1b1/Abr/Ccl1/Bcas3/Ccr7/Arhgdia/Asap2/Gdi2/Iqgap2/Depdc1b/Snx18/Wnt5a/Ptk2b/Asap1/Arhgap8/Rapgef3/Racgap1/Ephb3/Kalrn/Stxbp5l/Arhgap31/Tiam1/Rgs8/Ralbp1/Arhgap12/Arap3/Arhgap26/Tbc1d10c/Rasgrp2/Adrb1/Gpsm1/Dab2ip/Arhgap15/Itga6/Pip5k1a/Rap1gds1/Wnt4/Rap1gap/Rcc2/Epha2/Mtor/Arap2/Arhgap24/Rasal1/Stard13/Plxna4/Dgki/Iqsec1/Plxnd1*	1.859	0.0151	*Rasal1/Plxna4/Dgki/Chn2/Akt2/Ccl25/Cx3cl1/Pin1/Gnb5/Srgap1/Arhgef15/Ccl1/Ccr7/Rgs9/Arhgdia/Epha4/Pde6d/Gdi2/Iqgap2/Depdc1b/Asap1/Arhgap8/Racgap1/Kalrn/Tiam1/Mlst8/Arap3/Rasgrp2/Ric1/Adrb1/Gpsm1/Arhgap15/Rcc2/Epha2/Mtor/Arap2/Arhgap24*
GO:0010970	transport along microtubule	2.06578174	0.0238	*Pcm1/Tmem108/Ttc21a/Fyco1/Pafah1b1/Ap3b1/Ap3m1/Kifc1/Pura/Kif5c/Ttc30a1/Kif28/Map1a/Ift80/Wdr19/Dynll1/Bicdl1/Ift81/Sun1/Dync1i1/Wasf1/Arl8b*	2.503	0.0259	*Wasf1/Ift122/Ap3b2/Pcm1/Tmem108/Fyco1/Kif5a/Agtpbp1/Ap3b1/Kifc1/Pura/Kif28/Ttc30a1/Map1a/Spg11/Ift80/Prkcz/Wasf1/Ift122/Ap3b2/D430042O09Rik/Pcm1/Tmem108/Fyco1/Kif5a/Agtpbp1/Ap3b1/Lrrc6/Kifc1/Pura/Kif28/Ttc30a1/Map1a/Spg11/Ift80/Dnaic1/Prkcz*
GO:0048167	regulation of synaptic plasticity	1.99824835	0.0091	*Brsk1/Eif4ebp2/Stx4a/Shank2/Crtc1/Gipc1/Slc24a1/Gria1/Rara/Psen1/Lgmn/Ptk2b/Rims2/Adgrb1/Srf/Lrrtm2/Synpo/Camk2a/Syt7/Adrb1/Eif2ak4/Map1a/Kcnb1/Ephb2/Htr6/Ywhag/Vgf/Dgki/Lrrtm1/Hrh1*	2.193	0.0336	*Nos1/Grid2ip/Dgki/Hrh1/Brsk1/Jph3/Gria1/Epha4/Hrh2/Nfatc4/Shisa9/Kcnn2/Camk2a/Syt7/Adrb1/Abl1/Map1a/Spg11/Mmp9/Musk/Prkcz*

GO:0099111	microtubule-based transport	1.98845302	0.0213	*D430042O09Rik/Pcm1/Tmem108/Ttc21a/Fyco1/Pafah1b1/Kif27/Ap3b1/Ap3m1/Lrrc6/Kifc1/Pura/Kif5c/Ttc30a1/Kif28/Map1a/Ift80/Wdr19/Dynll1/Bicdl1/Ift81/Sun1/Dync1i1/Wasf1/Arl8b*	2.494	0.0113	*Wasf1/Ift122/Ap3b2/D430042O09Rik/Pcm1/Tmem108/Fyco1/Kif5a/Agtpbp1/Ap3b1/Lrrc6/Kifc1/Pura/Kif28/Ttc30a1/Map1a/Spg11/Ift80/Dnaic1/Prkcz*
GO:0050804	modulation of chemical synaptic transmission	1.98605247	3E-06	*Brsk1/Calm3/Grik5/Apba2/Eif4ebp2/Syt9/Stx4a/Shank2/Crtc1/Gipc1/Cacna1a/Egln1/Grik4/Slc24a1/Nedd4/Tmem108/Gria1/Abr/Pnkd/Ngfr/Rara/Psen1/Lgmn/Nrn1/Ror2/Htr1a/Ptk2b/Ptger4/Gdnf/Rims2/Adgrb1/Pla2g6/Asic1/Gm28040/Stxbp5l/Btbd9/Srf/Plcl2/Sh3gl1/Prkce/Lrrtm2/Synpo/Camk2a/Adrb2/Dcc/Syt7/Apba1/Adrb1/Ntng2/Dgkz/Eif2ak4/Map1a/Kcnb1/Chrnb2/Ppp3ca/Eif4e/Cnr1/Kcnq4/Ephb2/Htr6/Mtor/Uts2/Cux2/Ywhag/Vgf/Prkar1b/Lrrc4/Dgki/Lrrtm1/Slc6a6/Grip2/Hrh1/Clstn3*	2.133	0.0001	*Nos1/Cux2/Prkar1b/Grid2ip/Dgki/Grip2/Hrh1/Clstn3/Vamp1/Brsk1/Grik5/Apba2/Syt9/Jph3/Egln1/Tmem108/Gria1/Pnkd/Nptx1/Epha4/Begain/Nrn1/Hrh2/Homer1/Nfatc4/Ptger4/Gdnf/Pla2g6/Shisa9/Plcl2/Sh3gl1/Kcnn2/Sncaip/Camk2a/Syt7/Adrb1/Ntng2/Abl1/Dgkz/Map1a/Spg11/Plcb4/Mmp9/Chrnb2/Eif4e/Cnr1/Musk/Mtor/Uts2/Prkcz*
GO:0099177	regulation of trans-synaptic signaling	1.98206441	3E-06	*Brsk1/Calm3/Grik5/Apba2/Eif4ebp2/Syt9/Stx4a/Shank2/Crtc1/Gipc1/Cacna1a/Egln1/Grik4/Slc24a1/Nedd4/Tmem108/Gria1/Abr/Pnkd/Ngfr/Rara/Psen1/Lgmn/Nrn1/Ror2/Htr1a/Ptk2b/Ptger4/Gdnf/Rims2/Adgrb1/Pla2g6/Asic1/Gm28040/Stxbp5l/Btbd9/Srf/Plcl2/Sh3gl1/Prkce/Lrrtm2/Synpo/Camk2a/Adrb2/Dcc/Syt7/Apba1/Adrb1/Ntng2/Dgkz/Eif2ak4/Map1a/Kcnb1/Chrnb2/Ppp3ca/Eif4e/Cnr1/Kcnq4/Ephb2/Htr6/Mtor/Uts2/Cux2/Ywhag/Vgf/Prkar1b/Lrrc4/Dgki/Lrrtm1/Slc6a6/Grip2/Hrh1/Clstn3*	2.129	0.0001	*Nos1/Cux2/Prkar1b/Grid2ip/Dgki/Grip2/Hrh1/Clstn3/Vamp1/Brsk1/Grik5/Apba2/Syt9/Jph3/Egln1/Tmem108/Gria1/Pnkd/Nptx1/Epha4/Begain/Nrn1/Hrh2/Homer1/Nfatc4/Ptger4/Gdnf/Pla2g6/Shisa9/Plcl2/Sh3gl1/Kcnn2/Sncaip/Camk2a/Syt7/Adrb1/Ntng2/Abl1/Dgkz/Map1a/Spg11/Plcb4/Mmp9/Chrnb2/Eif4e/Cnr1/Musk/Mtor/Uts2/Prkcz*
GO:0045665	negative regulation of neuron differentiation	1.96370049	0.005	*Gsk3a/Pou4f2/Ryk/Amigo3/Pmp22/Rap1gap2/Pafah1b1/Ngfr/Wnt3/Id2/Psen1/Ptprg/Wnt5a/Actr3/Asap1/Notch3/Sema6b/Sema6a/Slit1/Dlx2/Eif2ak4/Jag1/Bmp7/Efna1/Ppp3ca/Eif4e/Dab1/Ephb2/Rap1gap/Draxin/Disp3/Hes5/Rbpj/Phox2b/Foxo3*	2.199	0.0137	*Trpv4/Foxo3/Dguok/Cib1/Pou4f2/Sema7a/Epha4/Mylip/Spock1/Ptprg/Nfatc4/Asap1/Notch3/Sema6a/Jag1/Bmp7/Efna1/Sema6c/Eif4e/Dab1/Draxin/Casz1/Rbpj/Phox2b/Hdac2*
GO:0045666	positive regulation of neuron differentiation	1.923055	5E-05	*Ankrd27/Gdpd5/Shank2/Crtc1/Pou4f2/Cx3cl1/Trim67/Tle6/Nck1/Twf2/Actr2/Pafah1b1/Mmd/Spag9/Ngfr/Rara/Wnt3/Obsl1/Nin/Psen1/Rims1/Ror2/Arsb/Rarb/Wnt5a/Gli2/Ptk2b/Stk24/Actr3/Rims2/Etv5/Syt2/Kalrn/Tiam1/Itsn1/Qk/Srf/Tgif1/Fbxo38/Tcf4/Dcc/Vldlr/Camk1d/Dab2ip/Dlx2/Itga6/Nfe2l2/Bmp7/Cdh4/Cnr1/Trim32/Dab1/Ptprf/Foxo6/Mtor/Adra2c/Phox2b/Rasal1/Cux2/Ocm/Plxna4/Fig4/Grip2/Plxnd1*	1.743	0.037	*Rasal1/Cux2/Plxna4/Grip2/Fes/Gdpd5/Pou4f2/Cx3cl1/Sema7a/Llph/Mmd/Nme1/Spag9/Rnf157/Epha4/Rims1/Rarb/Stk24/Syt2/Kalrn/Adamts1/Tiam1/Itsn1/Qk/Vldlr/Nfe2l2/Bmp7/Cdh4/Ss18l1/Cnr1/Musk/Trim32/Dab1/Foxo6/Mtor/Adra2c/Phox2b*
GO:0030705	cytoskeleton-dependent intracellular transport	1.90793897	0.0434	*Pcm1/Tmem108/Ttc21a/Fyco1/Pafah1b1/Ap3b1/Ap3m1/Myo10/Kifc1/Pura/Kif5c/Ttc30a1/Kif28/Map1a/Ift80/Wdr19/Dynll1/Bicdl1/Ift81/Sun1/Dync1i1/Wasf1/Arl8b*	2.471	0.0163	*Wasf1/Wasl/Ift122/Ap3b2/Pcm1/Tmem108/Fyco1/Kif5a/Agtpbp1/Ap3b1/Myo10/Kifc1/Pura/Kif28/Ttc30a1/Map1a/Spg11/Ift80/Prkcz*

GO:0045664	regulation of neuron differentiation	1.87697101	1E-07	*Sox5/Brsk1/Gsk3a/Ankrd27/Gdpd5/Shank2/Crtc1/Pou4f2/Cacna1a/Cx3cl1/Trim67/S1pr5/Tle6/Nedd4/Nck1/Ryk/Twf2/Amigo3/Actr2/Abi2/Pmp22/Rap1gap2/Pafah1b1/Mmd/Spag9/Ngfr/Rara/Wnt3/Obsl1/Id2/Nin/Psen1/Bcl11b/Rims1/Akt1/Ror2/Arsb/Ptprg/Rarb/Zswim8/Wnt5a/Mapk8/Gli2/Ptk2b/Stk24/Actr3/Rims2/Asap1/Ephb3/Etv5/Syt2/Kalrn/Tiam1/Itsn1/Qk/Notch3/Srf/Sema6b/Tgif1/Sema6a/Fbxo38/Tcf4/Dcc/Vldlr/Slit1/Camk1d/Olfm1/Dab2ip/Dlx2/Itga6/Nfe2l2/Eif2ak4/Jag1/Bmp7/Cdh4/Sfrp2/Efna1/Chrnb2/Ppp3ca/Eif4e/Cnr1/Trim32/Dab1/Ptprf/Foxo6/Ephb2/Rap1gap/Draxin/Disp3/Mtor/Hes5/Adra2c/Rbpj/Phox2b/Rasal1/Cux2/Ywhag/Ocm/Plxna4/Fig4/Grip2/Foxo3/Plxnd1*	1.771	0.0016	*Trpv4/Rasal1/Cux2/Foxo3/Plxna4/Dguok/Grip2/Brsk1/Cib1/Fes/Gdpd5/Pou4f2/Cx3cl1/S1pr5/Sema7a/Llph/Mmd/Nme1/Spag9/Rnf157/Epha4/Rims1/Mylip/Spock1/Ptprg/Rarb/Mapk8/Nfatc4/Rap2a/Stk24/Asap1/Syt2/Kalrn/Adamts1/Tiam1/Itsn1/Qk/Notch3/Sema6a/Vldlr/Abl1/Nfe2l2/Jag1/Bmp7/Cdh4/Ss18l1/Efna1/Chrnb2/Sema6c/Eif4e/Cnr1/Musk/Trim32/Dab1/Foxo6/Draxin/Mtor/Casz1/Adra2c/Rbpj/Phox2b/Hdac2*
GO:0051345	positive regulation of hydrolase activity	1.86366447	9E-07	*Calm3/Akt2/Ankrd27/Pdcd5/Nr1h2/Rhog/Dock1/Ccl25/Gipc1/Cx3cl1/Ccl17/Pin1/Arhgef12/Apoa1/Slc39a10/Smad3/Gnb5/Nprl2/Tbc1d30/Arhgap9/Tbc1d10a/Nmur2/Arhgef15/Rap1gap2/Abr/Ccl1/Bcas3/Ngfr/Ccr7/Myl4/Arhgdia/Asap2/Lgmn/Akt1/Gdi2/Syk/Depdc1b/Snx18/Wnt5a/Mapk8/Sox7/Ptk2b/Dap/Asap1/Gpihbp1/Arhgap8/Gramd4/Rapgef3/Racgap1/Tfap4/Kalrn/Stxbp5l/Arhgap31/Tiam1/Tnnt2/BC004004/Rgs8/Ralbp1/Msh2/Arhgap12/Arap3/Arhgap26/Ppargc1b/Adrb2/Malt1/Tbc1d10c/Rasgrp2/Adrb1/Dab2ip/Arhgap15/Itga6/Adrm1/Sfrp2/Efna1/Pip5k1a/Rap1gds1/Bcl10/Casp8ap2/Acer2/Wnt4/Rap1gap/Rcc2/Epha2/Casp9/Arap2/Txk/Arhgap24/Map3k5/Dynll1/Rasal1/Stard13/Dync1i1/Adcyap1r1/Iqsec1*	1.585	0.0375	*Rasal1/Chn2/Adcyap1r1/Pparg/Akt2/Pdcd5/Bax/Ccl25/Cx3cl1/Pin1/Smad3/Gnb5/Apaf1/Cck/Srgap1/Arhgef15/Ccl1/Ccr7/Rgs9/Arhgdia/Epha4/Gdi2/Syk/Depdc1b/Mapk8/Dap/Asap1/Arhgap8/Racgap1/Kalrn/Tiam1/Lmf1/Arap3/Ppargc1b/Malt1/Pmaip1/Rasgrp2/Bad/Ric1/Adrb1/Arhgap15/Adrm1/Efna1/Casp8ap2/Acer2/Rcc2/Epha2/Prkcz/Arap2/Txk/Arhgap24*
GO:0051271	negative regulation of cellular component movement	1.83194252	0.0052	*Abcc8/Ccl25/Angpt2/Bst2/Cx3cl1/Pin1/Ryk/Tmeff2/Abr/Igfbp5/Wnt3/Arhgdia/Rhob/Actn1/Akt1/Ptprg/Wnt5a/Clasp1/Rgcc/Stk24/Ptger4/Adgrb1/Card10/Srf/Sema6b/Sema6a/Coro1b/Slit1/Dab2ip/Scai/Nfe2l2/Spred1/Frmd5/Jag1/Ptprt/Sfrp2/Wnt4/Miip/Rbpj/Stard13/Mitf/Foxo3*	2.257	0.0018	*Nos1/Foxo3/Wasl/Mitf/Pparg/Abcc8/Ccl25/Angpt2/Nrg1/Cx3cl1/Pin1/Tmeff2/Sema7a/Srgap1/Igfbp5/Arhgdia/Clasp1/Ptprg/Rap2a/Stk24/Ptger4/Aif1/Sema6a/Scai/Nfe2l2/Dusp10/Frmd5/Jag1/Ptprt/Sema6c/Clic4/Miip/Rbpj*
GO:0050769	positive regulation of neurogenesis	1.80768456	6E-05	*Ankrd27/Gdpd5/Nkx6-2/Shank2/Crtc1/Pou4f2/Cx3cl1/Trim67/Tle6/Zfp609/Nck1/Twf2/Actr2/Pafah1b1/Mmd/Spag9/Ngfr/Rara/Wnt3/Obsl1/Id2/Nin/Psen1/Rims1/Ror2/Arsb/Rarb/Wnt5a/Mapk8/Prmt5/Gli2/Ptk2b/Stk24/Actr3/Rims2/Etv5/Syt2/Kalrn/Tiam1/Itsn1/Qk/Srf/Tgif1/Sema6a/Fbxo38/Tcf4/Dcc/Vldlr/Camk1d/Dab2ip/Dlx2/Itga6/Nfe2l2/Bmp7/Cdh4/Fubp1/Cnr1/Trim32/Dab1/Ptprf/Foxo6/Ephb2/Spen/Tnfrsf1b/Mtor/Smarcd3/Adra2c/Phox2b/Rasal1/Cux2/Ocm/Plxna4/Fig4/Grip2/Plxnd1*	1.814	0.0054	*Rasal1/Cux2/Plxna4/Grip2/Pparg/Fes/Gdpd5/Rassf10/Nkx6-2/Pou4f2/Cx3cl1/Sema7a/Zfp609/Llph/Mmd/Nme1/Spag9/Rnf157/Epha4/Rims1/Rarb/Mapk8/Stk24/Syt2/Kalrn/Adamts1/Tiam1/Itsn1/Qk/Sema6a/Vldlr/Nfe2l2/Bmp7/Cdh4/Ss18l1/Cnr1/Musk/Trim32/Dab1/Dmrta2/Foxo6/Spen/Tnfrsf1b/Mtor/Smarcd3/Adra2c/Phox2b/Hdac2*

GO:0051962	positive regulation of nervous system development	1.79565261	2E-05	*Ankrd27/Gdpd5/Nkx6-2/Shank2/Crtc1/Pou4f2/Cx3cl1/Trim67/Vstm5/Tle6/Zfp609/Nck1/Twf2/Amigo3/Actr2/Pafah1b1/Asic2/Mmd/Spag9/Ngfr/Rara/Wnt3/Obsl1/Id2/Nin/Psen1/Rims1/Ror2/Arsb/Rarb/Wnt5a/Mapk8/Prmt5/Gli2/Ptk2b/Stk24/Actr3/Rims2/Adgrb1/Ephb3/Etv5/Syt2/Kalrn/Tiam1/Itsn1/Qk/Srf/Tgif1/Lrrtm2/Sema6a/Fbxo38/Tcf4/Dcc/Vldlr/Camk1d/Dab2ip/Dlx2/Itga6/Nfe2l2/Bmp7/Cdh4/Fubp1/Cnr1/Trim32/Dab1/Ptprf/Foxo6/Ephb2/Spen/Tnfrsf1b/Mtor/Smarcd3/Adra2c/Phox2b/Rasal1/Cux2/Ocm/Plxna4/Lrrtm1/Fig4/Grip2/Plxnd1/Clstn3*	1.696	0.0137	*Rasal1/Cux2/Plxna4/Grip2/Pparg/Clstn3/Fes/Gdpd5/Rassf10/Nkx6-2/Pou4f2/Cx3cl1/Sema7a/Zfp609/Llph/Asic2/Mmd/Nme1/Spag9/Rnf157/Epha4/Rims1/Rarb/Mapk8/Stk24/Syt2/Kalrn/Adamts1/Tiam1/Itsn1/Qk/Sema6a/Vldlr/Nfe2l2/Bmp7/Cdh4/Ss18l1/Cnr1/Musk/Trim32/Dab1/Dmrta2/Foxo6/Spen/Tnfrsf1b/Mtor/Smarcd3/Adra2c/Phox2b/Hdac2*
GO:0040013	negative regulation of locomotion	1.7941123	0.0086	*Abcc8/Ccl25/Angpt2/Bst2/Cx3cl1/Pin1/Ryk/Tmeff2/Abr/Igfbp5/Wnt3/Arhgdia/Rhob/Akt1/Ptprg/Wnt5a/Clasp1/Rgcc/Stk24/Ptger4/Adgrb1/Card10/Srf/Sema6b/Sema6a/Coro1b/Slit1/Dab2ip/Scai/Nfe2l2/Spred1/Frmd5/Jag1/Ptprt/Sfrp2/Wnt4/Miip/Rbpj/Stard13/Mitf/Foxo3*	2.195	0.0034	*Foxo3/Wasl/Mitf/Pparg/Abcc8/Ccl25/Angpt2/Nrg1/Cx3cl1/Pin1/Tmeff2/Sema7a/Srgap1/Igfbp5/Arhgdia/Clasp1/Ptprg/Rap2a/Stk24/Ptger4/Aif1/Sema6a/Scai/Nfe2l2/Dusp10/Frmd5/Jag1/Ptprt/Sema6c/Clic4/Miip/Rbpj*
GO:0050767	regulation of neurogenesis	1.7869798	1E-07	*Sox5/Brsk1/Gsk3a/Ankrd27/Abcc8/Gdpd5/Arntl/Nkx6-2/Shank2/Pcm1/Crtc1/Pou4f2/Cacna1a/Cx3cl1/Trim67/S1pr5/Tle6/Zfp609/Nedd4/Nck1/Ryk/Twf2/Amigo3/Nf2/Actr2/Abi2/Pmp22/Rap1gap2/Pafah1b1/Mmd/Spag9/Ngfr/Rara/Wnt3/Obsl1/Id2/Nin/Psen1/Bcl11b/Rims1/Akt1/Ror2/Arsb/Ptprg/Rarb/Zswim8/Wnt5a/Mapk8/Prmt5/Gli2/Ptk2b/Stk24/Actr3/Rims2/Asap1/Slc45a3/Ephb3/Etv5/Syt2/Kalrn/Tiam1/Itsn1/Qk/Notch3/Tnfrsf21/Srf/Sema6b/Tgif1/Sema6a/Camk2a/Fbxo38/Tcf4/Dcc/Vldlr/Slit1/Vax1/Camk1d/Olfm1/Dab2ip/Dlx2/Itga6/Nfe2l2/Eif2ak4/Jag1/Bmp7/Cdh4/Igsf10/Sfrp2/Efna1/Chrnb2/Ppp3ca/Eif4e/Fubp1/Cnr1/Trim32/Dab1/Ptprf/Foxo6/Ephb2/Rap1gap/Spen/Tnfrsf1b/Draxin/Disp3/Mtor/Hes5/Ski/Smarcd3/Adra2c/Rbpj/Phox2b/Rasal1/Cux2/Ywhag/Ocm/Plxna4/Fig4/Grip2/Foxo3/Plxnd1*	1.868	3E-05	*Trpv4/Nos1/Rasal1/Cux2/Foxo3/Plxna4/Dguok/Grip2/Pparg/Brsk1/Abcc8/Cib1/Fes/Gdpd5/Rassf10/Nkx6-2/Nrg1/Pcm1/Pou4f2/Cx3cl1/S1pr5/Sema7a/Zfp609/Llph/Nf2/Mmd/Nme1/Spag9/Rnf157/Epha4/Rims1/Mylip/Spock1/Ptprg/Rarb/Mapk8/Nfatc4/Rap2a/Stk24/Gpr183/Asap1/Syt2/Kalrn/Adamts1/Tiam1/Itsn1/Qk/Notch3/Sema6a/Camk2a/Vldlr/Tcf7l2/Vax1/Abl1/Nfe2l2/Dusp10/Jag1/Bmp7/Cdh4/Ss18l1/Efna1/Chrnb2/Sema6c/Eif4e/Cnr1/Musk/Trim32/Dab1/Dmrta2/Foxo6/Spen/Tnfrsf1b/Draxin/Mtor/Casz1/Smarcd3/Adra2c/Rbpj/Phox2b/Hdac2*

GO:0051960	regulation of nervous system development	1.78510186	2E-08	*Sox5/Brsk1/Gsk3a/Lrfn1/Lrfn3/Ankrd27/Abcc8/Gdpd5/Arntl/Nkx6-2/Shank2/Pcm1/Crtc1/Pou4f2/Cacna1a/Cx3cl1/Trim67/Vstm5/S1pr5/Zpr1/Tle6/Zfp609/Nedd4/Nck1/Ryk/Twf2/Amigo3/Nf2/Actr2/Abi2/Pmp22/Arhgef15/Rap1gap2/Pafah1b1/Asic2/Mmd/Spag9/Ngfr/Rara/Wnt3/Obsl1/Id2/Nin/Psen1/Bcl11b/Rims1/Akt1/Ror2/Arsb/Ptprg/Rarb/Zswim8/Wnt5a/Colq/Mapk8/Prmt5/Gli2/Ptk2b/Stk24/Actr3/Rims2/Asap1/Adgrb1/Slc45a3/Ephb3/Etv5/Syt2/Kalrn/Tiam1/Itsn1/Qk/Notch3/Tnfrsf21/Srf/Sema6b/Tgif1/Lrrtm2/Sema6a/Camk2a/Fbxo38/Tcf4/Dcc/Lrfn4/Vldlr/Slit1/Sufu/Vax1/Camk1d/Olfm1/Ntng2/Dab2ip/Dlx2/Itga6/Nfe2l2/Eif2ak4/Jag1/Bmp7/Cdh4/Igsf10/Sfrp2/Efna1/Chrnb2/Ppp3ca/Eif4e/Fubp1/Cnr1/Trim32/Dab1/Ptprf/Foxo6/Ephb2/Rap1gap/Spen/Tnfrsf1b/Draxin/Disp3/Mtor/Hes5/Ski/Smarcd3/Adra2c/Rbpj/Phox2b/Rasal1/Cux2/Ywhag/Ocm/Plxna4/Lrrtm1/Fig4/Grip2/Foxo3/Plxnd1/Clstn3*	1.859	1E-05	*Trpv4/Nos1/Rasal1/Cux2/Foxo3/Plxna4/Dguok/Grip2/Pparg/Ift122/Clstn3/Brsk1/Abcc8/Cib1/Fes/Gdpd5/Rassf10/Nkx6-2/Nrg1/Pcm1/Pou4f2/Cx3cl1/S1pr5/Zpr1/Sema7a/Zfp609/Llph/Nf2/Arhgef15/Asic2/Mmd/Nme1/Spag9/Rnf157/Nptx1/Epha4/Rims1/Mylip/Spock1/Ptprg/Rarb/Colq/Mapk8/Nfatc4/Rap2a/Stk24/Gpr183/Asap1/Syt2/Kalrn/Adamts1/Tiam1/Itsn1/Qk/Notch3/Sema6a/Camk2a/Vldlr/Sufu/Tcf7l2/Vax1/Ntng2/Abl1/Nfe2l2/Dusp10/Jag1/Bmp7/Cdh4/Ss18l1/Efna1/Chrnb2/Sema6c/Eif4e/Cnr1/Musk/Trim32/Dab1/Dmrta2/Foxo6/Spen/Tnfrsf1b/Draxin/Mtor/Casz1/Smarcd3/Adra2c/Rbpj/Phox2b/Hdac2*
GO:1903522	regulation of blood circulation	1.76863247	0.0322	*Abcc9/Calm3/Gsk3a/Sptbn4/Mef2a/Ctnna3/Atp2a1/Tmem38a/Cx3cl1/Atp2b1/Smtn/Srebf1/Asic2/Cacna1g/Myl4/Akt1/Pik3r1/Tnnt2/Adrb2/Dusp5/Adrb1/Nos1ap/Mc3r/Ank2/Edn2/Uts2/Kcnh2/Adra2c/Myl2/Tbxas1/Hrh1*	2.057	0.0396	*Nos1/Myl2/Hrh1/Abcc9/Ctnna3/Atp2a1/Tmem38a/Cx3cl1/Hcn4/Smtn/Srebf1/Asic2/Cacna1g/Gaa/Hrh2/Pik3r1/Nos1ap/Dusp5/Adrb1/Snta1/Edn2/Uts2/Adra2c*
GO:0051961	negative regulation of nervous system development	1.76019273	0.008	*Gsk3a/Abcc8/Nkx6-2/Pcm1/Pou4f2/Ryk/Amigo3/Pmp22/Arhgef15/Rap1gap2/Pafah1b1/Ngfr/Wnt3/Id2/Psen1/Ptprg/Wnt5a/Actr3/Asap1/Notch3/Tnfrsf21/Sema6b/Sema6a/Slit1/Sufu/Vax1/Dlx2/Eif2ak4/Jag1/Bmp7/Efna1/Ppp3ca/Eif4e/Dab1/Ephb2/Rap1gap/Draxin/Disp3/Hes5/Ski/Rbpj/Phox2b/Fig4/Foxo3*	2.133	0.0034	*Trpv4/Foxo3/Dguok/Ift122/Abcc8/Cib1/Nkx6-2/Nrg1/Pcm1/Pou4f2/Sema7a/Arhgef15/Epha4/Mylip/Spock1/Ptprg/Nfatc4/Asap1/Notch3/Sema6a/Sufu/Vax1/Dusp10/Jag1/Bmp7/Efna1/Sema6c/Eif4e/Dab1/Draxin/Casz1/Rbpj/Phox2b/Hdac2*
GO:0001505	regulation of neurotransmitter levels	1.746017	0.0103	*Brsk1/Calm3/Grik5/Akt2/Apba2/Syt9/Gipc1/Cacna1a/Smad3/Syn3/Pnkd/Psen1/Akt1/Nrn1/Htr1a/Colq/Ptk2b/Ptger4/Gdnf/Rims2/Dpys/Asic1/Syt2/Stxbp5l/Gart/Btbd9/Prkce/Camk2a/Adrb2/Nrxn2/Dagla/Syt7/Apba1/Ass1/Chrnb2/Cnr1/Htr6/Mtor/Pkd2/Dynll1/Daglb/Dgki*	1.846	0.0478	*Nos1/Rph3a/Dgki/Hrh1/Vamp1/Brsk1/Grik5/Akt2/Apba2/Tomt/Syt9/Gcsh/Smad3/Pnkd/Nrn1/Agtpbp1/Colq/Ptger4/Gdnf/Dpys/Syt2/Gart/Aif1/Sncaip/Camk2a/Syt7/Chrnb2/Cnr1/Mtor*
GO:0010720	positive regulation of cell development	1.73515086	1E-04	*Ankrd27/Gdpd5/Dock1/Nkx6-2/Shank2/Crtc1/Pou4f2/Cx3cl1/Trim67/Pin1/Apoa1/Tle6/Zfp609/Nck1/Twf2/Actr2/Pafah1b1/Mmd/Spag9/Ngfr/Rara/Wnt3/Obsl1/Id2/Nin/Psen1/Rims1/Ror2/Arsb/Rarb/Wnt5a/Mapk8/Prmt5/Gli2/Ptk2b/Stk24/Actr3/Rims2/Triobp/Etv5/Syt2/Kalrn/Tiam1/Itsn1/Qk/Srf/Trip10/Tgif1/Sema6a/Fbxo38/Tcf4/Dcc/Vldlr/Adrb1/Camk1d/Dab2ip/Dlx2/Itga6/Nfe2l2/Bmp7/Cdh4/Slc9b2/Fubp1/Cnr1/Trim32/Dab1/Ptprf/Foxo6/Ephb2/Spen/Tnfrsf1b/Mtor/Smarcd3/Adra2c/Phox2b/Rasal1/Cux2/Ocm/Plxna4/Fig4/Grip2/Plxnd1*	1.924	0.0004	*Rasal1/Cux2/Plxna4/Grip2/Pparg/Myadm/Myod1/Cib1/Fes/Gdpd5/Rassf10/Nkx6-2/Pou4f2/Cx3cl1/Pin1/Sema7a/Zfp609/Llph/Mmd/Nme1/Spag9/Rnf157/Epha4/Rims1/Rarb/Mapk8/Stk24/Triobp/Syt2/Kalrn/Adamts1/Tiam1/Itsn1/Qk/Trip10/Sema6a/Bad/Vldlr/Adrb1/Abl1/Nfe2l2/Bmp7/Cdh4/Ss18l1/Slc9b2/Cnr1/Musk/Trim32/Dab1/Dmrta2/Foxo6/Spen/Tnfrsf1b/Mtor/Smarcd3/Adra2c/Phox2b/Hdac2*

GO:0045597	positive regulation of cell differentiation	1.72533069	1E-07	*Sox5/Foxa3/Ppp1r13l/Nfkbid/Ankrd27/Sult2b1/Gdpd5/Arntl/Dock1/Nkx6-2/Shank2/Zfp703/Crtc1/Pou4f2/Cx3cl1/Trim67/Pin1/Bcl9l/Apoa1/Tle6/Smad3/Zfp609/Nck1/Twf2/Pa2g4/Nf2/Actr2/Irf1/Pafah1b1/Lgals9/Mmd/Spag9/Ngfr/Rara/Ccr7/Wnt3/Obsl1/Id2/Nin/Psen1/Fos/Rims1/Akt1/Syk/Ror2/Arsb/Ap3b1/Pik3r1/Rarb/Wnt5a/Mapk8/Gdf10/Zfp219/Prmt5/Wdfy2/Gli2/Ptk2b/Rgcc/Stk24/Ptger4/Gdnf/Actr3/Rims2/Adgrb1/Triobp/Vdr/Etv5/Syt2/Kalrn/Tiam1/Itsn1/Runx1/Qk/Spdef/H2-Aa/Srf/Trip10/Tgif1/Socs5/Sema6a/Ppargc1b/Fbxo38/Malt1/Tcf4/Dcc/Zbtb7c/Smad2/Vldlr/Adrb1/Camk1d/Pax8/Olfm1/Dab2ip/Dlx2/Itga6/Nfe2l2/Jag1/Zhx3/Stk4/Bmp7/Cdh4/Il12a/Sfrp2/Slc9b2/Bmpr1b/Fubp1/Cnr1/Glipr2/Trim32/Dab1/Ptch2/Ptprf/Foxo6/Sfn/Runx3/Ephb2/Wnt4/Spen/Tnfrsf1b/Mtor/Uts2/Smarcd3/Adra2c/Rbpj/Phox2b/Map3k5/Rasal1/Cux2/Ocm/Plxna4/Cyp26b1/Fig4/Grip2/Foxo3/Plxnd1*	1.683	0.0004	*Rasal1/Cux2/Foxo3/Plxna4/Grip2/Pparg/Myadm/Foxa3/Ppp1r13l/Myod1/Cib1/Fes/Gdpd5/Rassf10/Nkx6-2/Nrg1/Pou4f2/Cx3cl1/Pin1/Bcl9l/Sema7a/Smad3/Zfp609/Llph/Nf2/Mmd/Nme1/Spag9/Col1a1/Ccr7/Rnf157/Epha4/Rims1/Syk/Ap3b1/Pik3r1/Rarb/Mapk8/Acin1/Stk24/Ptger4/Gdnf/Triobp/Syt2/Vdr/Trp63/Kalrn/Adamts1/Tiam1/Itsn1/Qk/Trip10/Sema6a/Ppargc1b/Malt1/Bad/Vldlr/Adrb1/Abl1/Nfe2l2/Dusp10/Jag1/Bmp7/Cdh4/Ss18l1/Slc9b2/Bmpr1b/Cnr1/Musk/Trim32/Dab1/Dmrta2/Foxo6/Runx3/Spen/Tnfrsf1b/Mtor/Uts2/Prkcz/Smarcd3/Adra2c/Rbpj/Phox2b/Hdac2*
GO:0010975	regulation of neuron projection development	1.72218512	0.0003	*Brsk1/Gsk3a/Ankrd27/Shank2/Crtc1/Pou4f2/Cacna1a/Cx3cl1/Trim67/Nedd4/Nck1/Ryk/Twf2/Amigo3/Actr2/Abi2/Pmp22/Rap1gap2/Pafah1b1/Ngfr/Wnt3/Obsl1/Nin/Psen1/Rims1/Akt1/Ror2/Arsb/Ptprg/Zswim8/Wnt5a/Ptk2b/Stk24/Actr3/Rims2/Asap1/Ephb3/Syt2/Kalrn/Tiam1/Itsn1/Qk/Srf/Sema6b/Sema6a/Fbxo38/Dcc/Vldlr/Slit1/Camk1d/Olfm1/Dab2ip/Itga6/Nfe2l2/Bmp7/Cdh4/Sfrp2/Efna1/Chrnb2/Ppp3ca/Cnr1/Dab1/Ptprf/Foxo6/Ephb2/Draxin/Mtor/Rbpj/Rasal1/Cux2/Ocm/Plxna4/Fig4/Plxnd1*	1.715	0.0153	*Trpv4/Rasal1/Cux2/Plxna4/Dguok/Brsk1/Cib1/Fes/Pou4f2/Cx3cl1/Sema7a/Llph/Nme1/Rnf157/Epha4/Rims1/Mylip/Spock1/Ptprg/Nfatc4/Rap2a/Stk24/Asap1/Syt2/Kalrn/Adamts1/Tiam1/Itsn1/Qk/Sema6a/Vldlr/Abl1/Nfe2l2/Bmp7/Cdh4/Ss18l1/Efna1/Chrnb2/Sema6c/Cnr1/Musk/Dab1/Foxo6/Draxin/Mtor/Rbpj/Hdac2*
GO:2000146	negative regulation of cell motility	1.70849032	0.0311	*Abcc8/Ccl25/Angpt2/Bst2/Cx3cl1/Pin1/Tmeff2/Abr/Igfbp5/Arhgdia/Rhob/Akt1/Ptprg/Clasp1/Rgcc/Stk24/Ptger4/Adgrb1/Card10/Srf/Coro1b/Dab2ip/Scai/Nfe2l2/Spred1/Frmd5/Jag1/Ptprt/Sfrp2/Wnt4/Miip/Rbpj/Stard13/Mitf/Foxo3*	2.219	0.0054	*Foxo3/Wasl/Mitf/Pparg/Abcc8/Ccl25/Angpt2/Nrg1/Cx3cl1/Pin1/Tmeff2/Srgap1/Igfbp5/Arhgdia/Clasp1/Ptprg/Rap2a/Stk24/Ptger4/Aif1/Scai/Nfe2l2/Dusp10/Frmd5/Jag1/Ptprt/Clic4/Miip/Rbpj*
GO:0022604	regulation of cell morphogenesis	1.70328727	0.0012	*Eps8/Brsk1/Hpn/Ankrd27/Rhog/Aldoa/Dock1/Pou4f2/Cacna1a/Bcl9l/Apoa1/Nedd4/Ryk/Twf2/Limd1/Actr2/Abi2/Pafah1b1/Fmnl1/Wnt3/Obsl1/Arhgdia/Rhob/Coch/Nin/Psen1/Rims1/Zswim8/Wnt5a/Ptk2b/Rhobtb2/Myo10/Actr3/Rims2/Triobp/Ephb3/Syt2/Kalrn/Tiam1/Ezr/Srf/Sema6b/Sema6a/Coro1b/Rhod/Slit1/Fam171a1/Olfm1/Arhgap15/Cdh4/Postn/Efna1/Chrnb2/Plekho1/Ppp3ca/Dab1/Ptprf/Ephb2/Rcc2/Draxin/Rasal1/Cux2/Ocm/Plxna4/Plxnd1*	1.684	0.0375	*Rasal1/Cux2/Wasl/Plxna4/Myadm/Brsk1/Hpn/Cib1/Fes/Pou4f2/Dapk3/Bcl9l/Sema7a/Limd1/Rnf157/Arhgdia/Epha4/Coch/Syne3/Rims1/Nfatc4/Rap2a/Myo10/Triobp/Syt2/Kalrn/Tiam1/Sema6a/Rhod/Abl1/Arhgap15/Cdh4/Ss18l1/Postn/Efna1/Chrnb2/Sema6c/Plekho1/Dab1/Rcc2/Draxin*

GO:0044057	regulation of system process	1.70200454	0.0005	*Abcc9/Calm3/Gsk3a/Sptbn4/Ryr1/Nr1h2/Abcc8/Mef2a/Ctnna3/Atp2a1/Mtg1/Shank2/Tmem38a/Cx3cl1/Pin1/Apoa1/Smad3/Tmem108/Scn11a/Gls/Atp2b1/Smtn/Nmur2/Srebf1/Asic2/Igfbp5/Cacna1g/Tac4/Ngfr/Myl4/Sstr2/Akt1/Jarid2/Cartpt/Pik3r1/Ptk2b/Ptger4/Rims2/Gm28040/Fgf12/Tnnt2/Tnfrsf21/Srf/Sh3gl1/Trip10/Adrb2/Dusp5/Adrb1/Nos1ap/Cry2/Mc3r/Mtg2/Ank2/Ppp3ca/Edn2/Ece1/Tnfrsf1b/Mtor/Errfi1/Uts2/Kcnh2/Adra2c/Phox2b/Cux2/Myl2/Hcar2/Gtf2ird2/Tbxas1/Fig4/Grip2/Hrh1/Foxo3*	1.890	0.0017	*Nos1/Cux2/Myl2/Foxo3/Grip2/Hrh1/Abcc9/Ctnna3/Abcc8/Atp2a1/Tmem38a/Fto/Cx3cl1/Pin1/Hcn4/Smad3/Tmem108/Scn11a/Cck/Smtn/Slc22a5/Srebf1/Asic2/Igfbp5/Cacna1g/Sstr2/Inha/Gaa/Nptx1/Begain/Hrh2/Homer1/Pik3r1/Inhbb/Ptger4/Shisa9/Aif1/Sh3gl1/Trip10/Nos1ap/Dusp5/Adrb1/Snta1/Edn2/Trim63/Tnfrsf1b/Mtor/Uts2/Prkcz/Adra2c/Phox2b*
GO:0023057	negative regulation of signaling	1.69919595	6E-09	*Ybx3/Calm3/Ppm1n/Gsk3a/Nfkbia/Pin1a1/Abcc8/Crtc3/Ndufc2/Tsku/Gdpd5/Pde2a/Arntl/Nucb2/Mvp/Ptpre/Shank2/Tmem161a/Pde4c/Pfkl/Nkd1/Cx3cl1/Trim67/Egln1/Sesn3/Pin1/Cdkn2d/Zbtb7a/Nlrx1/Bcl9l/Apoa1/Cactin/Tle6/Slc24a1/Nedd4/Nck1/Nprl2/Limd1/Otud5/Emd/Irak3/Nf2/Grb10/Slit3/Irf1/Gria1/Srebf1/Epn2/Pafah1b1/Mnt/Nxn/Lgals9/Aatf/Igfbp5/Pnkd/Tob1/Wfikkn2/Ngfr/Socs7/Nploc4/Esr2/Psen1/Lgmn/Gsc/Kif26a/Akt1/Trim27/Serpinb1b/Tmem170b/Ror2/Pde8b/Cartpt/Vdac2/Wnt5a/Lgals3/Ndrg2/Ptk2b/Rgcc/Ubac2/Ptger4/Gdnf/Rrm2b/Pla2g6/Ttll12/Gramd4/Hdac7/Asic1/Tns2/Heg1/Kalrn/Stxbp5l/Ezr/Pde10a/Ivns1abp/Lemd2/Tnfrsf21/Rgs8/Srf/Plcl2/Tgif1/Lbh/Ltbp1/Socs5/Arhgap12/Sema6a/Adrb2/Tbc1d10c/Fam89b/Lzts2/Sufu/Dusp5/Pdcd4/Dab2ip/Scai/Dlx2/Itga6/Nfe2l2/Dgkz/Mapk8ip1/Cry2/Spred1/Jag1/Ptprt/Stk4/Sulf2/Kcnb1/Bmp7/Ift80/Sfrp2/Pear1/Efna1/Adar/Ppp3ca/Cnr1/Trim32/Dab1/Prkaa2/Trabd2b/Ptprf/Edn2/Kcnq4/Ephb2/Wnt4/Camk2n1/Htr6/Epha2/Draxin/Mtor/Errfi1/Uts2/Nphp4/Ski/Cptp/Htra3/Wfs1/Arhgap24/Pkd2/Fgfrl1/Rasal1/Ncor2/Bmt2/Cyp26b1/Anxa4/Sesn1/Foxo3*	1.598	0.0005	*Nos1/Rasal1/Ncor2/Sesn1/Foxo3/Grid2ip/Pparg/Ift122/Myadm/Ptgir/Bax/Abcc8/Cib1/Ndufc2/Gdpd5/Plekha1/Rnf126/Dusp26/Pde4c/Nkd1/Cx3cl1/Cactin/Egln1/Sesn3/Pin1/Bcl9l/Limd1/Irak3/Prdm14/Nf2/Gria1/Srebf1/Epn2/Nxn/Igfbp5/Wfikkn2/Pnkd/Socs7/Rgs9/Inha/Esr2/Gsc/Dlk1/Trim27/Serpinb1b/Inhbb/Lgals3/Nfatc4/Ubac2/Ptger4/Gdnf/Rrm2b/Pla2g6/Pim3/Trp63/Heg1/Kalrn/Pde10a/Lemd2/Plcl2/Lbh/Ltbp1/Sema6a/Lzts2/Sufu/Dusp5/Pdcd4/Tcf7l2/Lefty1/Abl1/Scai/Nfe2l2/Dgkz/Dusp10/Jag1/Sstr4/Ptprt/Mmp9/Bmp7/Ift80/Efna1/Adar/Mcl1/Cnr1/Trim32/Tle1/Dab1/Prkaa2/Trabd2b/Edn2/Cnksr3/Epha2/Draxin/Mtor/Uts2/Nphp4/Prkcz/Htra3/Arhgap24*
GO:0060284	regulation of cell development	1.69828758	4E-07	*Sox5/Brsk1/Gsk3a/Ankrd27/Abcc8/Gdpd5/Arntl/Dock1/Nkx6-2/Shank2/Pcm1/Crtc1/Pou4f2/Cacna1a/Cx3cl1/Trim67/Pin1/S1pr5/Apoa1/Tle6/Zfp609/Nedd4/Nck1/Ryk/Twf2/Amigo3/Nf2/Actr2/Abi2/Pmp22/Rap1gap2/Pafah1b1/Mmd/Spag9/Ngfr/Rara/Wnt3/Obsl1/Id2/Nin/Psen1/Bcl11b/Rims1/Akt1/Ror2/Arsb/Ptprg/Rarb/Zswim8/Wnt5a/Mapk8/Prmt5/Gli2/Ptk2b/Stk24/Actr3/Rims2/Asap1/Triobp/Slc45a3/Ephb3/Etv5/Syt2/Kalrn/Tiam1/Itsn1/Qk/Notch3/Tnfrsf21/Srf/Sema6b/Trip10/Tgif1/Sema6a/Camk2a/Fbxo38/Tcf4/Dcc/Vldlr/Slit1/Adrb1/Vax1/Camk1d/Olfm1/Dab2ip/Dlx2/Itga6/Nfe2l2/Eif2ak4/Jag1/Bmp7/Cdh4/Postn/Igsf10/Sfrp2/Efna1/Chrnb2/Slc9b2/Ppp3ca/Eif4e/Fubp1/Cnr1/Trim32/Dab1/Ptprf/Foxo6/Ephb2/Rap1gap/Rcc2/Spen/Tnfrsf1b/Draxin/Disp3/Mtor/Hes5/Ski/Smarcd3/Adra2c/Rbpj/Phox2b/Rasal1/Cux2/Rflna/Ywhag/Ocm/Plxna4/Fig4/Grip2/Atg7/Foxo3/Plxnd1*	1.850	1E-05	*Trpv4/Nos1/Rasal1/Cux2/Foxo3/Plxna4/Dguok/Grip2/Atg7/Pparg/Myadm/Brsk1/Abcc8/Myod1/Cib1/Fes/Gdpd5/Rassf10/Nkx6-2/Nrg1/Pcm1/Pou4f2/Cx3cl1/Pin1/S1pr5/Sema7a/Zfp609/Llph/Nf2/Mmd/Nme1/Spag9/Rnf157/Epha4/Rims1/Mylip/Spock1/Ptprg/Rarb/Mapk8/Nfatc4/Rap2a/Stk24/Gpr183/Asap1/Triobp/Syt2/Kalrn/Adamts1/Tiam1/Itsn1/Qk/Notch3/Trip10/Sema6a/Camk2a/Bad/Vldlr/Tcf7l2/Adrb1/Vax1/Abl1/Nfe2l2/Dusp10/Jag1/Bmp7/Cdh4/Ss18l1/Postn/Efna1/Chrnb2/Sema6c/Slc9b2/Eif4e/Cnr1/Musk/Trim32/Dab1/Dmrta2/Foxo6/Rcc2/Spen/Tnfrsf1b/Draxin/Mtor/Casz1/Smarcd3/Adra2c/Rbpj/Phox2b/Hdac2*

GO:0030336	negative regulation of cell migration	1.69661162	0.0426	*Abcc8/Ccl25/Angpt2/Bst2/Cx3cl1/Tmeff2/Abr/Igfbp5/Arhgdia/Rhob/Akt1/Ptprg/Clasp1/Rgcc/Stk24/Ptger4/Adgrb1/Card10/Srf/Coro1b/Dab2ip/Scai/Nfe2l2/Spred1/Jag1/Ptprt/Sfrp2/Wnt4/Miip/Rbpj/Stard13/Mitf/Foxo3*	2.176	0.0102	*Foxo3/Wasl/Mitf/Pparg/Abcc8/Ccl25/Angpt2/Nrg1/Cx3cl1/Tmeff2/Srgap1/Igfbp5/Arhgdia/Clasp1/Ptprg/Rap2a/Stk24/Ptger4/Aif1/Scai/Nfe2l2/Dusp10/Jag1/Ptprt/Clic4/Miip/Rbpj*
GO:0120035	regulation of plasma membrane bounded cell projection organization	1.69469222	5E-05	*Eps8/Brsk1/Gsk3a/Ankrd27/Rhog/Shank2/Crtc1/Pou4f2/Cacna1a/Cx3cl1/Def8/Trim67/Nedd4/Nck1/Ryk/Twf2/Amigo3/Tbc1d30/Actr2/Abi2/Pmp22/Rap1gap2/Pafah1b1/Bcas3/Ngfr/Mien1/Ccr7/Wnt3/Obsl1/Nin/Psen1/Rims1/Akt1/Ror2/Cdhr2/Arsb/Ptprg/Zswim8/Wnt5a/Ptk2b/Stk24/Myo10/Actr3/Rims2/Asap1/Kctd17/Ephb3/Syt2/Kalrn/Tiam1/Itsn1/Ezr/Qk/Srf/Sema6b/Sema6a/Cep120/Fbxo38/Dcc/Coro1b/Vldlr/Slit1/Camk1d/Olfm1/Dab2ip/Itga6/Nfe2l2/Bmp7/Cdh4/Sfrp2/Efna1/Chrnb2/Ppp3ca/Cnr1/Dab1/Ptprf/Foxo6/Ephb2/Rap1gap/Rcc2/Epha2/Draxin/Mtor/Tapt1/Rbpj/Arhgap24/Dynll1/Rasal1/Cux2/Fscn1/Ocm/Plxna4/Fig4/Plxnd1*	1.668	0.0075	*Trpv4/Rasal1/Cux2/Wasl/Plxna4/Dguok/Noto/Brsk1/Cib1/Fes/Pou4f2/Cx3cl1/Sema7a/Llph/Nme1/Ccr7/Rnf157/Epha4/Rims1/Mylip/Cdhr2/Spock1/Homer1/Ptprg/Nfatc4/Rap2a/Stk24/Myo10/Asap1/Syt2/Kalrn/Adamts1/Tiam1/Itsn1/Qk/Sema6a/Cep120/Vldlr/Abl1/Nfe2l2/Bmp7/Cdh4/Ss18l1/Efna1/Chrnb2/Sema6c/Odf2l/Cnr1/Musk/Dab1/Foxo6/Rcc2/Crocc/Epha2/Draxin/Mtor/Rbpj/Hdac2/Arhga*
GO:0030030	cell projection organization	1.69421892	1E-05	*Dnaaf3/Brsk1/Atp1a3/Sptbn4/Ankrd27/Mef2a/Tsku/Gdpd5/Rhog/Ank3/Shank2/Myo16/Pcm1/Palld/Pou4f2/Cacna1a/Vstm5/Bbs9/Zpr1/Nedd4/Nck1/Ryk/Tmem108/Twf2/Tmeff2/Bbs10/Pla2g3/Actr2/Abi2/Pmp22/Pafah1b1/Rab10/Nin/Psen1/Lgmn/Bcl11b/Akt1/Actn2/Nrn1/Unc5a/Kif27/Wnt5a/Ercc6/Mapk8/Gli2/Ptk2b/Gdnf/Actr3/Lrp12/Asap1/Lrrc6/Triobp/Parvg/Ttll8/Ephb3/Kalrn/Ezr/Lhfpl5/Ddr1/Tnfrsf21/Srf/Rab23/Camk2a/Ablim3/Dcc/Coro1b/Rhod/Vldlr/Slit1/Ablim1/Ntng2/Dab2ip/Bbs5/Itga6/Ttc30a1/Map1a/Stk4/Bmp7/Ift80/Fhdc1/Chrnb2/Pip5k1a/Plekho1/Cnr1/Trim32/Dab1/Spata6/Ptprf/Runx3/Ephb2/Mtor/Hes5/Tapt1/Wdr19/Phox2b/Sept11/Pkd2/Dynll1/Bicdl1/Ift81/Actb/Fscn1/Plxna4/Wasf1/Atg7*	1.644	0.0057	*Rph3a/Wasf1/Actb/Wasl/Plxna4/Noto/Atg7/Ift122/Dnaaf3/Brsk1/Gdpd5/Plekha1/Myo16/Pcm1/Pou4f2/Tmeff2/Zpr1/Sema7a/Tmem108/Poc1a/Cck/Cep290/Pla2g3/Nptx1/Epha4/Nrn1/Mapk8/Rap2a/Gdnf/Lrp12/Asap1/Lrrc6/Triobp/Parvg/Ttll8/Kalrn/Arhgef4/Aif1/Camk2a/Ablim3/Rhod/Vldlr/Ablim1/Cdnf/Ntng2/Abl1/Bbs5/Ttc30a1/Map1a/Spg11/Bmp7/Ss18l1/Ift80/Fhdc1/Chrnb2/Plekho1/Odf2l/Cnr1/Trim32/Dab1/Spata6/Runx3/Crocc/Mtor/Phox2b*
GO:0010648	negative regulation of cell communication	1.69405867	7E-09	*Ybx3/Calm3/Ppm1n/Gsk3a/Nfkbid/Pih1d1/Abcc8/Crtc3/Ndufc2/Tsku/Gdpd5/Pde2a/Arntl/Nucb2/Ptpre/Shank2/Tmem161a/Pde4c/Pfkl/Nkd1/Cx3cl1/Trim67/Egln1/Sesn3/Pin1/Cdkn2d/Zbtb7a/Nlrx1/Bcl9l/Apoa1/Cactin/Tle6/Slc24a1/Nedd4/Nck1/Nprl2/Limd1/Otud5/Emd/Irak3/Nf2/Grb10/Slit3/Irf1/Gria1/Srebf1/Epn2/Pafah1b1/Mnt/Nxn/Lgals9/Aatf/Igfbp5/Pnkd/Tob1/Wfikkn2/Ngfr/Socs7/Nploc4/Esr2/Psen1/Lgmn/Gsc/Kif26a/Akt1/Trim27/Serpinb1b/Tmem170b/Ror2/Pde8b/Cartpt/Vdac2/Wnt5a/Lgals3/Ndrg2/Ptk2b/Rgcc/Ubac2/Ptger4/Gdnf/Rrm2b/Pla2g6/Ttll12/Gramd4/Hdac7/Asic1/Tns2/Heg1/Kalrn/Stxbp5l/Ezr/Pde10a/Ivns1abp/Lemd2/Tnfrsf21/Rgs8/Srf/Plcl2/Tgif1/Lbh/Ltbp1/Socs5/Arhgap12/Sema6a/Adrb2/Tbc1d10c/Fam89b/Lzts2/Sufu/Dusp5/Pdcd4/Dab2ip/Scai/Dlx2/Itga6/Nfe2l2/Dgkz/Mapk8ip1/Cry2/Spred1/Jag1/Ptprt/Stk4/Sulf2/Kcnb1/Bmp7/Ift80/Sfrp2/Pear1/Efna1/Adar/Ppp3ca/Cnr1/Trim32/Dab1/Prkaa2/Trabd2b/Ptprf/Edn2/Kcnq4/Ephb2/Wnt4/Camk2n1/Htr6/Epha2/Draxin/Mtor/Errfi1/Uts2/Nphp4/Ski/Cptp/Htra3/Wfs1/Arhgap24/Pkd2/Fgfrl1/Rasal1/Ncor2/Bmt2/Cyp26b1/Anxa4/Sesn1/Foxo3*	1.603	0.0004	*Nos1/Rasal1/Ncor2/Sesn1/Foxo3/Grid2ip/Pparg/Ift122/Myadm/Ptgir/Bax/Abcc8/Cib1/Ndufc2/Gdpd5/Plekha1/Rnf126/Dusp26/Pde4c/Nkd1/Cx3cl1/Cactin/Egln1/Sesn3/Pin1/Bcl9l/Limd1/Irak3/Prdm14/Nf2/Gria1/Srebf1/Epn2/Nxn/Igfbp5/Wfikkn2/Pnkd/Socs7/Rgs9/Inha/Esr2/Gsc/Dlk1/Trim27/Serpinb1b/Inhbb/Lgals3/Nfatc4/Ubac2/Ptger4/Gdnf/Rrm2b/Pla2g6/Pim3/Trp63/Heg1/Kalrn/Pde10a/Lemd2/Plcl2/Lbh/Ltbp1/Sema6a/Lzts2/Sufu/Dusp5/Pdcd4/Tcf7l2/Lefty1/Abl1/Scai/Nfe2l2/Dgkz/Dusp10/Jag1/Sstr4/Ptprt/Mmp9/Bmp7/Ift80/Efna1/Adar/Mcl1/Cnr1/Trim32/Tle1/Dab1/Prkaa2/Trabd2b/Edn2/Cnksr3/Epha2/Draxin/Mtor/Uts2/Nphp4/Prkcz/Htra3/Arhgap24*

GO:0034765	regulation of ion transmembrane transport	1.69371363	0.0026	*Calm3/Akt2/Kcna7/Abcc8/Kcnc1/Cacng3/Atp2a1/Ank3/Mrln/Shank2/Irs2/Tmem38a/Cacna1a/Cx3cl1/Nedd4/Gnb5/Scn11a/Cacna1f/Ramp3/Kcnj12/Asic2/Cacna1g/Psen1/Kcnk13/Akt1/Actn2/Slc34a1/Rem2/Ptk2b/Tmem37/Fgf14/Pla2g6/Rapgef3/Kcnh3/Clcn2/Fgf12/Cox17/Kcnj6/Kcng3/Prkce/Adrb2/Kcng2/Kcnip2/Adrb1/Nos1ap/Clic3/Psen2/Kcnb1/Ahcyl1/Ank2/Ptpn3/Kcnh1/Kcnq4/Ephb2/Mtor/Kcnh2/Pkd2/Tpcn1/Fxyd4/Cacna2d4*	1.556	0.4082	*Nos1/Akt2/Mrln/Gnb5/Ywhaq/Rem2/Mmp9*
GO:0031344	regulation of cell projection organization	1.67459706	8E-05	*Eps8/Brsk1/Gsk3a/Ankrd27/Rhog/Shank2/Crtc1/Pou4f2/Cacna1a/Cx3cl1/Def8/Trim67/Nedd4/Nck1/Ryk/Twf2/Amigo3/Tbc1d30/Actr2/Abi2/Pmp22/Rap1gap2/Pafah1b1/Bcas3/Ngfr/Mien1/Ccr7/Wnt3/Obsl1/Nin/Psen1/Rims1/Akt1/Ror2/Cdhr2/Arsb/Ptprg/Zswim8/Wnt5a/Ptk2b/Stk24/Myo10/Actr3/Rims2/Asap1/Kctd17/Ephb3/Syt2/Kalrn/Tiam1/Itsn1/Ezr/Qk/Srf/Sema6b/Sema6a/Cep120/Fbxo38/Dcc/Coro1b/Vldlr/Slit1/Camk1d/Olfm1/Dab2ip/Itga6/Nfe2l2/Bmp7/Cdh4/Sfrp2/Efna1/Chrnb2/Ppp3ca/Cnr1/Dab1/Ptprf/Foxo6/Ephb2/Rap1gap/Rcc2/Epha2/Draxin/Mtor/Tapt1/Rbpj/Arhgap24/Dynll1/Rasal1/Cux2/Fscn1/Ocm/Plxna4/Fig4/Plxnd1*	1.648	0.0095	*Trpv4/Rasal1/Cux2/Wasl/Plxna4/Dguok/Noto/Brsk1/Cib1/Fes/Pou4f2/Cx3cl1/Sema7a/Llph/Nme1/Ccr7/Rnf157/Epha4/Rims1/Mylip/Cdhr2/Spock1/Homer1/Ptprg/Nfatc4/Rap2a/Stk24/Myo10/Asap1/Syt2/Kalrn/Adamts1/Tiam1/Itsn1/Qk/Sema6a/Cep120/Vldlr/Abl1/Nfe2l2/Bmp7/Cdh4/Ss18l1/Efna1/Chrnb2/Sema6c/Odf2l/Cnr1/Musk/Dab1/Foxo6/Rcc2/Crocc/Epha2/Draxin/Mtor/Rbpj/Hdac2/Arhga*
GO:0043065	**positive regulation of apoptotic process**	1.62778658	0.0013	*Ube2m/Tomm40/Grik5/Gsk3a/Nfkbid/Pdcd5/Map2k7/Ido1/Pou4f2/Egln1/Pin1/Ppp2r1b/Smad3/Nck1/Ip6k2/Ngfr/Rhob/Esr2/Psen1/Akt1/Eef1e1/Syk/Rarb/Wnt5a/Mapk8/Sox7/Rgcc/Dap/* ***Pla2g6*** */Atf4/Gramd4/Vdr/Nr4a1/Tfap4/Eif2b5/Traf7/Spdef/Ralbp1/Camk2a/Adrb2/Malt1/Calhm2/* ***Pdcd4*** */Adrb1/Camk1d/Olfm1/Dab2ip/Itga6/Prdm11/Rbck1/Ctnnbl1/Stk4/Bmp7/Il12a/Sfrp2/Bmpr1b/Bcl10/Casp8ap2/Cnr1/Acer2/Scp2/Ptprf/Runx3/Rpl11/Casp9/Tnfrsf1b/Il20ra/Map3k5/Hcar2/Tpd52l1/Ing3/Rybp/Atg7/Foxo3/Gapdh*	1.872	0.0011	*Foxo3/Rybp/Atg7/Pparg/Gapdh/Grik5/Pdcd5/Bax/Pou4f2/Dapk3/Egln1/Pin1/Ppp2r1b/Smad3/Apaf1/Ip6k2/Cck/Spop/Esr2/Eef1e1/Syk/Rarb/Mapk8/Inhbb/Acin1/Nfatc4/Dap/* ***Pla2g6*** */Vdr/Trp63/Fndc1/Camk2a/Malt1/Pmaip1/Cd248/Map3k11/Bad/Calhm2/* ***Pdcd4*** */Tcf7l2/Adrb1/Abl1/Ctnnbl1/Serinc3/Mmp9/Bmp7/Mcl1/Bmpr1b/Casp8ap2/Cnr1/Musk/Acer2/Runx3/Rpl11/Tnfrsf1b*
GO:0010942	**positive regulation of cell death**	1.61627026	0.0008	*Ube2m/Tomm40/Grik5/Gsk3a/Nfkbid/Pdcd5/Map2k7/Ido1/Pou4f2/Egln1/Pin1/Ppp2r1b/Smad3/Nck1/Ip6k2/Elk1/Ramp3/Irf1/Ngfr/Rhob/Esr2/Psen1/Fos/Akt1/Eef1e1/Syk/Rarb/Wnt5a/Mapk8/Sox7/Rgcc/Dap/* ***Pla2g6*** */Atf4/Gramd4/Vdr/Nr4a1/Tfap4/Eif2b5/Traf7/Spdef/Daxx/Ralbp1/Camk2a/Adrb2/Malt1/Lzts2/Calhm2/* ***Pdcd4*** */Adrb1/Camk1d/Olfm1/Dab2ip/Itga6/Prdm11/Rbck1/Ctnnbl1/Stk4/Bmp7/Il12a/Sfrp2/Bmpr1b/Bcl10/Casp8ap2/Cnr1/Acer2/Scp2/Ptprf/Runx3/Rpl11/Casp9/Tnfrsf1b/Mtor/Il20ra/Map3k5/Hcar2/Tpd52l1/Ing3/Rybp/Atg7/Foxo3/Gapdh*	1.823	0.0012	*Nos1/Foxo3/Rybp/Atg7/Pparg/Gapdh/Grik5/Pdcd5/Bax/Pou4f2/Dapk3/Egln1/Pin1/Ppp2r1b/Smad3/Apaf1/Ip6k2/Cck/Spop/Esr2/Eef1e1/Syk/Rarb/Mapk8/Inhbb/Acin1/Nfatc4/Dap/* ***Pla2g6*** */Vdr/Trp63/Fndc1/Camk2a/Malt1/Pmaip1/Cd248/Map3k11/Bad/Lzts2/Calhm2/* ***Pdcd4*** */Tcf7l2/Adrb1/Abl1/Ctnnbl1/Serinc3/Mmp9/Bmp7/Kcnk2/Mcl1/Bmpr1b/Casp8ap2/Cnr1/Musk/Acer2/Runx3/Rpl11/Tnfrsf1b/Mtor*

GO:0030001	metal ion transport	1.61519392	0.0027	*Abcc9/Atp1a3/Ryr1/Atp4a/Hpn/Kcna7/Abcc8/Kcnc1/Trpm1/Cacng3/Atp2a1/Tmem38a/Cacna1a/Slc12a4/Kcnk1/Slc39a10/Slc24a1/Plcd1/Scn11a/Cacna1f/Atp2b1/Ramp3/Nmur2/Kcnj12/Slc13a5/Asic2/Cacna1g/Slc38a10/Psen1/Kcnk13/Sfxn1/Slc34a1/Trpc7/Klhl3/Slc28a3/Ap3b1/Tmem37/Tmem163/Vdr/Kcnh3/Asic1/Cox17/Cpox/Slc5a3/Kcnj6/Rab11b/Plcl2/Kcng3/Kcnk12/Camk2a/Kcng2/Kcnip2/Slc25a25/Slc39a13/Slc12a6/Kcnb1/Kcnmb3/Chrnb2/Slc9b2/Ppp3ca/Kcnh1/Kcnq4/Plch2/Kcnh2/Pkd2/Tpcn1/Mfsd4b1/Hrh1/Fxyd4/Cacna2d4*	1.628	0.0411	*Mfsd4b1/Trpv4/Hrh1/Cacna2d4/Abcc9/Atp4a/Hpn/Abcc8/Kcnc1/Atp2a1/Orai3/Cracr2b/Tusc3/Tmem38a/Slc12a4/Jph3/Hcn4/Slc17a8/Scn11a/Slc22a5/Kcnj12/Slc13a5/Asic2/Cltc/Cacna1g/Ramp1/Sfxn1/Klhl3/Ap3b1/Tmem163/Vdr/Kcnh3/Scn8a/Kcnj6/Plcl2/Kcng3/Kcnn2/Camk2a/Slc25a25/Slc12a6/Plcb4/Kcnk2/Kcnmb3/Chrnb2/Slc9b2*
GO:0043068	positive regulation of programmed cell death	1.61482649	0.0017	*Ube2m/Tomm40/Grik5/Gsk3a/Nfkbid/Pdcd5/Map2k7/Ido1/Pou4f2/Egln1/Pin1/Ppp2r1b/Smad3/Nck1/Ip6k2/Ngfr/Rhob/Esr2/Psen1/Akt1/Eef1e1/Syk/Rarb/Wnt5a/Mapk8/Sox7/Rgcc/Dap/Pla2g6/Atf4/Gramd4/Vdr/Nr4a1/Tfap4/Eif2b5/Traf7/Spdef/Ralbp1/Camk2a/Adrb2/Malt1/Calhm2/Pdcd4/Adrb1/Camk1d/Olfm1/Dab2ip/Itga6/Prdm11/Rbck1/Ctnnbl1/Stk4/Bmp7/Il12a/Sfrp2/Bmpr1b/Bcl10/Casp8ap2/Cnr1/Acer2/Scp2/Ptprf/Runx3/Rpl11/Casp9/Tnfrsf1b/Il20ra/Map3k5/Hcar2/Tpd52l1/Ing3/Rybp/Atg7/Foxo3/Gapdh*	1.857	0.0014	*Foxo3/Rybp/Atg7/Pparg/Gapdh/Grik5/Pdcd5/Bax/Pou4f2/Dapk3/Egln1/Pin1/Ppp2r1b/Smad3/Apaf1/Ip6k2/Cck/Spop/Esr2/Eef1e1/Syk/Rarb/Mapk8/Inhbb/Acin1/Nfatc4/Dap/Pla2g6/Vdr/Trp63/Fndc1/Camk2a/Malt1/Pmaip1/Cd248/Map3k11/Bad/Calhm2/Pdcd4/Tcf7l2/Adrb1/Abl1/Ctnnbl1/Serinc3/Mmp9/Bmp7/Mcl1/Bmpr1b/Casp8ap2/Cnr1/Musk/Acer2/Runx3/Rpl11/Tnfrsf1b*
GO:0043269	regulation of ion transport	1.61422169	0.0006	*Calm3/Sptbn4/Akt2/Kcna7/Abcc8/Kcnc1/Il16/Syt9/Cacng3/Atp2a1/Ank3/Mrln/Shank2/Irs2/Tmem38a/Cacna1a/Cx3cl1/Apoa1/Nedd4/Gnb5/Scn11a/Cacna1f/Atp2b1/Ramp3/Kcnj12/Asic2/Cacna1g/Psen1/Kcnk13/Akt1/Actn2/Trim27/Syk/Slc34a1/Htr1a/Lgals3/Rem2/Ptk2b/Tmem37/Cldn10/Fgf14/Gdnf/Pla2g6/Pdgfb/Atf4/Mchr1/Rapgef3/Vdr/Kcnh3/Clcn2/Fgf12/Cox17/Kcnj6/Sik1/Rab11b/Kcng3/Prkce/Camk2a/Adrb2/Kcng2/Chrm1/Syt7/Kcnip2/Adrb1/Nos1ap/Clic3/Psen2/Kcnb1/Chrnb2/Ahcyl1/Ank2/Cnr1/Ptpn3/Kcnh1/Kcnq4/Ephb2/Htr6/Mtor/Kcnh2/Wfs1/Pkd2/Tpcn1/Adcyap1r1/Fxyd4/Cacna2d4*	1.787	0.0017	*Nos1/Rph3a/Adcyap1r1/Cacna2d4/Akt2/Mrln/Bax/Abcc8/Kcnc1/Il16/Syt9/Atp2a1/Cracr2b/Tmem38a/Cx3cl1/Jph3/Hcn4/Gnb5/Scn11a/Cck/Kcnj12/Asic2/Cacna1g/Rgs9/Ywhaq/Trim27/Syk/Hrh2/Homer1/Lgals3/Rem2/Cldn10/Fgf14/Gdnf/Pla2g6/Vdr/Kcnh3/Scn8a/Shisa9/Clcn2/Kcnj6/Kcng3/Lhcgr/Kcnn2/Camk2a/Nos1ap/Syt7/Adrb1/Clic3/Abl1/Plcb4/Sstr4/Snta1/Mmp9/Chrnb2/Ahcyl1/Cnr1/Cnksr3/Clic4/Mtor*
GO:0010721	negative regulation of cell development	1.59294154	0.0369	*Gsk3a/Abcc8/Nkx6-2/Pcm1/Pou4f2/Ryk/Amigo3/Pmp22/Rap1gap2/Pafah1b1/Ngfr/Wnt3/Id2/Psen1/Ptprg/Wnt5a/Actr3/Asap1/Notch3/Sema6b/Sema6a/Slit1/Vax1/Dlx2/Eif2ak4/Jag1/Bmp7/Postn/Efna1/Ppp3ca/Eif4e/Dab1/Ephb2/Rap1gap/Rcc2/Draxin/Disp3/Hes5/Ski/Rbpj/Phox2b/Rflna/Foxo3*	1.917	0.0177	*Trpv4/Foxo3/Dguok/Abcc8/Cib1/Nkx6-2/Nrg1/Pcm1/Pou4f2/Sema7a/Epha4/Mylip/Spock1/Ptprg/Nfatc4/Asap1/Notch3/Sema6a/Vax1/Dusp10/Jag1/Bmp7/Postn/Efna1/Sema6c/Eif4e/Dab1/Rcc2/Draxin/Casz1/Rbpj/Phox2b/Hdac2*

GO:0007010	cytoskeleton organization	1.58063603	0.0003	*Eps8/Brsk1/Calm3/Sptbn4/Mef2a/Uvrag/Arhgef17/Rhog/Mypn/Ank3/Cfap46/Specc1l/Pcm1/Palld/Capn9/Zpr1/Vill/Limd1/Limk2/Pla2g3/Smtn/Nf2/Actr2/Abi2/Aurkb/Pafah1b1/Abr/Kif2b/Kpnb1/Ccr7/Fmnl1/Prkar1a/Obsl1/Rhob/Nin/Actn1/Eml1/Cdc42bpb/Akt1/Actn2/Tubb2a/Pfn3/Mast4/Actbl2/Ccser2/Clasp1/Ptk2b/Rhobtb2/Actr3/Nckap5/Adgrb1/Eppk1/Plec/Parvg/Tuba1b/Gm49450/Tuba1c/Racgap1/Itgb5/Son/Nav1/Tnnt2/Ezr/Rsph1/Kifc1/Srf/Trip10/Ralbp1/Cript/Arhgap26/Cep120/Synpo/Ablim3/Coro1b/Rhod/Lzts2/Ablim1/Nuf2/Fam171a1/Nos1ap/Sptan1/Ttll11/Map1a/Frmd5/Fhdc1/Pip5k1a/Psrc1/Ank2/Rap1gds1/Epb41l4b/Trim32/Myom3/Myl2/Sun1/Actb/Fscn1/Gm42791/Mad2l1/Wasf1/Iqsec1/Gapdh*	1.644	0.0048	*Trpv4/Myl2/Wasf1/Actb/Grid2ip/Wasl/Gapdh/Mypn/Brsk1/Ccdc8/Cib1/Fes/Mical2/Pcm1/Capn9/Cnn1/Zpr1/Cryab/Tln2/Poc1a/Limd1/Cep290/Pla2g3/Smtn/Nf2/Chd3/Cltc/Kpnb1/Ccr7/Krt14/Prkar1a/Syne3/Cdc42bpb/Tubb2a/Clasp1/Rap2a/Eppk1/Plec/Parvg/Gm49450/Tuba1c/Racgap1/Nav1/Itgb5/Spice1/Son/Rsph1/Kifc1/Trip10/Cript/Svil/Cep120/Ablim3/Nos1ap/Rhod/Lzts2/Ablim1/Sptan1/Abl1/Ttll11/Map1a/Frmd5/Fhdc1/Ubxn2b/Trim32/Myom3/Prkcz*
GO:0034762	regulation of transmembrane transport	1.57670974	0.0073	*Calm3/Gsk3a/Akt2/Kcna7/Abcc8/Kcnc1/Mef2a/Cacng3/Atp2a1/Ank3/Mrln/Shank2/Irs2/Tmem38a/Pou4f2/Cacna1a/Cx3cl1/Nedd4/Gnb5/Scn11a/Cacna1f/Ramp3/Grb10/Kcnj12/Asic2/Cacna1g/Psen1/Kcnk13/Akt1/Actn2/Slc34a1/Rem2/Ptk2b/Tmem37/Fgf14/Pla2g6/Rapgef3/Kcnh3/Clcn2/Fgf12/Cox17/Kcnj6/Kcng3/Prkce/Adrb2/Kcng2/Kcnip2/Adrb1/Nos1ap/Clic3/Nfe2l2/Psen2/Kcnb1/Ahcyl1/Ank2/Ptpn3/Kcnh1/Kcnq4/Ephb2/Mtor/Kcnh2/Pkd2/Tpcn1/Actb/Fxyd4/Cacna2d4*	1.686	0.0259	*Nos1/Rph3a/Actb/Cacna2d4/Akt2/Mrln/Bax/Abcc8/Kcnc1/Atp2a1/Tmem38a/Pou4f2/Cx3cl1/Jph3/Hcn4/Gnb5/Scn11a/Kcnj12/Asic2/Cacna1g/Rgs9/Ywhaq/Homer1/Rem2/Fgf14/Pla2g6/Kcnh3/Scn8a/Shisa9/Clcn2/Kcnj6/Kcng3/Lhcgr/Kcnn2/Nos1ap/Adrb1/Clic3/Abl1/Nfe2l2/Snta1/Mmp9/Ahcyl1/Cnksr3/Clic4/Mtor*
GO:0009968	negative regulation of signal transduction	1.57392895	2E-05	*Ybx3/Calm3/Ppm1n/Gsk3a/Nfkbid/Pih1d1/Crtc3/Ndufc2/Tsku/Gdpd5/Pde2a/Arntl/Nucb2/Ptpre/Tmem161a/Nkd1/Cx3cl1/Trim67/Egln1/Sesn3/Pin1/Cdkn2d/Zbtb7a/Nlrx1/Bcl9l/Apoa1/Cactin/Tle6/Nedd4/Nck1/Nprl2/Limd1/Emd/Irak3/Nf2/Grb10/Slit3/Irf1/Epn2/Pafah1b1/Mnt/Nxn/Aatf/Igfbp5/Tob1/Wfikkn2/Ngfr/Socs7/Nploc4/Esr2/Psen1/Lgmn/Gsc/Kif26a/Akt1/Tmem170b/Ror2/Vdac2/Wnt5a/Lgals3/Ndrg2/Ubac2/Gdnf/Rrm2b/Ttll12/Gramd4/Hdac7/Tns2/Heg1/Ezr/Pde10a/Ivns1abp/Lemd2/Rgs8/Plcl2/Tgif1/Lbh/Ltbp1/Socs5/Arhgap12/Sema6a/Adrb2/Tbc1d10c/Fam89b/Lzts2/Sufu/Dusp5/Pdcd4/Dab2ip/Scai/Dlx2/Itga6/Nfe2l2/Dgkz/Mapk8ip1/Cry2/Spred1/Jag1/Ptprt/Stk4/Sulf2/Bmp7/Ift80/Sfrp2/Pear1/Efna1/Adar/Trim32/Dab1/Prkaa2/Trabd2b/Ptprf/Ephb2/Wnt4/Camk2n1/Epha2/Draxin/Mtor/Errfi1/Nphp4/Ski/Htra3/Wfs1/Arhgap24/Pkd2/Fgfrl1/Rasal1/Ncor2/Bmt2/Cyp26b1/Sesn1/Foxo3*	1.514	0.0097	*Rasal1/Ncor2/Sesn1/Foxo3/Pparg/Ift122/Myadm/Ptgir/Bax/Cib1/Ndufc2/Gdpd5/Plekha1/Rnf126/Dusp26/Nkd1/Cx3cl1/Cactin/Egln1/Sesn3/Pin1/Bcl9l/Limd1/Irak3/Prdm14/Nf2/Epn2/Nxn/Igfbp5/Wfikkn2/Socs7/Rgs9/Esr2/Gsc/Dlk1/Lgals3/Nfatc4/Ubac2/Gdnf/Rrm2b/Trp63/Heg1/Pde10a/Lemd2/Plcl2/Lbh/Ltbp1/Sema6a/Lzts2/Sufu/Dusp5/Pdcd4/Tcf7l2/Lefty1/Abl1/Scai/Nfe2l2/Dgkz/Dusp10/Jag1/Sstr4/Ptprt/Mmp9/Bmp7/Ift80/Efna1/Adar/Mcl1/Trim32/Tle1/Dab1/Prkaa2/Trabd2b/Cnksr3/Epha2/Draxin/Mtor/Nphp4/Prkcz/Htra3/Arhgap24*

GO:0051130	positive regulation of cellular component organization	1.57226517	8E-06	*Eps8/Ercc1/Gsk3a/Akt2/Ankrd27/Pdcd5/Nr1h2/Pih1d1/Abcc8/Eed/Syt9/Igf2/Shank2/Map2k7/Ank1/Palld/Crtc1/Pou4f2/Cx3cl1/Has3/Def8/Trim67/Vstm5/Apoa1/Smad3/Nck1/Twf2/Amigo3/Fyco1/Cct2/Nf2/Actr2/Abi2/Srebf1/Arhgef15/Aurkb/Pafah1b1/Asic2/Bcas3/Ngfr/Mien1/Ccr7/Wnt3/Obsl1/Wbp2/Nin/Psen1/Tcl1/Rims1/Actn2/Trim27/Jarid2/Syk/Ror2/Pfn3/Arsb/Iqgap2/Htr1a/Wnt5a/Mapk8/Clasp1/Lgals3/Ptk2b/Npm2/Rgcc/Stk24/Actr3/Rims2/Asap1/Adgrb1/Gpihbp1/Kctd17/Pla2g6/Pdgfb/Rapgef3/Slx4/Ephb3/Cldn1/Syt2/Kalrn/Tiam1/Itsn1/Qk/BC004004/Akap8l/Srf/Dazl/Sh3gl1/Prkce/Lrrtm2/Fchsd1/Cep120/Synpo/Adrb2/Fbxo38/Dcc/Coro1b/Syt7/Vldlr/Camk1d/Pip4k2a/Lrsam1/Dab2ip/Itga6/Nfe2l2/Mapk8ip1/Bmp7/Cdh4/Cct3/Psrc1/5330417C22Rik/Ppp3ca/Cnr1/Mier1/Trabd2b/Ptprf/Foxo6/Ephb2/Wnt4/Epha2/Mtor/Nphp4/Actr3b/Tapt1/C530008M17Rik/Dynll1/Rasal1/Cux2/Fscn1/Ocm/Arpc1a/Plxna4/Lrrtm1/Tgfa/Wasf1/Iqsec1/Fig4/Grip2/Plxnd1/Clstn3*	1.532	0.0049	*Trpv4/Nos1/Rasal1/Cux2/Wasf1/Wasl/Plxna4/Tgfa/Grip2/Pparg/Clstn3/Ercc1/Akt2/Pdcd5/Bax/Abcc8/Myod1/Fes/Syt9/Nrg1/Pou4f2/Cx3cl1/Has3/Wdr61/Sema7a/Smad3/Poc1a/Cck/Ube2n/Fyco1/Cct2/Llph/Nf2/Srebf1/Arhgef15/Dhx33/Asic2/Nme1/Ccr7/Rnf157/Epha4/Tcl1/Rims1/Trim27/Syk/Ice1/Iqgap2/Clasp1/Mapk8/Lgals3/Npm2/Stk24/Asap1/Pla2g6/Syt2/Cldn1/Kalrn/Adamts1/Tiam1/Itsn1/Qk/Mlst8/Akap8l/Sh3gl1/Fchsd1/Cep120/Pmaip1/Bad/Syt7/Vldlr/Pip4k2a/Abl1/Nfe2l2/Mmp9/Bmp7/Cdh4/Ss18l1/Cct3/Ubxn2b/Cnr1/Musk/Trabd2b/Foxo6/Crocc/Epha2/Mtor/Nphp4*
GO:0030334	regulation of cell migration	1.56193773	0.0004	*Akt2/Abcc8/Rhog/Aldoa/Stx4a/Dock1/Igf2/Ccl25/Irs2/Angpt2/Zfp703/Bst2/Cx3cl1/Smad3/Zfp609/Dapk2/Nck1/Elp6/Tmeff2/Abr/Lgals9/Ccl1/Igfbp5/Bcas3/Spag9/Mien1/Ccr7/Itga2b/Prkca/Arhgdia/Rhob/Lgmn/Akt1/Ror2/Arsb/Pik3r1/Ptprg/Wnt5a/Mapk8/Clasp1/Lgals3/Ptk2b/Rhobtb2/Rgcc/Stk24/Ptger4/Actr3/Adgrb1/Card10/Pdgfb/Mgat3/Rapgef3/Hdac7/Cldn1/Lgr6/Tiam1/Lamc2/Srf/Sema6b/Prkce/Jcad/Sema6a/Camk2a/Coro1b/Rhod/Fam89b/Camk1d/Dab2ip/Scai/Itga6/Nfe2l2/Spred1/Frmd5/Jag1/Ptprt/Stk4/Bmp7/Postn/Igsf10/Il12a/Sfrp2/Efna1/Ppp3ca/Lmo4/Fubp1/Glipr2/Epb41l4b/Trim32/Edn2/Wnt4/Rcc2/Epha2/Miip/Mtor/Uts2/Rbpj/Stard13/Plxna4/Iqsec1/Mitf/Foxo3/Plxnd1*	1.657	0.0034	*Trpv4/Foxo3/Wasl/Plxna4/Mitf/Pparg/Myadm/Akt2/Abcc8/Cib1/Ccl25/Angpt2/Nrg1/Dapk3/Cx3cl1/Tmeff2/Sema7a/Smad3/Zfp609/Dapk2/Srgap1/Ccl1/Igfbp5/Spag9/Col1a1/Ccr7/Arhgdia/Pik3r1/Clasp1/Ptprg/Mapk8/Lgals3/Rap2a/Stk24/Gpr183/Ptger4/Mgat3/Lgr6/Cldn1/Adamts1/Lamc2/Tiam1/Aif1/Jcad/Sema6a/Camk2a/Rhod/Abl1/Scai/Nfe2l2/Dusp10/Frmd5/Jag1/Ptprt/Mmp9/Bmp7/Postn/Efna1/Sema6c/Lmo4/Trim32/Edn2/Clic4/Rcc2/Epha2/Miip/Mtor/Uts2/Rbpj*
GO:0044087	regulation of cellular component biogenesis	1.56071653	0.0002	*Eps8/Ercc1/Sptbn4/Lrfn1/Lrfn3/Ankrd27/Nr1h2/Pih1d1/Rhog/Atxn2l/Palld/Cx3cl1/Has3/Def8/Vstm5/Apoa1/Smad3/Nck1/Twf2/Nprl2/Amigo3/Kif9/Vill/Tmeff2/Gm28778/Bbs10/Tbc1d30/Ormdl2/Nf2/Actr2/Abi2/Sept8/Arhgef15/Asic2/Bcas3/Mien1/Ccr7/Cep295nl/Nin/Trim9/Tcl1/Trim27/Syk/Pfn3/Iqgap2/Pik3r1/Vdac2/Wnt5a/Colq/Mapk8/Clasp1/Lgals3/Ptk2b/Rgcc/Ptger4/Myo10/Actr3/Asap1/Adgrb1/Parp10/Kctd17/Pla2g6/Rapgef3/Slx4/Ephb3/Cldn1/Ezr/Srf/Fez2/Prkce/Lrrtm2/Fchsd1/Cep120/Synpo/Coro1b/Rhod/Lrfn4/Slit1/Pip4k2a/Ntng2/Sptan1/Lrsam1/Dab2ip/Bmp7/Chrnb2/Psrc1/5330417C22Rik/Lmo4/Prkaa2/Trabd2b/Ephb2/Wnt4/Rap1gap/Rcc2/Epha2/Mtor/Nphp4/Cptp/Actr3b/Tapt1/C530008M17Rik/Arhgap24/Dynll1/Cux2/Fscn1/Arpc1a/Lrrtm1/Wasf1/Atg7/Clstn3*	1.612	0.073	*Cux2/Wasf1/Wasl/Clstn3/Ercc1/Bax/Fes/Nrg1/Cx3cl1/Has3/Smad3/Cck/Nf2/Arhgef15/Dhx33/Asic2/Ccr7/Tcl1/Trim27/Syk/Ice1/Iqgap2/Clasp1/Mapk8/Lgals3/Asap1/Cldn1/Mlst8/Fchsd1/Cep120/Pip4k2a/Abl1/Bmp7/Musk/Trabd2b/Crocc/Epha2/Mtor/Nphp4*

GO:0044087	regulation of cellular component biogenesis	1.56071653	0.0002	*Eps8/Ercc1/Sptbn4/Lrfn1/Lrfn3/Ankrd27/Nr1h2/Pih1d1/Rhog/Atxn2l/Palld/Cx3cl1/Has3/Def8/Vstm5/Apoa1/Smad3/Nck1/Twf2/Nprl2/Amigo3/Kif9/Vill/Tmeff2/Gm28778/Bbs10/Tbc1d30/Ormdl2/Nf2/Actr2/Abi2/Sept8/Arhgef15/Asic2/Bcas3/Mien1/Ccr7/Cep295nl/Nin/Trim9/Tcl1/Trim27/Syk/Pfn3/Iqgap2/Pik3r1/Vdac2/Wnt5a/Colq/Mapk8/Clasp1/Lgals3/Ptk2b/Rgcc/Ptger4/Myo10/Actr3/Asap1/Adgrb1/Parp10/Kctd17/Pla2g6/Rapgef3/Slx4/Ephb3/Cldn1/Ezr/Srf/Fez2/Prkce/Lrrtm2/Fchsd1/Cep120/Synpo/Coro1b/Rhod/Lrfn4/Slit1/Pip4k2a/Ntng2/Sptan1/Lrsam1/Dab2ip/Bmp7/Chrnb2/Psrc1/5330417C22Rik/Lmo4/Prkaa2/Trabd2b/Ephb2/Wnt4/Rap1gap/Rcc2/Epha2/Mtor/Nphp4/Cptp/Actr3b/Tapt1/C530008M17Rik/Arhgap24/Dynll1/Cux2/Fscn1/Arpc1a/Lrrtm1/Wasf1/Atg7/Clstn3*	1.579	0.0078	*Cux2/Wasf1/Wasl/Noto/Atg7/Clstn3/Myadm/Ercc1/Bax/Fes/Nrg1/Dapk3/Cx3cl1/Has3/Tmeff2/Cryab/Smad3/Stag1/Kif9/Cck/Ormdl2/Nf2/Sept8/Arhgef15/Dhx33/Asic2/Ccr7/Nptx1/Trim9/Tcl1/Trim27/Syk/Ice1/Iqgap2/Pik3r1/Clasp1/Colq/Mapk8/Lgals3/Ptger4/Myo10/Asap1/Pla2g6/Cldn1/Spice1/Mlst8/Fez2/Fchsd1/Sncaip/Cep120/Cep76/Rhod/Pip4k2a/Lefty1/Ntng2/Sptan1/Abl1/Bmp7/Chrnb2/Lmo4/Odf2l/Musk/Prkaa2/Trabd2b/Rcc2/Crocc/Epha2/Mtor/Nphp4/Prkcz/Arhgap24*
GO:0045595	regulation of cell differentiation	1.54364775	3E-08	*Sox5/Brsk1/Foxa3/Ppp1r13l/Gsk3a/Nfkbid/Hpn/Ankrd27/Sult2b1/Abcc8/Klf13/Gdpd5/Arntl/Nucb2/Dock1/Nkx6-2/Igf2/Shank2/Zfp703/Pcm1/Crtc1/Pou4f2/Cacna1a/Cx3cl1/Ccl17/Zfpm1/Trim67/Pin1/S1pr5/Zbtb7a/Bcl9l/Apoa1/Tle6/Smad3/Zfp609/Nedd4/Rbp1/Nck1/Ryk/Twf2/Amigo3/Limd1/Pa2g4/Nf2/Actr2/Abi2/Irf1/Pmp22/Rap1gap2/Pafah1b1/Lgals9/Igfbp5/Mmd/Spag9/Tob1/Ngfr/Rara/Ccr7/Wnt3/Obsl1/Mafg/Id2/Nin/Psen1/Fos/Bcl11b/Rims1/Akt1/Syk/Ror2/Arsb/Ap3b1/Cartpt/Pik3r1/Nln/Ptprg/Rarb/Zswim8/Wnt5a/Mapk8/Gdf10/Clasp1/Zfp219/Prmt5/Cmtm5/Wdfy2/Gli2/Ptk2b/Rgcc/Stk24/Ptger4/Gdnf/Actr3/Rims2/Asap1/Adgrb1/Triobp/Maff/Pdgfb/Nfam1/Slc45a3/Hdac7/Vdr/Ephb3/Etv5/Syt2/Kalrn/Tiam1/Itsn1/Runx1/Qk/Spdef/Sik1/Notch3/H2-Aa/Tnfrsf21/Srf/Sema6b/Trip10/Tgif1/Lbh/Sos1/Tmem178/Socs5/Hspa9/Sema6a/Camk2a/Ppargc1b/Fbxo38/Malt1/Tcf4/Dcc/Zbtb7c/Smad2/Vldlr/Slit1/Pdcd4/Adrb1/Vax1/Camk1d/Pax8/Olfm1/Dab2ip/Dlx2/Itga6/Nfe2l2/Lmo2/Eif2ak4/Jag1/Eif6/Mafb/Zhx3/Stk4/Bmp7/Cdh4/Adrm1/Postn/Igsf10/Il12a/Sfrp2/Efna1/Chrnb2/Rbm15/Slc9b2/Ppp3ca/Eif4e/Bmpr1b/Lmo4/Fubp1/Cnr1/Glipr2/Trim32/Dab1/Ptch2/Ptprf/Foxo6/Sfn/Runx3/Ephb2/Wnt4/Rap1gap/Rcc2/Spen/Tnfrsf1b/Draxin/Disp3/Mtor/Errfi1/Uts2/Ajap1/Hes5/Ski/Smarcd3/Adra2c/Rbpj/Phox2b/Map3k5/Rasal1/Cux2/Rflna/Ncor2/Ywhag/Ocm/Plxna4/Cyp26b1/Fig4/Grip2/Mitf/Atg7/Foxo3/Plxnd1/Gnb3*	1.507	0.0003	*Trpv4/Nos1/Rasal1/Cux2/Ncor2/Foxo3/Card11/Plxna4/Dguok/Grip2/Mitf/Atg7/Pparg/Myadm/Brsk1/Foxa3/Ppp1r13l/Hpn/Abcc8/Myod1/Klf13/Cib1/Fes/Gdpd5/Rassf10/Nkx6-2/Nrg1/Pcm1/Pou4f2/Fto/Cx3cl1/Zfpm1/Pin1/S1pr5/Bcl9l/Wdr61/Sema7a/Smad3/Zfp609/Limd1/Llph/Nf2/Igfbp5/Mmd/Nme1/Spag9/Col1a1/Ccr7/Rnf157/Epha4/Esrrb/Rims1/Mylip/Syk/Spock1/Ap3b1/Pik3r1/Clasp1/Ptprg/Rarb/Mapk8/Acin1/Cmtm5/Nfatc4/Rap2a/Stk24/Gpr183/Ptger4/Gdnf/Asap1/Triobp/Syt2/Vdr/Trp63/Kalrn/Adamts1/Tiam1/Itsn1/Qk/Notch3/Trip10/Lbh/Tmem178/Sema6a/Camk2a/Ppargc1b/Malt1/Bad/Vldlr/Pdcd4/Tcf7l2/Adrb1/Vax1/Abl1/Nfe2l2/Lmo2/Dusp10/Jag1/Mafb/Mmp9/Bmp7/Cdh4/Ss18l1/Adrm1/Postn/Efna1/Chrnb2/Sema6c/Slc9b2/Eif4e/Bmpr1b/Lmo4/Cnr1/Musk/Trim32/Dab1/Dmrta2/Foxo6/Runx3/Rcc2/Spen/Tnfrsf1b/Draxin/Mtor/Casz1*

GO:0035556	intracellular signal transduction	1.53016754	1E-05	*Eps8/Brsk1/Calm3/Relb/Akt2/Mef2a/Eif4ebp2/Pde2a/Rhog/Tead1/Nucb2/Atp2a1/Dock1/Inpp5a/Map2k7/Myo16/Mcf2l/Tmem38a/Pou4f2/Def8/Gucy1a2/Sesn3/Rab3d/Arhgef12/Nlrx1/Fzr1/Dapk2/Mras/Twf2/Shisa5/Plcd1/Snrk/Gm5136/Irak3/Ramp3/Grb10/Abi2/Rasgef1c/Irf1/Nmur2/Abr/Igfbp5/Ngfr/Kpnb1/Socs7/Ccr7/Prkca/Prkar1a/Arhgdia/Rab10/Adcy3/Rhob/Psen1/Dicer1/Cdc42bpb/Akt1/Gdi2/Syk/Ror2/Rasgrf2/Pik3r1/Mast4/Depdc1b/Wnt5a/Sh3bp5/Ercc6/Mapk8/Rab2b/Spata13/Ptk2b/Rhobtb2/Stk24/Dock9/Ptger4/Ncald/Rims2/Adgrb1/Nrbp2/Pdgfb/Atf4/Nfam1/Rapgef3/Racgap1/Tns2/Tfap4/Fgf12/Kalrn/Adcy5/Arhgap31/Tiam1/Hunk/Itsn1/Ezr/Sik1/Rab11b/Rcan2/Plcl2/Ralbp1/Sos1/Map4k3/Prkce/Socs5/Msh2/Rab18/Adrb2/Malt1/Smad2/Coro1b/Rhod/Rasgrp2/Adrb1/Rab14/Dab2ip/Dgkz/Psen2/Ghrh/Stk4/Mc3r/Pear1/Ank2/Ppp3ca/Bcl10/Ptgfr/Xpa/Acer2/Dab1/Prkaa2/Kcnh1/Edn2/Sfn/Epha2/Casp9/Tnfrsf1b/Mtor/Errfi1/Plch2/Prkag2/Htr5a/Rab32/Adra2c/Stk32b/Mapk10/Map3k5/Wsb2/Rasal1/Lat2/Prkar1b/Dgki/Adcyap1r1/Kbtbd2/Wasf1/Foxo3/Gapdh*	1.568	0.0009	*Ksr2/Nos1/Rasal1/Wasf1/Lat2/Foxo3/Prkar1b/Card11/Dgki/Chn2/Adcyap1r1/Kbtbd2/Ift122/Gapdh/Pik3c2g/Brsk1/Ptgir/Akt2/Bax/Atp2a1/Plekha1/Myo16/Mcf2l/Nrg1/Tmem38a/Pou4f2/Dapk3/Sesn3/Cryab/Dapk2/Apaf1/Mapkapk3/Snrk/Gm5136/Irak3/Srgap1/Rhbdd3/Rasgef1c/Igfbp5/Kpnb1/Socs7/Ccr7/Rgs9/Prkar1a/Arhgdia/Ywhaq/Gphb5/Cdc42bpb/Gdi2/Ramp1/Syk/Pik3r1/Depdc1b/Arhgef3/Mapk8/Nfatc4/Rap2a/Stk24/Dock9/Ptger4/Rabif/Racgap1/Trp63/Kalrn/Tiam1/Hunk/Itsn1/Arhgef4/Mlst8/Aif1/Rcan2/Plcl2/Map4k3/Lhcgr/Malt1/Pmaip1/Rps6kb2/Rhod/Map3k11/Rasgrp2/Bad/Adrb1/Abl1/Dgkz/Plcb4/Rab33b/Mcl1/Acer2/Dab1/Prkaa2/Acot11/Edn2/Epha2/Tnfrsf1b/Mtor/Prkcz/Htr5a/Adra2c/Stk32b/Mapk10*
GO:0040012	regulation of locomotion	1.52995345	0.0004	*Akt2/Abcc8/Il16/Rhog/Aldoa/Stx4a/Dock1/Igf2/Ccl25/Irs2/Angpt2/Zfp703/Bst2/Pou4f2/Cx3cl1/Pin1/Smad3/Zfp609/Dapk2/Nck1/Ryk/Elp6/Tmeff2/Abr/Lgals9/Ccl1/Igfbp5/Bcas3/Spag9/Mien1/Ccr7/Itga2b/Wnt3/Prkca/Arhgdia/Rhob/Lgmn/Akt1/Ror2/Arsb/Pik3r1/Ptprg/Zswim8/Wnt5a/Mapk8/Clasp1/Lgals3/Ptk2b/Rhobtb2/Rgcc/Stk24/Ptger4/Actr3/Adgrb1/Card10/Pdgfb/Mgat3/Rapgef3/Hdac7/Cldn1/Lgr6/Tiam1/Lamc2/Srf/Sema6b/Prkce/Jcad/Sema6a/Camk2a/Coro1b/Rhod/Fam89b/Chrm1/Slit1/Camk1d/Dab2ip/Scai/Itga6/Nfe2l2/Spred1/Frmd5/Jag1/Ptprt/Stk4/Bmp7/Postn/Igsf10/Il12a/Sfrp2/Efna1/Ppp3ca/Lmo4/Fubp1/Glipr2/Epb41l4b/Trim32/Edn2/Wnt4/Rcc2/Epha2/Miip/Mtor/Uts2/Rbpj/Ocm/Stard13/Plxna4/Iqsec1/Mitf/Foxo3/Plxnd1*	1.599	0.0048	*Trpv4/Foxo3/Wasl/Plxna4/Mitf/Pparg/Myadm/Akt2/Abcc8/Cib1/Fes/Il16/Ccl25/Angpt2/Nrg1/Pou4f2/Dapk3/Cx3cl1/Pin1/Tmeff2/Sema7a/Smad3/Zfp609/Dapk2/Srgap1/Prdm14/Ccl1/Igfbp5/Spag9/Col1a1/Ccr7/Arhgdia/Pik3r1/Clasp1/Ptprg/Mapk8/Lgals3/Rap2a/Stk24/Gpr183/Ptger4/Mgat3/Lgr6/Cldn1/Adamts1/Lamc2/Tiam1/Aif1/Jcad/Sema6a/Camk2a/Rhod/Abl1/Scai/Nfe2l2/Dusp10/Frmd5/Jag1/Ptprt/Mmp9/Bmp7/Postn/Efna1/Sema6c/Lmo4/Trim32/Edn2/Clic4/Rcc2/Epha2/Miip/Mtor/Uts2/Rbpj*

GO:0051270	regulation of cellular component movement	1.5274757	0.0003	*Akt2/Abcc8/Rhog/Ctnna3/Atp2a1/Aldoa/Stx4a/Dock1/Igf2/Ccl25/Irs2/Angpt2/Zfp703/Bst2/Pou4f2/Cx3cl1/Pin1/Smad3/Zfp609/Dapk2/Nck1/Ryk/Elp6/Tmeff2/Abr/Lgals9/Ccl1/Igfbp5/Bcas3/Spag9/Mien1/Ccr7/Itga2b/Wnt3/Prkca/Arhgdia/Rhob/Actn1/Lgmn/Akt1/Ror2/Arsb/Pik3r1/Ptprg/Zswim8/Wnt5a/Mapk8/Clasp1/Lgals3/Ptk2b/Rhobtb2/Rgcc/Stk24/Ptger4/Actr3/Adgrb1/Card10/Pdgfb/Mgat3/Rapgef3/Hdac7/Cldn1/Lgr6/Tiam1/Tnnt2/Lamc2/Srf/Sema6b/Prkce/Jcad/Sema6a/Camk2a/Coro1b/Rhod/Fam89b/Slit1/Adrb1/Camk1d/Dab2ip/Scai/Itga6/Nfe2l2/Spred1/Frmd5/Jag1/Ptprt/Stk4/Bmp7/Postn/Igsf10/Il12a/Sfrp2/Efna1/Ank2/Ppp3ca/Lmo4/Fubp1/Glipr2/Epb41l4b/Trim32/Edn2/Wnt4/Rcc2/Epha2/Miip/Mtor/Uts2/Rbpj/Ocm/Stard13/Plxna4/Iqsec1/Mitf/Foxo3/Plxnd1*	1.604	0.0034	*Trpv4/Nos1/Foxo3/Wasl/Plxna4/Mitf/Pparg/Ctnna3/Myadm/Akt2/Abcc8/Cib1/Fes/Atp2a1/Ccl25/Angpt2/Nrg1/Pou4f2/Dapk3/Cx3cl1/Pin1/Tmeff2/Sema7a/Smad3/Zfp609/Dapk2/Srgap1/Prdm14/Ccl1/Igfbp5/Spag9/Col1a1/Ccr7/Arhgdia/Pik3r1/Clasp1/Ptprg/Mapk8/Lgals3/Rap2a/Stk24/Gpr183/Ptger4/Mgat3/Lgr6/Cldn1/Adamts1/Lamc2/Tiam1/Aif1/Jcad/Sema6a/Camk2a/Rhod/Adrb1/Abl1/Scai/Nfe2l2/Dusp10/Frmd5/Jag1/Ptprt/Mmp9/Bmp7/Postn/Efna1/Sema6c/Lmo4/Trim32/Edn2/Clic4/Rcc2/Epha2/Miip/Mtor/Uts2/Rbpj*
GO:0060341	regulation of cellular localization	1.50671637	0.0005	*Calm3/Grik5/Gsk3a/Akt2/Ryr1/Pdcd5/Apba2/Rhog/Syt9/Atp2a1/Stx4a/Ank3/Mrln/Pcm1/Pde4c/Tmem38a/Gipc1/Cacna1a/Cx3cl1/Necab2/Reep6/Pin1/Zpr1/Nedd4/Plcd1/Emd/Cct2/Nf2/Ramp3/Sept8/Srebf1/Stx8/Aurkb/Abr/Lgals9/Bcas3/Pnkd/Ngfr/Agr2/Psen1/Akt1/Actn2/Nrn1/Syk/Pde8b/Pik3r1/Htr1a/Wnt5a/Lgals3/Ptk2b/Ubac2/Ptger4/Gdnf/Actr3/Rims2/Card10/Pla2g6/Rapgef3/Asic1/Kalrn/Adcy5/Stxbp5l/Tiam1/Tnnt2/Ezr/Btbd9/Akap8l/Pram1/Rab11b/Plcl2/Sh3gl1/Rab23/Prkce/Cript/Camk2a/Sh3tc2/Adrb2/Chrm1/Syt7/Apba1/Lzts2/Adrb1/Nos1ap/Cnst/Map1a/Kcnb1/Cct3/Chrnb2/Ank2/Slc9b2/Ptpn14/Cnr1/Prkaa2/Scp2/Sfn/Ephb2/Htr6/Epha2/Plch2/Kcnh2/Pkd2/Cabp1/Dynll1/Tpcn1/Prkar1b/Actb/Dgki/Adcyap1r1/Reep1/Iqsec1/Chchd4/Grip2/Gpr27/Edem1/Atg7/Snx3*	1.711	0.0003	*Nos1/Prkar1b/Actb/Dgki/Adcyap1r1/Chchd4/Grip2/Edem1/Atg7/Vamp1/Grik5/Akt2/Mrln/Pdcd5/Bax/Apba2/Cib1/Fes/Syt9/Atp2a1/Pcm1/Pde4c/Tmem38a/Cx3cl1/Jph3/Pin1/Zpr1/Cryab/Snx12/Cep290/Cct2/Nf2/Sept8/Srebf1/Stx8/Cltc/Pnkd/Agr2/Vti1b/Nrn1/Syk/Ice1/Pik3r1/Lgals3/Ubac2/Ptger4/Gdnf/Pla2g6/Pim3/Kalrn/Tiam1/BC034090/Akap8l/Pram1/Plcl2/Sh3gl1/Cript/Lhcgr/Sncaip/Camk2a/Sh3tc2/Pmaip1/Nos1ap/Bad/Syt7/Lzts2/Trim8/Tcf7l2/Adrb1/Abl1/Map1a/Plcb4/Snta1/Pard6b/Cct3/Chrnb2/Slc9b2/Ubxn2b/Cnr1/Musk/Prkaa2/Crocc/Epha2/Prkcz*

Enrichment ＞1.5, FDR ＜0.05

Table S5

Identified lactylated histone proteins between sham group and MCAO group (n = 3)

Accession	Position	Amino acid	Gene name	MCAO1	MCAO2	MCAO3	Sham1	Sham2	Sham3	Relative level of lactylated protein in MCAO compared to Sham	
										Fold change	P value
Q3THW5	116	K	*H2az2*	-	-	-	1.137	-	-	-	-
Q64478	44	K	*H2bc9*	-	-	-	-	-	-	-	-
Q64478	47	K	*H2bc9*	-	-	-	-	-	-	-	-
Q64478	117	K	*H2bc9*	-	-	-	-	-	-	-	-
Q64478	35	K	*H2bc9*	-	-	-	-	-	-	-	-
Q64478	109	K	*H2bc9*	-	-	-	-	-	-	-	-
P10854	6	K	*H2bc14*	-	-	-	-	-	-	-	-
P27661	128	K	*H2ax*	0.769	1.314	1.061	1.005	0.924	-	1.087	0.786469116
P62806	78	K	*H4c1*	0.903	1.563	1.257	0.42	0.817	1.269	1.486	0.261895641
P62806	32	K	*H4c1*	1.005	1.45	1.126	0.541	0.821	1.22	1.387	0.226389393
P62806	80	K	*H4c1*	0.987	1.868	1.313	0.523	0.689	0.945	1.932	0.060076592
P62806	92	K	*H4c1*	0.969	1.275	0.973	-	1.024	-	1.047	-
P84244	80	K	*H3-3a*	0.825	0.891	1.171	0.855	1.011	1.338	0.901	0.590137133
P84244	24	K	*H3-3a*	0.79	1.021	1.093	0.883	1.005	1.297	0.912	0.585596772
P84244	123	K	*H3-3a*	0.852	1.423	-	0.677	0.719	1.264	1.283	0.47886682
P84244	57	K	*H3-3a*	-	1.28	1.047	0.623	0.936	1.349	1.2	0.502197177
Q64475	6	K	*H2bc3*	-	-	-	-	-	-	-	-
Q64478	6	K	*H2bc9*	-	-	-	-	-	-	-	-
Q64524	6	K	*H2bc21*	-	-	-	-	-	-	-	-
Q64525	6	K	*Hist2h2bb*	-	-	-	-	-	-	-	-
Q8CGP0	6	K	*H2bu1*	-	-	-	-	-	-	-	-

Table S6

Information of frozen postmortem human brain samples, related to Figure S2E

	Age	Gender	Date of Death	Date of Biopsy	Cause of Death
Stroke	55	Male	17-Apr-19	18-Apr-19	Ischemic Stroke

Table S7

A list of primer or probes, related to Experimental Section.

Applications	Primers or genes	Forward	Reverse
qPCR	Pdcd4	AAAGACGACTGCGGAAAAATTCA	CTTCTAACCGCTTCACTTCCATT
	Pla2g6	AAGCCAGCGTTCATCCTGAGCT	TCGATGGCGATGAGAAGCTGGA
	β-Actin	ACGGCCAGGTCATCACTATT	TGGCATAGAGGTCTTTACGGA
qCHIP	Pdcd4-peak4099	CTTAACCCCCACTCTGGCTG	GCTAGGGGAAGTGAGACGTG
	Pla2g6-peak2736	TCACTCACCTCGGCTTACTCAGCACTTCTC	GGCATGGGCTCGGTGGATTGGT
Mouse genotyping	Mecp-WT	TGGGCTAAATGACTTGCAGGTC	TGCTAACTGGGGATCTCTGCTAC
	Mecp2-KO	CTTGAGAAGTTGTGACTTTGCAGG	GGTCAACAGCTTGTCTGGTCAGT
	Mecp2-Loxp	GACATTGTTTCATCTTCCATGCCA	TTTGTCAGAGCCCTACCCATAAG
	Syn1-cre	AGCAGAGGAGTCGCGTCGTG	CGCAGCAGGGTGTTGTAGGC
	Syn1-WT	AGTCTTTCCCTTGCCTCTGCT	GGGTCTTCCACCTTTCTTCAG
Primers for EMSA probes	mouse-Pdcd4 mCpG probe	G(5-Methyl dC)CGCCC(5-Methyl dC)CGCCCCCCTGAGCCC(5-Methyl dC)CGCCCCT	AGGGG(5-Methyl dC)CGGGGCTCAGGGGGG(5-Methyl dC)CGGGG(5-Methyl dC)CGC
	mouse-Pdcd4 probe	GCGCCCCGCCCCCCTGAGCCCCGCCCCT	AGGGGCGGGGCTCAGGGGGGCGGGGCGC
	mouse-Pla2g6 mCpG probe	AGGCCC(5-Methyl dC)CGCCCC(5-Methyl dC)CGC(5-Methyl dC)CGCCC(5-Methyl dC)CGCCCA(5-Methyl dC)CGT	A(5-Methyl dC)CGTGGG(5-Methyl dC)CGGGG(5-Methyl dC)CGG(5-Methyl dC)CGGGGG(5-Methyl dC)CGGGGCCT
	mouse-Pla2g6 probe	AGGCCCCGCCCCGCCGCCCCGCCCACGT	ACGTGGGCGGGGCGGCGGGGGCGGGGCCT
	human-Pdcd4 mCpG probe	G(5-Methyl dC)CGCATG(5-Methyl dC)CGCCCTCC(5-Methyl dC)CG(5-Methyl dC)CGCCCCTCCCC	GGGGAGGGG(5-Methyl dC)CG(5-Methyl dC)CGGGAGGG(5-Methyl dC)CGCATG(5-Methyl dC)CGC
	human-Pdcd4 probe	GCGCATGCGCCCTCCCGCGCCCCTCCCC	GGGGAGGGGCGCGGGAGGGCGCATGCGC
	human-Pla2g6 mCpG probe	G(5-Methyl dC)CGCATG(5-Methyl dC)CGCCCTCC(5-Methyl dC)CG(5-Methyl dC)CGCCCCTCCCC	GGGGAGGGG(5-Methyl dC)CG(5-Methyl dC)CGGGAGGG(5-Methyl dC)CGCATG(5-Methyl dC)CGC
	human-Pla2g6 probe	GCGCATGCGCCCTCCCGCGCCCCTCCCC	GGGGAGGGGCGCGGGAGGGCGCATGCGC
shRNA targeting sequence	Hdac1-shRNA1	GCTTGGGTAATAGCAGCCATT	
	Hdac1-shRNA2	GCCAGTCATGTCCAAAGTAAT	
	Hdac2-shRNA1	CCCAATGAGTTGCCATATAAT	
	Hdac2-shRNA2	CGAGCATCAGACAAACGGATA	
	Hdac3-shRNA1	CCTGCATTATGGTCTCTATAA	
	Hdac3-shRNA2	GTGTTGAATATGTCAAGAGTT	
	P300-shRNA1	CCCTGGATTAAGTTTGATAAA	
	P300-shRNA2	CCAACAGGAATGACTACCAAT	
	AARS1-shRNA1	GCTGCACATAGGAACGATATA	
	AARS1-shRNA2	GACCTCATCATGCTGGACATT	
	GCN5-shRNA1	GAAGCCTTCTACGGTCCATTT	
	GCN5-shRNA2	CGTGCCAAGAAGCTTGAGAAA	
	CBP-shRNA1	CGCGAATGACAACACAGATTT	
	CBP-shRNA2	TAACTCTGGCCATAGCTTAAT	
	PCAF-shRNA1	CTCTTGAGAAACGCACGCTTA	
	PCAF-shRNA2	CCGGGATATTATGAAGTTATA	
